# Effect of Internet-Based Cognitive Behavioral Humanistic and Interpersonal Training vs Internet-Based General Health Education on Adolescent Depression in Primary Care: A Randomized Clinical Trial

**DOI:** 10.1001/jamanetworkopen.2018.4278

**Published:** 2018-11-02

**Authors:** Tracy R. G. Gladstone, Daniela A. Terrizzi, Allison Paulson, Jennifer Nidetz, Jason Canel, Eumene Ching, Anita D. Berry, James Cantorna, Joshua Fogel, Milton Eder, Megan Bolotin, Lauren O. Thomann, Kathy Griffiths, Patrick Ip, David A. Aaby, C. Hendricks Brown, William Beardslee, Carl Bell, Theodore J. Crawford, Marian Fitzgibbon, Linda Schiffer, Nina Liu, Monika Marko-Holguin, Benjamin W. Van Voorhees

**Affiliations:** 1 The Robert S. and Grace W. Stone Primary Prevention Initiatives, Wellesley Centers for Women, Wellesley College, Wellesley, Massachusetts; 2 Department of Pediatrics, College of Medicine, University of Illinois at Chicago, Chicago; 3 School of Social Service Administration, University of Chicago, Chicago, Illinois; 4 NorthShore University HealthSystem, Evanston, Illinois; 5 Harvard Vanguard, Boston, Massachusetts; 6 Advocate Children’s Hospital, Downers Grove, Illinois; 7 Franciscan Medical Specialists, Munster, Indiana; 8 Department of Business Management, Brooklyn College, Brooklyn, New York; 9 Department of Family Medicine and Community Health, University of Minnesota, Minneapolis; 10 Research School of Psychology, College of Health & Medicine, The Australian National University, Canberra, Australia; 11 Department of Paediatrics and Adolescent Medicine, Queen Mary Hospital, The University of Hong Kong, Pokfulam, Hong Kong; 12 Department of Preventive Medicine, Feinberg School of Medicine, Northwestern University, Chicago, Illinois; 13 Department of Psychiatry and Behavioral Sciences, Feinberg School of Medicine, Northwestern University, Chicago, Illinois; 14 Judge Baker Center, Harvard Medical School, Boston, Massachusetts; 15 Jackson Park Hospital, Chicago, Illinois; 16 Department of Psychiatry, School of Medicine, University of Illinois at Chicago, Chicago; 17 Windsor University, St Kitts, St Kitts and Nevis; 18 Graduate School of Social Work, Chicago State University and Positive Influence, Inc, Chicago, Illinois; 19 University of Illinois Cancer Center, University of Illinois at Chicago, Chicago; 20 Institute for Health Research and Policy, School of Public Health, University of Illinois at Chicago, Chicago

## Abstract

**Importance:**

Although 13% to 20% of American adolescents experience a depressive episode annually, no scalable primary care model for adolescent depression prevention is currently available.

**Objective:**

To study whether competent adulthood transition with cognitive behavioral humanistic and interpersonal training (CATCH-IT) lowers the hazard for depression in at-risk adolescents identified in primary care, as compared with a general health education (HE) attention control.

**Design, Setting, and Participants:**

This multicenter, randomized clinical trial, a phase 3 single-blind study, compares CATCH-IT with HE. Participants were enrolled from 2012 to 2016 and assessed at 2, 6, 12, 18, and 24 months postrandomization in a primary care setting. Eligible adolescents were aged 13 to 18 years with subsyndromal depression and/or history of depression and no current depression diagnosis or treatment. Of 2250 adolescents screened for eligibility, 446 participants completed the baseline interview, and 369 were randomized into CATCH-IT (n = 193) and HE (n = 176).

**Interventions:**

The internet-based intervention, CATCH-IT, is a 20-module (15 adolescent modules and 5 parent modules) online psychoeducation course that includes a parent program, supported by 3 motivational interviews.

**Main Outcomes and Measures:**

Time to event for depressive episode; depressive symptoms at 6 months.

**Results:**

Of 369 participants (mean [SD] age, 15.4 [1.5] years; 251 women [68%]) included in this trial, 193 were randomized into CATCH-IT and 176 into HE. Among these participants, 28% had both a past episode and subsyndromal depression; 12% had a past episode only, 59% had subsyndromal depression only, and 1% had borderline subsyndromal depression. The outcome of time to event favored CATCH-IT but was not significant with intention-to-treat analyses (unadjusted hazard ratio [HR], 0.59; 95% CI, 0.27-1.29; *P* = .18; adjusted HR, 0.53; 95% CI, 0.23-1.23; *P* = .14). Adolescents with higher baseline Center for Epidemiologic Studies Depression scale (CES-D_10_) scores showed a significantly stronger effect of CATCH-IT on time to event relative to those with lower baseline scores (HR 0.82; 95% CI, 0.67-0.99; *P* = .04). For example, the hazard ratio for a CES-D_10_ score of 15 was 0.20 (95% CI, 0.05-0.77), compared with a hazard ratio of 1.44 (95% CI, 0.41-5.03) for a CES-D_10_ score of 5. In both CATCH-IT and HE groups, depression symptoms declined and functional scores increased.

**Conclusions and Relevance:**

For preventing depressive episodes CATCH-IT may be better than HE for at-risk adolescents with subsyndromal depression. Also CATCH-IT may be a scalable approach to prevent depressive episodes in adolescents in primary care.

**Trial Registration:**

ClinicalTrials.gov Identifier: NCT01893749

## Key Points

**Questions:**

Does an internet-based depression prevention program (competent adulthood transition with cognitive behavioral humanistic and interpersonal training) lower the hazard for depression in at-risk adolescents relative to health education attention control?

**Findings:**

In this randomized clinical trial of adolescents with subsyndromal depression or history of depression randomized to receive internet-based behavioral humanistic interpersonal training or an internet-based general health education control, those who received the CATCH-IT intervention did not evidence fewer episodes of depression in the full intention-to-treat sample, but adolescents with subsyndromal depression may have experienced fewer depressive episodes.

**Meaning:**

Competent adulthood transition with cognitive behavioral humanistic and interpersonal training may be better than health education for preventing depression in adolescents with subsyndromal depression.

## Teaser

This randomized clinical trial evaluates the effect of cognitive behavioral humanistic and interpersonal training vs a general health education attention control on the risk of depression in at-risk adolescents identified in primary care.

## Introduction

Approximately 13% to 20% of adolescents experience minor depressive episodes (mDE) or major depressive episodes (MDE) annually.^1^ These adolescents have a higher incidence of medical illness^2^ than those without mDE and MDE, and are at higher risk for suicide and recurrent depression.^3,4,5^ Effective depression prevention programs are essential.^6^

Promising findings for depression prevention programs are available. A Cochrane meta-analysis of prevention trials favored the intervention group over the control group with an overall risk difference for depressive disorders of −0.03, and for depression symptoms a standard mean difference of −0.21.^7^ A review noted a 22% risk reduction of depressive episodes for adolescents.^7,8^ Another meta-analysis involving 19 randomized preventive trials demonstrated significant reduction in depressive symptoms over 2 years among adolescents with higher symptom levels.^9^ Another review of traditional therapies augmented with computerized communications demonstrated small-to-moderate effect sizes for depressive symptoms.^10^ A systematic review of primary care-based preventative interventions targeting depression identified 14 randomized clinical trials; only 1 included adolescents, and average effect sizes were small.^11^ Targeted interventions that show success during trials may not be scalable owing to practical issues such as cost, or prove ineffective in the broader community.^12^

The primary care internet-based intervention, competent adulthood transition with cognitive behavioral humanistic and interpersonal training (CATCH-IT) addresses the need for a scalable intervention.^13,14,15,16^ Internet-based interventions are accessible, cost-effective, private, and acceptable because they reduce stigma.^12^ The CATCH-IT intervention is simple, consumer friendly, and more easily scaled up than more intensive, face-to-face interventions. A randomized clinical trial in China found that CATCH-IT lowered depressive symptoms in adolescents over 12 months.^17^

We present a multisite randomized clinical trial testing the efficacy of CATCH-IT (version 3) vs an internet-based health education (HE) attention control in primary care. We aimed to prevent the onset of depressive episodes and lower symptoms in adolescents at intermediate-to-high risk for depression. Our primary hypothesis was that adolescents assigned to CATCH-IT relative to HE would have a lower hazard ratio (HR) for mDE or MDE at 6 months. We chose to evaluate group differences at 6 months to examine the potential of CATCH-IT as an immediate, medium-term response to depressive symptoms, given that follow-up intervals for such interventions in adolescents generally range from less than 6 to 12 months.^12^ We also hypothesize that adolescents in CATCH-IT would show improvement in depressed mood and functional status relative to HE.^18^

## Methods

### Study Design and Setting

We conducted a hybrid type 1 effectiveness-implementation trial to test the efficacy of CATCH-IT in a scalable setting and collected information regarding implementation.^19^ This 2-site (Chicago, Illinois, and Boston, Massachusetts) randomized trial compared CATCH-IT vs HE for preventing depressive episode onset in an intermediate- to high-risk sample of adolescents in primary care. We defined risk status as teens’ current elevated symptoms of depression, history of depressive episode, or both. Depressive episodes are defined as a Depression Severity Rating (DSR) of 3 or more (exhibiting symptoms of subthreshold MDE). At baseline, the participants’ average Center for Epidemiologic Studies Depression scale (CES-D_20_) score was 16.9. Twelve percent of the sample enrolled with a past MDE only, 60% had current elevated symptoms only, and 28% had both a past MDE and elevated symptoms of depression. Participants were assessed at baseline and at 2 and 6 months postenrollment. Dates of depressive episodes were recorded. Depressive episodes were diagnosed through the use of Kiddie Schedule for Affective Disorders scale (K-SADS) interviews.

Institutional review board approval was received from the central site, University of Illinois at Chicago, and local institutional review boards (IRB of Record, Wellesley College, Advocate Healthcare, Franciscan Alliance, Northshore University Health Systems, Northwestern, and Access Healthcare).^20^ Participants were recruited from 2012 to 2016 through a description of the study during doctor visits, recruitment letters, and posted flyers. Adolescents were screened for risk in-person or by phone. After parental consent, adolescents participated in an eligibility assessment by phone. The parent and adolescent attended an enrollment assessment at their primary care office, when written informed consent from parents and assent from adolescents were obtained, and assessments were administered to confirm eligibility.^21^ This study followed the Consolidated Standards of Reporting Trials (CONSORT) reporting guideline. The protocol, implementation process, and methods have been described in Supplement 1 and elsewhere.^18^ The study was conducted in clinics in Chicago, northern Indiana, and Boston.

### Inclusion and Exclusion Criteria

Adolescents aged 13 to 18 years with elevated levels of depressive symptoms on the CES-D^22^ (scores 8-17 on the CES-D_10_ or scores ≥16 on the CES-D_20_) at screening or at baseline, and/or a history of depression or dysthymia,^22,23,24^ were eligible. Exclusion criteria included the following: current MDE diagnosis or treatment; past cognitive behavioral therapy; CES-D_10_ scores of more than 17^22^; schizophrenia, psychosis, or bipolar disorder; serious medical condition (ie, causing serious disability or dysfunction); significant reading impairment or developmental disability; imminent suicidal risk; and current drug or alcohol abuse.^25,26^ Criteria were selected to avoid confounding factors in depression etiology or treatment, consistent with the use of CATCH-IT as a preventive intervention.

### Randomization

Participants were assigned randomly to CATCH-IT or HE (1:1 allocation) using a computer generated sequence blocked by site and time of entry (random blocks of size 4 and 6), stratified by risk severity (based on CES-D score, prior MDE, or dysthymia), sex and age (13-14 years or 15-18 years).^26,27^

### Blinding

Randomization was concealed from investigators, clinicians, patients, and families until the baseline consent, enrollment, data collection, and assessment were completed. Study participants could not be blinded to their arm assignment. The health care professional was also not blinded, as he or she was expected to conduct 3 motivational interviews (MIs) for CATCH-IT participants. Assessors remained blinded throughout the study. Principal investigators (B.W.V.V. and T.R.G.G.) were blinded to between-group comparisons and group descriptive data until all 6-month follow-up data were collected.

### Retention

Challenges related to ongoing study participation were addressed by research staff. Approaches used to maintain contact were birthday cards and regular contact updates.

### Outcomes

Occurrence of first depressive episode was determined by the DSR. A score indicating at least subthreshold major depression (a DSR of ≥3) was considered to be a depressive episode. To test for robustness of findings, we also examined data using a DSR cutoff of 4 or more, indicating probable MDE, and a DSR of 5, indicating the presence of MDE.^28^ Symptom outcomes include the CES-D_10_^22^ and Global Assessment Scale (GAS) scores.

### CATCH-IT Intervention

The CATCH-IT intervention includes an internet component (15 adolescent modules, based primarily on the Coping with Depression Adolescent Course,^29^ behavioral activation,^30^ and interpersonal psychotherapy),^31^ a brief motivational component (3 physician MIs at 0, 2, and 12 months), and 1 to 3 staff coaching phone calls either at 1 month (Chicago) or at 2 and 4 weeks (Boston), and 18 months. There were also up to 3 check-in calls during weeks 1 through 3 to facilitate website use. The parent internet intervention component (5 modules) is based on an adaptation of the Preventive Intervention Project.^32^ A description of the intervention has been published.^18,33^

### HE Intervention

The HE intervention is an attention control internet site (14 modules) providing instruction on general health topics. The 14th module discusses mood and mental health treatment, and also addresses mental disorder stigma.^14,34^ Up to 3 check-in calls (weeks 1-3) were offered to ensure website access. The caregiver internet program (4 modules) is similar.

### Intervention Shared Elements

Both interventions were consistent with guidelines for adolescent depression in primary care including the following: training clinicians in depression identification, diagnosis, and treatment; establishing referral relationships; screening; using a formal tool to determine depression risk; assessing depression; interviewing caregivers and adolescents; educating caregivers and adolescents on treatment; establishing treatment plans; and establishing safety plans.^35^ These steps are closely related to the Chronic Care Model.^36^ Rates of episodes were extremely low for this high-risk sample. When episodes were identified, adolescents were referred for treatment, and caregivers and pediatricians were notified.

### Instruments

Instruments have been described previously.^18^ The 2-question screener was based on the Patient Health Questionnaire for adolescents.^37,38^ The K-SADS^39,40^ is a semistructured interview assessing current and lifetime psychiatric diagnoses in participants aged younger than 18 years, administered to parents and adolescents.^39,41^ The DSRs are obtained from the Kiddie Longitudinal Interval Follow-up Evaluation^41^ for each week of the follow-up interval, and GAS ratings were assigned at each assessment. For both the K-SADS and the Kiddie Longitudinal Interval Follow-up Evaluation, precipitating events were reviewed, and episodes secondary to medical concerns were indicated, if they occurred. The CES-D_10_ measures the frequency of 10 depressive symptoms over the past week, using a 4-point scale; it was completed at baseline, 2, and 6 months.^22^ Demographic information was collected at baseline, including race and ethnicity, using categories defined by the study team. Fidelity and exposure to the intervention were based on module completion, and completion and rating of the MIs, with 2 trained raters using the MI Treatment Integrity coding manual (version 4.2.1),^42^ and number of characters typed into the CATCH-IT website.

### Sample Size

We required 200 participants per intervention condition to achieve 80% power based on a conservative application of our pilot study findings.^40^ These calculations assumed that in the control group 72% are free from depression after 1-year follow-up, and the second year continues to follow the same exponential rate for controls; for intervention, the hazard is a constant ratio of 0.62, and an attrition rate of 7% for each of the first 4 quarters, and 2% for each of the second 4 quarters (Trial Protocol in Supplement 1).

### Statistical Analysis

The trial tested for differences between group medians in website engagement using Wilcoxon rank sum tests. We estimated incidence rates by calculating the number of depressive episodes per 10 000 person-weeks of follow-up. Kaplan-Meier curves were used to estimate the time to first episode distribution for each intervention under 6 different treatment allocations (eTable 1 in Supplement 2). Treatment allocations were developed based on existent literature with regard to threshold effects of adherence as an a priori analytic strategy. The thresholds were applied in the same manner with both interventions with similar numbers of persons identified in each arm. Cox proportional hazard regression was used to estimate the HR comparing CATCH-IT with HE. We present adjusted (sex, ethnicity [Hispanic/nonHispanic], race [white/nonwhite], baseline age, site, and baseline CES-D_10_ score) and unadjusted HRs. The assumption of proportional hazards was checked by testing the independence between the Schoenfeld residuals and time.^43^ The trial examined moderating effects of baseline adolescent CES-D_10_ score as a continuous variable, exhibited across a range of possible CES-D_10_ values, by including interaction terms in the Cox models. We used linear mixed-effect growth models with random intercepts and slopes to examine differences between group change over time in CES-D_10_ and GAS. Analyses were adjusted for the covariates listed above. We used propensity scores to account for differences between treatment groups in the per protocol analysis (≥2 modules completed) that could otherwise confound treatment effect estimates controlling for: site, age, sex, ethnicity, race, mother’s education, parents’ marital status, number of siblings, firstborn child, times moved, current GAS score, most severe past GAS score, highest past GAS score, and baseline CES-D_10_. Analyses were conducted using R statistical software, version 3.3.1 (R Foundation Inc), SAS, version 9.4 (SAS Institute), and Mplus, version 8 (Muthén & Muthén).

### Missing Data

The percentages of participants missing each K-SADS or CES-D_10_ assessment were calculated. We used a logistic regression model to determine whether those missing from follow-up differed from those who were not. We also used multiple imputation to assess the potential for differential follow-up by intervention condition. We constructed 50 data sets for each site and intervention condition with fully saturated specification by condition interacting with the following variables: all CES-D_10_ and GAS values (0, 2, and 6 months), screening CES-D_10_, baseline age, sex, ethnicity, race, and maternal education. These were combined into 50 complete imputed data sets and analyzed separately using the growth models described above; results were pooled.

## Results

### Implementation

**Figure 1.**
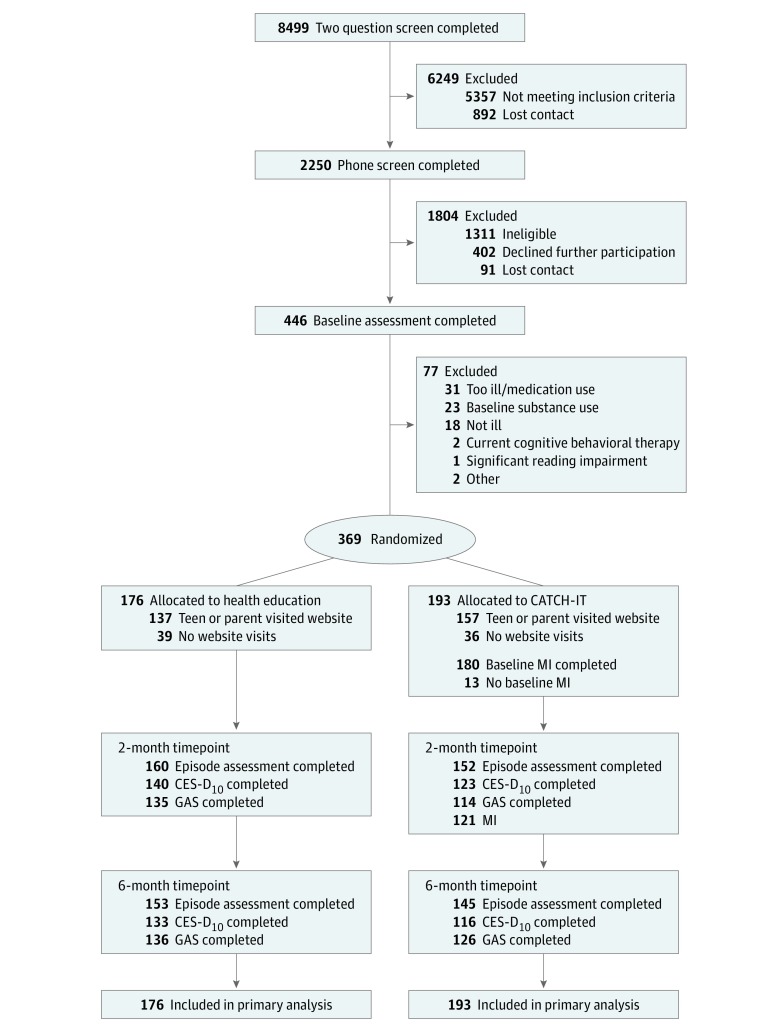
Consort Diagram CATCH-IT indicates competent adulthood transition with cognitive behavioral humanistic and interpersonal training; CES-D_10_, Center for Epidemiologic Studies Depression scale; GAS, Global Assessment Scale; MI, motivational interview.

We implemented the study in 8 health systems from 31 practices in a defined population of more than 41 000 adolescents. There were 8499 adolescents screened, 2250 phone assessments, 446 enrolled, and 369 randomized. The 2 groups consisted of CATCH-IT (n = 193) and HE (n = 176) (Figure 1). Among these participants, 28% had both a past episode and subsyndromal depression; 12% had a past episode only, 59% had subsyndromal depression only, and 1% had borderline subsyndromal depression

### Sample

Participants were aged 13 to 18 years (mean [SD] age, 15.4 [1.5] years; 251 women [68%]) with history of depression and/or current subsyndromal depressive symptoms. Participants were diverse in self-reported race and ethnicity: 21% Hispanic, 26% non-Hispanic black, 43% non-Hispanic white, 4% Asian, and 6% multiracial or other. Sixty-one percent had married parents, and 53% of the fathers were college graduates. Adolescents were moderately depressed (CES-D_10_: mean [SD], 9.4 [4.6]), moderately impaired (GAS: mean [SD], 78.1 [9.4%]), had a prior DSR of 3 or more (226 [62%]), and had a prior DSR of 4 or more (144 [40%]) (Table 1).

**Table 1.** Participant Characteristics at Baseline

Characteristic	All (N = 369)		CATCH-IT (n = 193)		Health Education (n = 176)	
	No.	No. (%)	No.	No. (%)	No.	No. (%)
Age, mean (SD), y	369	15.4 (1.5)	193	15.4 (1.5)	176	15.5 (1.5)
Sex	369		193		176	
Male		118 (32)		59 (31)		59 (34)
Female		251 (68)		134 (69)		117 (66)
Ethnicity	369		193		176	
Hispanic		77 (21)		41 (21)		36 (20)
Non-Hispanic^a^		292 (79)		152 (79)		140 (80)
Race	369		193		176	
White		201 (54)		107 (55)		94 (53)
Nonwhite^b^		168 (46)		86 (45)		82 (47)
Mother’s education	359		188		171	
Some high school		12 (3)		5 (3)		7 (4)
High school graduate/GED		45 (13)		20 (11)		25 (15)
Some college		87 (24)		44 (23)		43 (25)
College graduate		215 (60)		119 (63)		96 (56)
Father’s education	336		177		159	
Some high school		26 (8)		12 (7)		14 (9)
High school graduate/GED		76 (23)		36 (20)		40 (25)
Some college		55 (16)		37 (21)		18 (11)
College graduate		179 (53)		92 (52)		87 (55)
K-SADS						
GAS, mean (SD)^c^						
Current	367	78.1 (9.4)	193	78.3 (9.3)	174	78.0 (9.6)
Most severe past	359	67.5 (10.9)	189	68.1 (10.3)	170	67.0 (11.5)
Highest past	360	82.2 (8.5)	190	82.3 (8.4)	170	82.1 (8.5)
DSR, mean (SD)^d^						
Most severe	364	3.1 (1.4)	189	3.1 (1.4)	175	3.2 (1.4)
≥3		226 (62)		113 (60)		113 (65)
≥4		144 (40)		75 (40)		69 (39)
Current	365	1.8 (0.9)	190	1.7 (0.9)	175	1.8 (0.9)
CES-D_20_, mean (SD)^e^	362	16.9 (8.7)	190	17.3 (8.7)	172	16.5 (8.8)
CES-D_10_, mean (SD)^f^	362	9.4 (4.6)	190	9.5 (4.5)	172	9.4 (4.6)
SCARED total score, mean (SD)^g^	312	25.3 (12.3)	171	25.5 (12.7)	141	25.2 (11.9)

Abbreviations: CATCH-IT, Competent Adulthood Transition with Cognitive Behavioral Humanistic and Interpersonal Training; CES-D, Center for Epidemiologic Studies Depression scale; DSR, Depression Severity Rating; GAS, Global Assessment Scale; GED, general equivalency diploma; K-SADS, Kiddie Schedule for Affective Disorders scale; SCARED, screen for child and anxiety related disorders.

^a^ Participants with missing ethnicity data were coded as non-Hispanic (n = 6).

^b^ Participants with missing race data were coded as nonwhite (n = 20; most identified as Hispanic).

^c^ Possible range: 1 to 100; a higher score indicates higher functioning.

^d^ Possible range: 1 to 6; a higher score indicates more severe depression.

^e^ Possible range: 0 to 60; a higher score indicates more severe depression.

^f^ Possible range: 0 to 30; a higher score indicates more severe depression.

^g^ Possible range: 0 to 82; a higher score indicates greater anxiety.

### Fidelity and Intervention Exposure

Intervention use was monitored and recorded. The number of MIs completed was recorded for CATCH-IT participants. Table 2 shows that CATCH-IT adolescents and parents spent more time using the intervention, but CATCH-IT adolescents completed fewer modules than HE adolescents (modules completed: median [interquartile range], 1.0 [4.0] vs 4.0 [14.0], respectively; *P* = .003). Both study arms received a sizable dose of the interventions, with the combined (parent + adolescent) module completion greater for HE (modules completed: median [interquartile range], 4.0 [8.0] vs 8.0 [17.0], respectively; *P* < .001). Adolescents and parents included in CATCH-IT typed a mean (SD) of 3071 (4572) and 716 (977) characters, respectively (Table 2). Over 73% of MIs and phone calls were completed. Mean (SD) interview length was 7.7 (4.0) minutes, mean (SD) technical global rating was 3.0 (0.5) on a 1 to 5 scale, and mean (SD) relational global rating was 2.9 (0.6) (eTable 2 in Supplement 2).

**Table 2.** Fidelity Assessment of Internet Component

Website Use	Mean (SD)		Difference CATCH-IT and Health Education, Mean (95% CI)	Median (IQR)		*P* Value^a^
	CATCH-IT	Health Education		CATCH-IT	Health Education	
Adolescents						
No.	193	176				
Modules completed, No.	3.4 (4.7)	6.8 (6.5)	−3.4 (−4.5 to −2.2)	1.0 (4.0)	4.0 (14.0)	.003
Total time on site, min	100.2 (143.1)	22.8 (31.0)	77.4 (55.7 to 99.0)	39.6 (149.2)	8.4 (35.1)	<.001
Days visited site	3.7 (4.5)	1.4 (1.6)	2.3 (1.6 to 3.0)	2.0 (4.0)	1.0 (2.0)	<.001
Total characters typed, No.	3071 (4572)	NA	NA	923 (4469)	NA	NA
Adolescents and parents						
Modules completed, No.	5.3 (5.8)	8.8 (7.3)	−3.5 (−4.8 to −2.1)	4.0 (8.0)	8.0 (17.0)	<.001
Total time on site, min	130.6 (157.9)	30.6 (35.6)	100.0 (76.1 to 124.0)	75.8 (192.2)	18.9 (40.8)	<.001
Days visited site	5.2 (5.2)	2.2 (2.2)	2.9 (2.1 to 3.8)	4.0 (6.0)	2.0 (2.0)	<.001
Total characters typed, No.	3713 (4932)	NA	NA	1899 (5792)	NA	NA
Parents						
No.	165	157				
Modules completed, No.	2.1 (2.0)	2.2 (1.9)	−0.1 (−0.6 to 0.3)	2.0 (4.0)	4.0 (4.0)	.80
Total time on site, min	32.6 (37.3)	8.6 (10.0)	24.0 (17.9 to 30.0)	22.4 (51.9)	5.6 (14.9)	<.001
Days visited site	1.6 (1.6)	0.9 (1.1)	0.6 (0.3 to 0.9)	1.0 (2.0)	1.0 (1.0)	<.001
Total characters typed, No.	716 (977)	NA	NA	101 (1205)	NA	NA

Abbrevations: CATCH-IT, Competent Adulthood Transition with Cognitive Behavioral Humanistic and Interpersonal Training; IQR, interquartile range; NA, not available.

^a^ Medians compared using Wilcoxon rank-sum test.

### Outcomes

**Figure 2.**
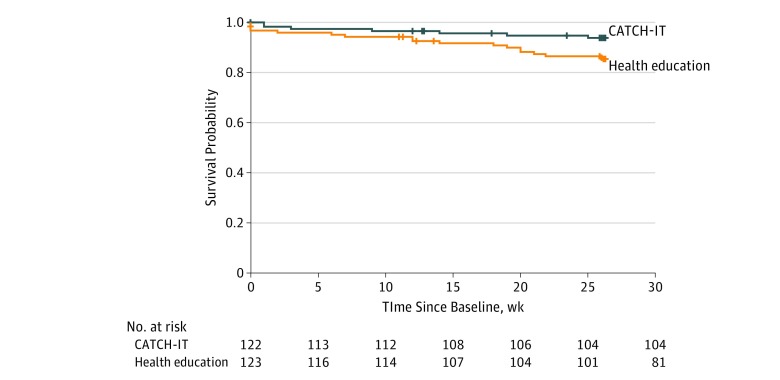
Time to First Depressive Episode for Those Completing 2 or More Modules (Per-Protocol 2 Analysis) CATCH-IT indicates competent adulthood transition with cognitive behavioral humanistic and interpersonal training.

**Figure 3.**
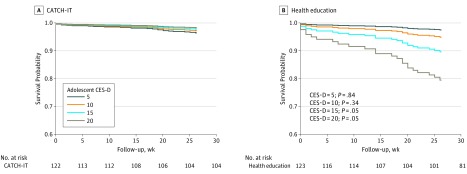
First Depressive Episode Survival Analysis by Adolescent Baseline CES-D Scores CATCH-IT indicates competent adulthood transition with cognitive behavioral humanistic and interpersonal training; and CES-D, Center for Epidemiologic Studies Depression scale.

For the primary outcome of time-to-depressive episode (DSR ≥3) using intention-to-treat analyses (N = 369), unadjusted HR was 0.59 (95% CI, 0.27-1.29; *P* = .18), and adjusted HR was 0.53 (95% CI 0.23-1.23, *P* = .14). Proportional hazards assumption was met (*P* = .89). For per protocol analysis (≥2 modules completed on either arm, n = 245) (Figure 2), unadjusted HR was 0.41 (95% CI, 0.17-0.99; *P* = .05), and adjusted HR was 0.44 (95% CI, 0.18-1.08; *P* = .07). After adjusting for potential confounders using propensity scores, HR was 0.52 (95% CI, 0.19, 1.42; *P* = .20). Additional analyses are shown in eTable 3 in Supplement 2, and incidence rates in eTable 4 in Supplement 2. The trial calculated the number needed to treat to indicate the number of adolescents who would need to receive the intervention to prevent 1 additional onset of depressive disorder and found a number needed to treat of 36 for the main effect of CATCH-IT. Adolescents with higher baseline CES-D_10_ scores showed a significantly stronger effect of CATCH-IT on time to event relative to those with lower baseline scores (HR, 0.82; 95% CI, 0.67-0.99; *P* = .04) (Figure 3; eTable 5 in Supplement 2). For example, the hazard ratio for a CES-D_10_ score of 15 was 0.20 (95% CI, 0.05-0.77), compared with a hazard ratio of 1.44 (95% CI, 0.41-5.03) for a CES-D_10_ score of 5. Sex, ethnicity, race, and age did not predict outcome or interact significantly with the interventions and outcomes. Both CATCH-IT and HE demonstrated reduced depressed mood and improved functional status, with no statistically significant differences at 6 months (eTables 6 and 7 in Supplement 2).

### Missing Data

At least 1 follow-up K-SADS was completed for 85% of the sample, at least 1 follow-up CES-D_10_ was completed for 80% of the sample, and at least 1 follow-up GAS assessment was completed for 81% of the sample. Dropout between CATCH-IT and HE was different at 2 and 6 months for both K-SADS and CES-D_10_ (eTable 8A in Supplement 2)_._ At 6 months, K-SADS data were missing for 48 participants (25%) of CATCH-IT and 23 participants (13%) of HE (*P* = .004), and CES-D_10_ was missing for 77 participants (40%) of CATCH-IT and 43 participants (24%) of HE (*P* = .002). Significant predictors of missing K-SADS at 6 months were randomization to CATCH-IT (CATCH-IT vs HE: odds ratio [OR], 2.62; 95% CI, 1.43-4.79; *P* = .002), living in Chicago (Boston vs Chicago: OR, 0.20; 95% CI, 0.09-0.46; *P* < .001), age at baseline (OR, 1.23; 95% CI, 1.02-1.49; *P* = .03), and maternal education (high school graduate or less vs college graduate: OR, 2.99; 95% CI, 1.37-6.53; *P* = .01) (eTable 8C in Supplement 2). Having a past episode or high CES-D_10_ at baseline was not associated with missing follow-up.

## Discussion

Overall, we observed a nonsignificant decrease in depressive disorders at 6 months in CATCH-IT as compared with HE. Adolescents and parents devoted substantial time to both interventions, and both conditions experienced decreased depressive symptoms and improved functional status. However, higher-risk adolescents demonstrated greater benefit from CATCH-IT, achieving as much as 80% risk reduction with a CES-D_10_ score of more than 15, but those without symptoms showed no such benefit. While regression to the mean is a possible explanation for the moderating effect of high CES-D on CATCH-IT, other studies have found that preventive effects for depression interventions are stronger for indicated vs universal samples.^44^ Moreover, the same effect did not emerge for the HE condition—higher CES-D scores did not moderate the effect of the HE condition—suggesting regression to the mean may not explain the group difference found. For the 66% of adolescent and parent pairs who completed at least 2 modules (63% for CATCH-IT and 70% for HE), the unadjusted analysis showed CATCH-IT reduced the risk of mDE and MDE by 59%, but this was not significant after adjustment for demographic factors or after analyses incorporating propensity scoring.

To our knowledge, this is the first clinical trial in adolescents to evaluate whether depressive episodes can be prevented in primary care settings.^11^ Our finding that the risk of depressive episodes may be reduced for adolescents with subsyndromal depression is consistent with our earlier phase 2 clinical trial, which only included adolescents with subsyndromal depression.^45^ Results were not significant in the intention-to-treat main effect analysis, but this may be the result of heterogeneity of treatment effect whereby CATCH-IT is favored for those with subsyndromal depression, but not for those with prior depressive episode alone. Perhaps CATCH-IT bored or frustrated adolescents without current symptoms, or conversely, elicited increased surveillance of symptoms or stimulated memories of prior episodes.^46,47^ Alternatively, adolescents who are not symptomatic may be less motivated to complete CATCH-IT, the more self-directed intervention, and may actually prefer HE, which did not require substantial effort, perhaps even gaining a sense of self-efficacy.^34,46,47,48,49^ Also, despite spending substantial time engaged with this intervention, the low number of modules completed may have attenuated impact. Additionally, the borderline significant findings favoring CATCH-IT with the completion of 2 modules (eTable 3 in Supplement 2) suggest that there may also be a threshold effect whereby sufficient numbers of modules may need to be completed for CATCH-IT to be more efficacious than HE.

Depression prevention programs have shown mixed results.^12^ The only other primary care trial with adolescents demonstrated improvements in explanatory style but not depressed mood.^50^ Our findings showing that increased participation may predict better outcomes are consistent with prior reports.^50,51^ The observed risk reduction across multiple outcomes (DSR ≥3, ≥4, and 5; eTable 3 in Supplement 2), even if not statistically significant, is comparable with other trials.^7,8,28^ While most internet interventions demonstrate favorable changes in depressed mood, this study did not demonstrate between group differences for mood or functional status.^10^ However, this is similar to the phase 2 clinical trial of CATCH-IT, which demonstrated lower cumulative prevalence of depressive episodes, but not between group differences in depressed mood.^38^ It is possible the extensive human contact within this trial had an ameliorating effect on mood and strengthened functional status, effectively blurring between group results. A clinical trial of CATCH-IT in Hong Kong that only used self-report instruments demonstrated a significant between group effect (effect size = 0.36) for depressed mood at 12 months.^17^

This study has a robust prevention design implemented in a population-based model in primary care.^52,53^ The implementation of our study at 2 sites and 8 health systems has rarely been accomplished in studies of child psychiatric conditions.^20^ This study fits the model by Curran et al^19^ of hybrid efficacy and implementation studies and substantially enhances generalizability. The attention control condition, which included guidelines for adolescent depression in primary care and chronic care model elements, no doubt reduced between-group differences.^24,36^ However, given the need for ethical care of adolescents at risk for depressive episodes, a “no intervention” or “wait list control” condition is not possible.^53,54^

### Limitations

This study had limitations, including the relatively low adherence rate of teens and parents. Module completion for CATCH-IT was consistent with pilot findings. A review of internet-based mental health interventions for youths revealed completion ranged from 24% to 85%, and it was not necessary to complete the entire intervention for positive benefits to emerge.^12^ In addition, module completion does not correlate with time spent, as the HE modules are significantly shorter than CATCH-IT; overall, CATCH-IT participants spent more time using the intervention. Nevertheless, future research should examine why adolescents did not complete the interventions, and explore strategies for boosting adherence. Also, our incidence rate for depressive disorders was low, thus, increasing the number of participants needed to have adequate power to detect group differences. We do not know for certain whether intervention effects can be attributed to the internet-based modules or to the MIs, although results from our pilot study suggest that adolescents who did not get MIs still evidenced reduced symptoms of depression at follow-up. Other limitations include the findings of differential attrition, which were adjusted analytically, and the fact that researchers enrolled only 92% of the target sample.

## Conclusions

Our long-term goal for the CATCH-IT intervention is to provide a first-line program for primary care physicians to offer as part of the guidelines for adolescent depression in primary care, to support adolescents while the need for further intervention can be evaluated. We continue to examine moderators that may explain who responds best to this approach. Future directions include the development of versions for personal devices (eg, tablets and mobile phones), and a version individualized for sexual- and gender-minority teens.

A scalable, population-based approach to preventing depression in adolescents in primary care may be efficacious for adolescents with subsyndromal depression, but not for those with a prior episode alone.

## Notes

Supplement 1. **Trial Protocol**

Supplement 2. **eTable 1.** Definition for Criteria for Main Outcomes Analyses **eTable 2.** MI Fidelity: Mean MITI Summary Scores **eTable 3.** Hazard Ratio Estimate and 95% CI for First Depressive Episode Comparing CATCH-IT to Health Education **eTable 4.** Incidence of First Depressive Episode by Treatment Group **eTable 5.** Adjusted Cox Proportional Hazard Model Results, Including Adolescent Baseline CES-D_10_ as a Moderator **eTable 6.** Depressed Mood **eTable 7.** Functional Status **eTable 8.** A, Participants With Missing Data: KSADS and CES-D_10_. B, Predictors of Missing 2-Month Follow-up: Time to Last Assessment <2 Months. C, Predictors of Missing 6-Month Follow-up: Time to Last Assessment <6 Months. D, Predictors of Missing 2-Month CES-D_10_. E, Predictors of Missing 6-Month CES-D_10_

Supplement 3. **Data Sharing Statement**
