# Supplementary material for: Effect of Internet-Based Cognitive Behavioral Humanistic and Interpersonal Training vs Internet-Based General Health Education on Adolescent Depression in Primary Care: A Randomized Clinical Trial
Source: JAMA Netw Open. 2018 Nov 2;1(7):e184278. doi: 10.1001/jamanetworkopen.2018.4278 (PMC6286074; doi:10.1001/jamanetworkopen.2018.4278)
Supplement: Supplement 1. — Trial Protocol [file jamanetwopen-1-e184278-s001.pdf]

1. Title Page

Researcher/Principal Investigator: Benjamin Van Voorhees

Start date of project: July 15, 2011

Title of Project: Promoting AdolescenT Health (PATH)

Investigators and Affiliations

Principle Investigator:
Benjamin W. Van Voorhees, MD, MPH
Visiting Associate Professor of Pediatrics
Chief, Section of General Pediatrics and Adolescent Medicine

Co-Investigator
Carl Bell, MD
Professor of Clinical Psychiatry
Director, Institute for Juvenile Research

Co-Investigator
Dr. Florence Desrosiers Assistant Professor Clinical Pediatrics Adolescent Medicine

Chicago Local performance sites outside of UIC

Mickey Eder, Ph.D.
ACCESS Health care

Jason Canel, MD
Northshore university health systems

Karen Walker, MD
The Children's Clinic

Anita Berry, MSN, CNP, APN, PMHS
Advocate Children's Hospital

James Cantorna, MD
Franciscan St. Margaret Health

Other sites

Tracy Gladstone, Ph.D. PI
Wellesley College (2nd performance site)

Boston local performance site

Eumene Cheng, MD
Harvard Vanguard Health Systems

Other sites continued

Hendricks Brown, Ph.D. Co-I
Northwestern University (analytic site)

Consultants

Mark Reinecke, Ph.D.
Consultant
Northwestern University

William Beardslee, MD
Consultant
Harvard Medical School

Robin Weersing
Consultant
University of California, San Diego

Sponsor: National Institutes of Mental Health 1 RO1 MH090035-01A1 Primary Care Internet Based Depression Prevention for Adolescents (CATCH-IT 3)

Protocol version: 16

Executive Summary and Organizational Overview:

Developing new interventions that incorporate the "diverse needs and circumstances of people with mental illness," particularly in primary care and community settings, is a key NIMH strategic objective. Prevention of mental disorders has become a priority for the NIMH, which emphasizes the importance of developing "new and better interventions" to "…preempt the occurrence of disease." These interventions must (1) work in multiple and diverse settings (e.g. primary care); (2) be suitable for delivery outside of traditional mental health systems (3) use new technologies; (4) build on previous clinical trials; (5) reduce identified disorders/enhance functional outcomes; (6) include families and (7) be tailored to the individual. Despite these NIMH guidelines, while primary care physicians remain the first line providers for at-risk adolescents, there is no widely available, low cost and culturally acceptable preventive approach that targets depression in primary care settings. To address this gap and specified NIMH priority, Dr. Van Voorhees developed and conducted a phase 2 clinical trial of a primary care Internet-based depression prevention intervention (CATCH-IT, Competent Adulthood Transition with Cognitive Behavioral Humanistic and Interpersonal Training). In this study, the high intensity arm (i.e., motivational interview + internet site) demonstrated significant reductions in depressed mood and increases in protective factors (social support, motivation) and lower incidence of depressive episodes over 12 months (7% versus 28%), compared to the low intensity arm (internet site referral + only physician brief advice). We now propose the next step study, a phase 3 efficacy study. In this 5-year, two-site randomized clinical trial, we propose to test the efficacy of the CATCH-IT primary care/Internet based depression prevention intervention against Attention Monitoring Psychoeducation (HEALTH EDUCATION) in preventing the onset of depressive episodes in an intermediate to high risk group of adolescents aged 13-18. We plan to (a) identify high risk adolescents based on elevated scores on the PHQ-A, a screening measure of depressive symptoms; (b) recruit and randomize 400 (200 per site) of these at-risk adolescents into either the CATCH-IT or the HEALTH EDUCATION group; (c) assess outcomes at 2, 6, 12, 18, and 24 months post intake on measures of depressive

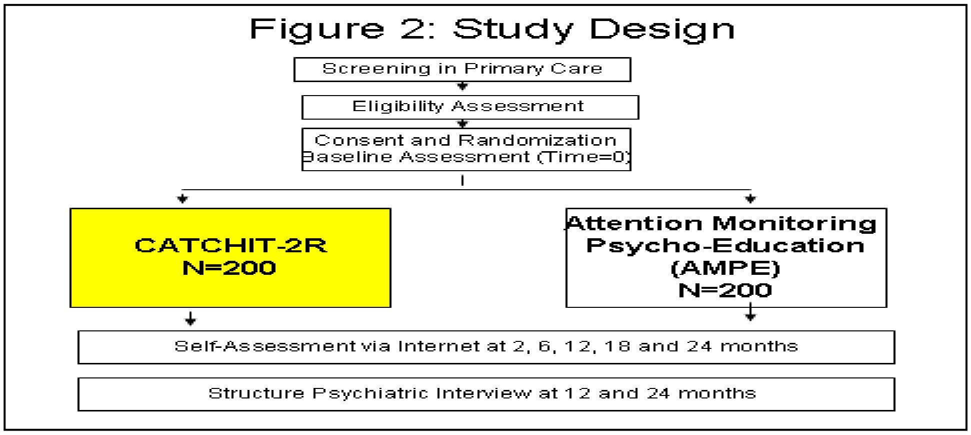

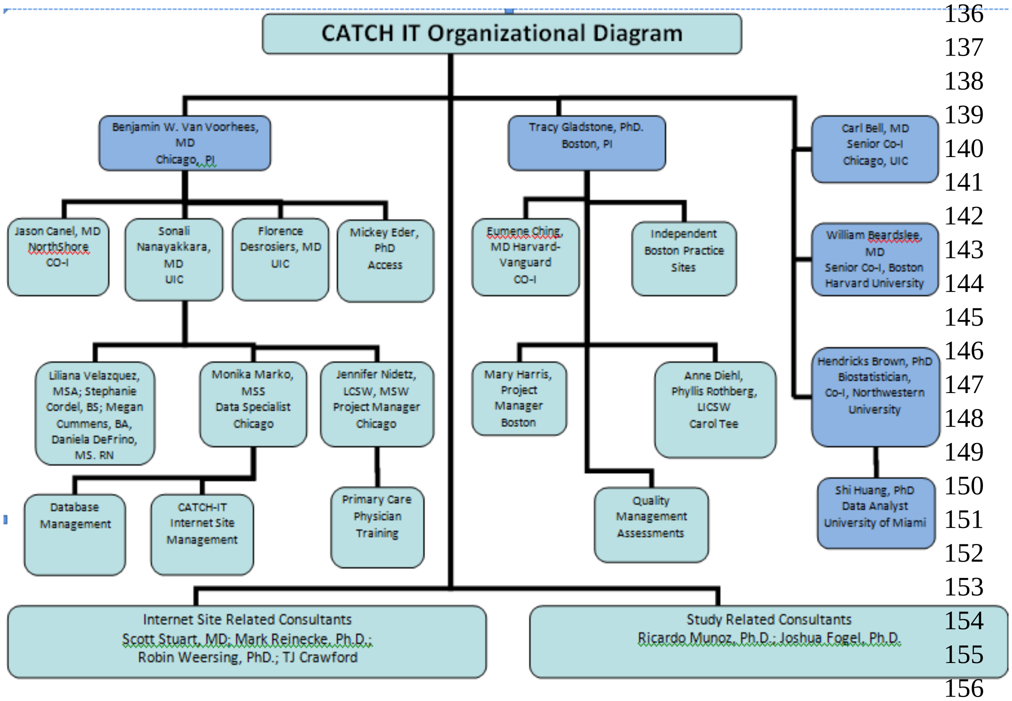

symptoms, depressive diagnoses, other mental disorders, and on measures of role impairment in education, quality of life, attainment of educational milestones, and family functioning; and (d) conduct **exploratory** analyses to examine the effectiveness of this intervention program, moderators of protection, and potential ethnic and cultural differences in intervention response.

2. Study Hypothesis and Specific Aims (purpose, objectives)

Significance: Prevention of mental disorders has become a key priority for the NIMH[1], but we have no widely available public health strategy to reduce morbidity and mortality. [2-10] We developed a primary care/Internet based depression prevention intervention strategy intended to meet the NIMH call for developing "new and better interventions" for "diverse needs and circumstances" to "…preempt the occurrence of disease." These interventions must (1) have broad "reach" into at-risk populations[11-13]; (2) work outside of traditional mental health systems; (3) use new technologies; (4) build on previous clinical trials; (5) reduce identified disorders/enhance functional outcomes; (6) include families; and (7) be personalized.[1] Primary care for adolescents provides a nearly untapped new opportunity to build cost-effective prevention strategies for depression[13]. **Innovation**: Based on face-to-face manual-based interventions[14-14-18] of demonstrated efficacy[15, 19-21], the CATCH-IT intervention (Competent Adulthood Transition with Cognitive Behavioral Humanistic and Interpersonal Training (CATCH-IT) [19, 22-24] represents a paradigm shift. Key innovations include: (1) it is the first of its kind, a public health adolescent depression prevention strategy (low cost, easily disseminated, acceptable and feasible primary care/Internet model);[25-26] (2) it combines a brief primary care-based motivational program with an Internet-based self-directed Cognitive Behavioral (CBT) and Interpersonal Psychotherapy (IPT) approach to address the key barrier of engagement/adherence; (3) it targets both adolescent and parent vulnerability and protective factors in separate interventions, using an ecological model; (4) it is personalized to the presence of parental depression and ethnicity/culture, key moderators in prior studies; and (5) it uses media based learning strategies including music, videos, and stories to convey learning. [19-20, 27-28] We demonstrated feasibility and potential efficacy in a randomized clinical trial in 12 primary care practices in 4 states/5 health systems (40% ethnic minorities) supported by a K-08 Award (2006), a NARSAD foundation Young Investigator Award (2004), and a RWJ Foundation Depression in Primary Care value grant (2005). In this study, CATCH-IT 3 demonstrated significant reductions in depressed mood (vulnerability), increased social support (protection), and reduced depressive episodes at 12 month follow-up (6.98 versus 27.50, p-value =0.013). [29-30] **Approach**: In this application, we propose the next step study, a 5-year, two-site (University of Illinois at Chicago/Access Healthcare and Wellesley College/Harvard Vanguard) randomized clinical trial (analytic site is Northwestern University), to test the efficacy of a second generation CATCH-IT 3 (revised to include engaging media based learning) prevention intervention against Attention Monitoring and Psycho-education (HEALTH EDUCATION) in preventing the onset of depressive episodes in an intermediate to high risk, geographically representative sample of adolescents aged 13-18. We plan to: (a) identify high risk adolescents based on elevated depressed mood scores (cut-off set to maximize sensitivity and specificity for future episode)[31-33]; (b) recruit 400 (200 per site) of these at-risk adolescents to be randomized into either the CATCH-IT 3 or the HEALTH EDUCATION group; (c) assess at baseline and at 2, 6, 12, 18 and 24 months post intake on measures of depressive symptoms, depressive diagnoses, other mental disorders, and on measures of role impairment in education, quality of life, attainment of educational milestones, and family functioning; and (d) examine predictors of intervention response, and potential ethnic and cultural differences in intervention response.

Aim 1: To determine whether the CATCH-IT 3 depression prevention intervention prevents or delays major depressive episodes, as well as non-affective disorder episodes, compared to HEALTH EDUCATION. **Hypothesis 1**: Compared to youth in the HEALTH EDUCATION condition, youth assigned to CATCH-IT 3 will have a lower hazard ratio of major depressive episodes and non-affective disorder episodes over 2 years.

Aim 2: To determine if participants in the CATCH-IT 3 group exhibit more rapid favorable changes of depressive symptoms/and or vulnerability/protective factors compared with the HEALTH EDUCATION group. **Hypothesis 2**: Compared to youth in the HEALTH EDUCATION condition, youth in the CATCH-IT 3 program will demonstrate a steeper slope of improved symptoms through growth curve analysis and fewer depressed days[34] over two years.

<u>Aim 3:</u> To determine if participants in the CATCH-IT 3 program report lower perceived educational impairment, greater quality of life, greater health-related quality of life, and lower incidence of other mental disorders (anxiety, substance/alcohol use) as compared to participants in HEALTH EDUCATION. **<u>Hypothesis 3</u>**: Compared to youth in the HEALTH EDUCATION group, youth in the CATCH-IT 3 program will demonstrate more rapid benefits in reduced educational impairment, improved quality of life, and fewer disorders, as shown through steeper changes in slopes based on growth curve analysis over 2 years.
<u>Aim 4</u>: To determine for whom (moderators) and how (mediators) the CATCH-IT 3 program works in this population (Figure 1). **<u>Hypothesis 4A</u>**: CATCH-IT 3 effects will be moderated by six domains: (1) demographic/cultural factors, [35-37] (2) vulnerability factors/adverse events, [33] (3) motivation,[38] (4) physician relationship, (5) parent/child co-morbid psychopathology, and (6) treatment. [20, 39] **<u>Hypothesis 4B</u>**: The relation between CATCH-IT 3 participation and reduction in depressive episodes will be mediated by adherence to the Internet [40] and motivational interview fidelity as they alter vulnerability factors (e.g. motivation, cognition and social support) and responses to adverse events, which in turn impact the likelihood of episodes.[39, 41-43]

Exploratory Aim 1: To determine the implementation feasibility of the intervention from the physician/nurse practitioner and office nurse/medical assistant perspective as well to describe the practices in relationship to the medical home model.

Exploratory Aim 2: To determine whether CATCH-IT 3 has a favorable cost-benefit ratio and/or cost effectiveness of <$50,000/disability adjusted life year compared to the HEALTH EDUCATION group.

<u>3. Background and Significance</u>

a. Significance of Research Project

<u>We have no public health strategies to prevent depressive disorder in adolescents (importance):</u> The lifetime prevalence of major depressive disorder in adolescents aged 15-17 is 14%, and an estimated 20% of adolescents will experience depression by age 17,[44] with considerable long-term impairment and mortality. [31, 44-49] Although efficacious psychosocial and pharmacological treatments for adolescent depression have been demonstrated, [50-51] any single approach only helps about 50-75% of those treated. [23, 52-54]Furthermore, adolescents have low rates of care seeking, receiving high quality treatment (35% and 20%),[10, 55-56] and completing referrals for psychotherapy (30%).[57] Therefore, prevention of depression in high-risk individuals may be more cost-effective and less distressing than waiting for the condition to appear and then trying to treat a full depressive episode. We now have substantial evidence that cognitive behavioral approaches may prevent adolescent depression,[58-61]but we have no public health strategy to deliver these approaches to the general population.
<u>CATCH-IT provides an entirely new prevention strategy for mental disorders to address five key barriers in the field (improving scientific knowledge and medical practice):</u> To address (1) cost, difficulty in distribution and low acceptability of high fidelity interventions (face-to-face delivery), CATCH-IT [19, 22-24] delivers a high fidelity[14-14-18] intervention via the Internet and primary care in teen-friendly language.[25-26] For (2) lack of motivation and engagement[19-20, 27-28] , we use a social marketing[62] and motivational interviewing strategy.[25, 38] With respect to (3) lack of targeting epidemiologic context including family, CATCH-IT targets the multiple etiological elements acting either in concert or in combination, including negative cognitions[63-64], poorer social skills[31, 65 31, 66], stressful events, subsyndromal depressive symptoms[31, 33], and the absence of protective factors (e.g., high self-esteem, coping skills) by engaging both <u>parent and adolescent</u> with distinct behavior change programs. [31, 39, 41, 43, 67] In terms of lack of (4) cultural relevance and (5) multi-modal learning opportunities, CATCH-IT uses a multi-channel learning process and culturally relevant lessons, stories and music, based on face-to-face manual-based interventions[14-14-18] of demonstrated efficacy.[15, 19-21] <u>Should this intervention prove efficacious, the public would have the first efficacious, low-cost and acceptable depression prevention intervention that would be universally available without creating any new care delivery systems or further burdening the mental health care delivery system</u>.

<u>CATCH-IT creates impetus for use of technology-based prevention of mental disorders</u>: We create a first of its kind method for screening (i.e., adolescents with elevated symptoms of depression), identifying and preventing a major mental disorder in primary care. This approach targets youth at risk for a major mental disorder with what we call an emerging behavioral vaccine.[63, 68-69] Even partially effective, systematically distributed "behavioral vaccines" could alter illness trajectories and dramatically reduce morbidity and potentially even mortality (suicide). Evolving interventions encompassing true machine learning and integrating biomarker and genotyping data from the primary care electronic medical record with behavioral data, could construct endo-phenotypes and projected trajectories. From these risk prediction and trajectory models, primary care-Internet based interventions could seamlessly deploy Internet/cell phone, peer-to-peer, and professional-to-patient interventions, with the appropriate content, at the appropriate time, in the preferred format, and at the moment most likely to alter trajectories towards resiliency and away from vulnerability.

b. Innovation

<u>Challenging the theoretical orientations by combining cognitive-behavioral/interpersonal/family-based interventions in an ecological model (changing theory)[69]:</u> We combine theoretical models to target vulnerability factors identified in epidemiologic studies, rather than adhering to a psychotherapy model, as has been done in the past (Figure 1).[20, 31, 39, 41, 43, 67] Changes in negative cognitions have been found to mediate the relation between cognitive therapy and depression.[70] CATCH-IT 3 trains adolescents in cognitive-restructuring and interpersonal skills to permit them to reduce negative cognitions before they become too severe, and thereby avert the development of full depressive syndromes. In addition, substantial epidemiologic work, including that done by the principal investigators, suggests that adolescents' families are a substantial source of vulnerability and protection with regard to future incidence of depression.[33, 69, 71-72] Adverse family environments are among the most consistent risk factors for adolescent depression.[73] Adolescent depressive symptoms have been connected to the quality of adolescents' family relationships,[74] and family factors have been found to predict outcome and treatment response among depressed youth.[75-76] Given the benefits of family change in promoting resilience in adolescents,[77-79] it follows that an important aim for depression prevention programs is to help **change** the maladaptive aspects of the family environment.

<u>Generating new methods to test theories of prevention:</u> At this point, we do not know which elements of preventive strategies make them effective. We describe our conceptual model in Figure 1 (numbers apply to model). Here, (1) the primary care motivational interview is intended to enhance adherence/engagement with the Internet program, and (2) adolescent CBT/IPT and parent Internet components are expected to alter individual, family and peer/school related behaviors, (3) so as to reduce the incidence of disorder (4) and enhance developmental progress/functional status. In terms of moderators, we propose six domains: (1) demographic and cultural factors,[35-37] (2) vulnerability factors and adverse events,[33] (3) motivation and attitudes,[38] (4) physician relationship, (5) parent and child co-morbid lifetime psychopathology, and (6) treatment before and during the study.[20, 39] With regard to mediators, we propose that the relation between CATCH-IT 3 participation and reduction in depressive episodes will be mediated by adherence to the Internet program in specific areas of behavior change (e.g. CBT and IPT elements)[40] and motivational interview fidelity, as they alter vulnerability factors (e.g. cognition and social support) and responses to adverse events, which in turn impact the likelihood of episodes.[39, 41-43]<u>Internet based moment-to-moment measures of</u>

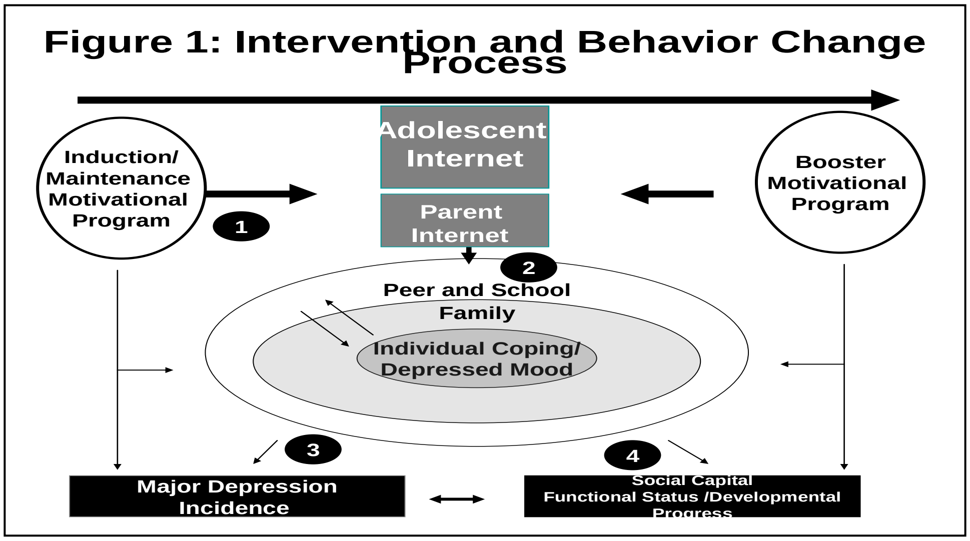

Figure 1: Intervention and Behavior Change Process

topic-specific learning adherence (time, page use, ratings, mouse clicks, and text responses) have never been available before to test hypothesized relations between psychotherapy intervention elements, vulnerability factor changes, and subsequent outcomes in an ecological context.

Exploiting new technologies by developing a new Internet delivery paradigm to address limited supply of face-to-face mental health resources: Primary care patients find group therapy to be the least acceptable treatment for depression, although this is currently the only approach of demonstrated efficacy.[20, 80]Low-intensity universal interventions (e.g. high school health classes) are more acceptable to adolescents but have shown variable results.[81] Long distances and the limited supply of mental health specialists in many regions may make group and face-to-face counseling difficult to implement. The Internet is a promising modality for the delivery of preventive interventions and offers advantages such as interactivity, personalization for adolescents and wide availability through libraries and schools, even for ethnic minority youth.[38, 82].[83].[84] [81, 84-89] Adolescents with obesity, smoking, alcohol use, HIV/AIDS risk, and sexual risk-taking have achieved website visit rates of 45% to 96% and favorable behavior change in studies of motivated volunteers. [38, 82, 90-92]. [93-96] However, use of health promotion Internet sites appears to be limited to only the highly motivated, particularly in mental health, [97-101] and systematic engagement may be essential for Internet based approaches to have a public health impact in adolescents. [102]

Innovating new models for engagement with low-intensity interventions: Although adolescents may be unlikely to complete free-standing Internet based interventions, [86-87] when combined with some minimal amount of face-to-face contact, self-directed interventions may have higher completion rates and greater reductions in symptoms (e.g. risk). There is substantial theoretical and empirical support for the use of motivational interviewing to enhance behavior change in adults and adolescents. The hypothesized mechanism of action is increased quantity and quality of motivation by strengthening the internal rationale around innate needs for competence, autonomy and connection.[103-105] We know of no preventive approach for depression that seeks to enhance motivation. The value of motivational interviewing has now been demonstrated in multiple studies with moderate to large effect sizes.[106-110] Based on the Trans-theoretical Model of Change,[105, 111] the CATCH-IT 3 intervention uses motivational interviewing, goals setting, and telephone coaching to enhance the quality and quantity of motivation to prevent depression (e.g. adhere to intervention and practice copings skills, Figure 1). [112]

Creating a new primary care mental disorder prevention model: The American Psychological Association has recommended the development and implementation of preventive interventions in medical settings because of their focus on health and privacy, [113] but to date there are few such programs.[13] Most adolescents see their primary care physician at least once a year and are receptive to counseling and advice from their physician about their health and behavior.[114] Primary care physicians have been successfully trained to use and apply motivational interviewing techniques and are interested in applying new approaches to improve outcomes of psychosocial disorders.[115-118] A primary care-based intervention may be more acceptable than specialty mental health services to adolescents and would provide the primary care-based psycho-social guidance many seek, [57, 80, 114] particularly for ethnic minority youth.[37]

c. Preliminary Studies

The Preventive Intervention Research Cycle*:* The preliminary studies of the investigators involved in the proposed prevention project are described using the framework for conducting empirical prevention research outlined by the Institute of Medicine (IOM).[119]

Step 1: Identify the problem or disorder and review information to determine its extent. The proposed prevention effort focuses on adolescent depression, particularly as it presents in the primary care setting. **Van Voorhees, Gladstone and Bell** studied the epidemiology of depressive disorders in community[33, 36, 69, 71-72] and primary care settings and found that 21% (N=293) of those screened (estimated > 1,200) in primary care reported a core symptom of depressive disorder and were at elevated risk.[29] **Gladstone** and **Beardslee** have also reviewed the literature on preventing youth depression.[120-122]

Step 2: Review relevant information about risk and protective factors from both prevention and non-prevention studies. Regarding risk and protective factors for adolescent depression, **Van Voorhees, Gladstone, Bell,** and **Reinecke** conducted a series of analyses to identify factors predicting depressive

episodes in adolescents.[71] Family “connectedness,” parental warmth, active coping, peer acceptance, better school performance and religious activities were protective, and poor affect regulation and greater depressed mood predicted higher risk. [33, 36, 69, 71-72] [69] **Gladstone** and Kaslow[123] showed that there is a significant connection between cognitive styles and depressive symptoms in youth, and that cognitive styles interact with gender to predict depressive symptoms.[124] **Brown** has extensively studied developmental trajectories in youth. <u>Knowledge developed from these risk studies forms the foundation of the CATCH-IT intervention</u>

<u>**Step 3: Design, conduct, and analyze pilot studies and replication trials of the preventive intervention.**</u>

Step 3 is the critical link between theory and intervention. We propose an efficacy study based on steps 1 and 2 of the IOM research cycle, and based on the preliminary studies described below. In an efficacy study of an intervention targeting depressed youth, the *Preventive Intervention Project (PIP),* investigators **Beardslee** and **Gladstone** found that, 4.5 years after enrollment, children evidenced lower levels of internalizing symptoms, positive changes in child-related behaviors and also reported better family functioning and increased understanding of parental depression. [125] The PIP intervention has been adapted into and forms the core of the CATCH-IT 3 parent intervention. **Van Voorhees** K-08 research forms the foundation of this application and this preliminary work is described below.

A. Development of the CATCH-IT Intervention and Model: Interdisciplinary team of investigators in the development of a prototype intervention, including an initial motivational interview (MI) in primary care to engage the adolescent, 11 Internet-based modules based on CBT and IPT, and a follow-up MI in primary care to enhance behavior change targeting identified vulnerablity (e.g. personal risk profile) including depressive symptoms, dysfuctional thinking, and low peer and family social support. [33, 36, 69, 71-72] In this model (Figure 1), the primary care motivational interview is intended to enhance adherence/engagement (1), bringing about adolescent CBT/IPT and parent Internet-based components that alter individual, family and peer/school related vulnerabability/protective behaviors (2), reducing incidence of disorder (3), and enhancing developmental progress/functional status (4). In a subsequent pilot study, **Van Voorhees** and colleagues recruited 14 emerging adults (ages 18-24) from two urban primary care clinic settings who had at least one risk factor for depression (family or personal history of an episode). [126] Favorable trends were noted for the targeted risk factors between the pre/post measures: depressive symptoms, dysfunctional thinking, [127] [98] and low social suppport.

B. Development of CATCH-IT 3 and Phase 2 Clinical Trial: Based on review committee members, the original CATCH-IT intervention was revised and was titled CATCH-IT 3. In this model, motivational interviewing was employed to engage the adolescents in an Internet-based behavior change program. In the phase 2 clinical trial, **Van Voorhees** randomized adolescents into one of two versions of CATCH-IT 3: primary care physician (PCP) motivational interview + Internet program (MI) versus PCP brief advice + Internet program (BA). He hypothesized that the longer, more time intensive MI would be superior to BA in preventing depressive episodes. The intervention included an initial and follow-up interview in primary care, 14 Internet-based modules targeting risk behaviors, and an accompanying parent program developed by **Gladstone** and **Beardslee**. The MI group demonstrated superiority in adherence: use of Internet program (MI: 90% versus BA: 78%, p=0.12), total time on site (143.7 minutes versus 100.2 minutes, p=0.03), number of sessions (8.16 versus 6.00, p=0.04), longer duration of session activity (46.2 days versus 29.34 days, p=0.04), and with more characters typed into exercises (3532 versus 2004, p=0.01). Additionally, teens in the MI group reported higher trust in their physician (4.18 versus 3.74, p=0.05), greater satisfaction with the Internet-based component (7.92 versus 6.66, p=0.01), and lower hopelessness (MI group of 2% versus 15% for the BA group, p=0.044). Overall, the MI group demonstrated lower cumulative prevalence of depressive episodes (meeting criteria for MDD or being diagnosed and treated for depressive episode) at 3 month (4.65% versus 22.5% for the BA group, p=0.02) [29] and at 12 month (6.98 versus 27.50, p =0.013) follow-up (Figure 3 below). [128-129] Moreover, **Van Voorhees**, **Gladstone** and colleagues[128-129] reported that, on a measure of depressed mood (CES-D), scores fell in both groups from baseline to twelve weeks with statistically significant reductions sustained out to 52 week follow-up.[128-129] Similarly, **Van Voorhees** and colleagues found that adolescents in both groups demonstrated improvements in peer social support, declines in perceived impairment of school performance, and improvements in ratings of motivation.[30] The current version of CATCH-IT is freely available on-line and has a growing user base (http://catchit-public.bsd.uchicago.edu).

C. <u>Exploration of Mediation and Moderation:</u> Favorable baseline attitudes and beliefs and a preparatory motivational interview strongly predict Internet program use.[40, 130] Internet site use strongly predicts changes in vulnerability measures at 6 weeks and long-term mood scores (CES-D score declines by 1 for each 1,000 characters typed into exercises, p-value <0.001 at six months).

<u>Step 4: Large-scale field trials</u>. Although the present investigation is not yet at step 4 of the IOM research cycle, ultimately it will be important to know if the proposed intervention can be implemented successfully by different investigators across different states, and by pediatricians and office staff in a range of primary care practice types and settings. These investigators have experience conducting large-scale field trials as well. In the *Prevention of Depression Study (POD)* study, **Gladstone, Beardslee, Garber, Weersing** and colleagues[131] conducted a large-scale, multisite investigation of a group cognitive-behavioral prevention for at-risk adolescents. Across an 8 month follow-up, they found the rate and hazard ratio (HR) of incident depressive episodes were lower in the cognitive-behavioral group, versus the treatment as usual control group (21.4% vs. 32.7%, HR= 0.63, 95% Confidence Interval (CI): 0.40-0.97). [20] **Bell** and colleagues completed a large study of a universal resiliency building intervention targeting risk factors acquisition of HIV among black South African youth (N=531 children). The Collaborative HIV Adolescent Mental Health Program South Africa (CHAMPSA) intervention demonstrated significant improvements at the individual, family and community levels: general health ratings (ES=0.33), caregiver involvement (ES=0.40), neighborhood disorganization (ES=-0.313) as well as in multiple other measures.[24]

Figure 3: Cumulative Prevalence of Clinically Significant Depressive Episodes at 12 Months (p-value comparison < 0.013)

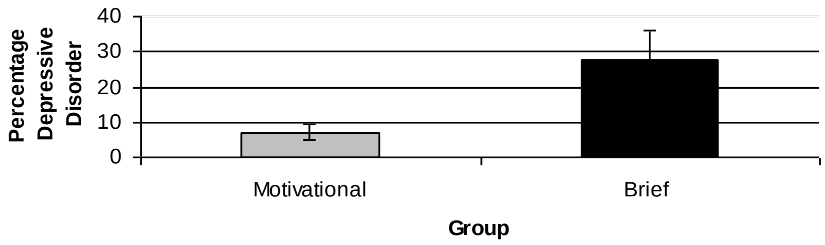

<u>Step 5: Facilitate large-scale implementation and evaluation of the preventive intervention program in the community.</u> "At this step, the investigator can provide a manual describing the program to guide implementation" (p.373).[119] **Beardslee** and **Gladstone** have developed a manual prevention program for families with parental depression, and they have been involved in the adaptation of this manual to new settings and populations.[22, 132] **Van Voorhees** has recently made his most recent version of the CATCH-IT intervention publicly available on the Internet (http://catchit-public.bsd.uchicago.edu) and is undertaking training programs in Texas, California and Illinois. The present project begins to address step 5 by implementing and evaluating a manual preventive intervention in real primary care settings across different states and differing healthcare systems. A completed implementation evaluation demonstrated that providers rated the CATCH-IT 3 intervention favorably and demonstrated competency in incorporating the intervention at 12 practice sites. [26]

<u>Feasibility: Studies supporting our ability to control quality and fulfill the aims of this application</u>

<u>Recruitment, subject retention, follow-up and analysis</u>: Both **Gladstone** and **Van Voorhees** have demonstrated the ability to recruit and retain subjects in complex prevention studies. **<u>Chicago</u>:** In the CATCH-IT K-08 sponsored phase two clinical trials, **Van Voorhees** recruited 12 primary care practice sites within five different health systems in four states and enrolled 83 adolescents with subsyndromal depressed mood by screening in primary care in 8 months. [29, 68] **<u>Boston/Wellesley</u>:** **Gladstone** and **Beardslee** [131] recruited 78 youth into a prevention study of offspring of depressed parents and retained 93% over 32 months. **Beardslee**, **Gladstone** and colleagues[18, 125] also recruited 105 families with parental depression and retained 87% at 4.5 year follow-up. **<u>Northwestern (Brown)/ Miami (Shi)</u>:** **Brown** and **Shi** have extensive experience analyzing data from RCTs of behavioral and preventive interventions.[133-135]

<u>Feasibility of study implementation in primary care sites</u>: **Van Voorhees** and colleagues recruited within five different health systems in four states (US Midwest and South). [62] Employing a public health model, patients were screened for major depression (depressed mood) and evaluated by phone to confirm inclusion (depressed mood) and exclusion (current major depression or other mental disorder) criteria. Of those screened positive and found eligible, 81% were enrolled.[29] Screening efficacy ranged from nearly 100% to 10%, with a mean of 60% of at-risk adolescents identified.

Treatment development and fidelity (including motivational interview): Investigators at both implementation sites have experience in the development and use of manual intervention programs. The CATCH-IT 3 program allows for collection use data every 6 seconds to make fine grained determinations of use adherence to particular components including time, text input, pages viewed, sessions, time of day, and ratings.[40] Similarly, the Chicago site has successfully recorded and analyzed motivational interviews for fidelity using standard methods (e.g. spirit and behaviors).[40] [136-137] [40] Ninety percent of adolescents in the motivational interview arm participated in the Internet program, and physician MI adherence with just one training session (versus three planned in this study) was adequate[40]. The Boston site has demonstrated the ability to implement interventions with high fidelity (POD fidelity of intervention delivery compliance rating scores ranged from 88.1% to 95.8%; PIP adherence ratings were 86.4% for the family meeting and 91.7% for the child session).[125]

Quality of assessments: In the CATCH-IT 3 assessments, outcomes were ascertained through blinded phone assessment interviews at six weeks and twelve weeks post randomization.[32, 138] Post study assessments were 91% complete at 6-8 weeks, 80% at 12 weeks, 78% at 24 weeks, and 74% at 52 weeks. In the POD study, outcomes were assessed at face-to-face assessment meetings. Independent evaluators were blinded to experimental condition throughout the study, and interrater reliability of depression ratings based on structured diagnostic interviews was 97.5% across the 8-month follow-up interval. In the PIP study, an interrater reliability study of assessor ratings of a semi-structured parent interview suggested excellent reliability, with intraclass correlations of 0.94 for total behavior changes, 0.92 for total attitude changes, and 0.93 for total changes.

4. Methods

a . Research Design

Summary: In this 5-year, multi-site randomized clinical trial, we propose to test the efficacy of the CATCH-IT (Competent Adulthood Transition with Cognitive-behavioral Humanistic and Interpersonal Training) primary care/Internet based depression prevention intervention against Attention Monitoring Psychoeducation (HEALTH EDUCATION) in preventing the onset of depressive episodes in an intermediate to high risk group of adolescents aged 13-18. We plan to (a) identify high risk adolescents based on elevated scores on the PHQ-A, a screening measure of depressive symptoms; (b) randomize 400 (200 per Chicago sites and 200 for Boston sites) of these at-risk adolescents into either the CATCH-IT or the HEALTH EDUCATION group; (c) assess outcomes at 2, 6, 12, 18, and 24 months post intake on measures of depressive symptoms, depressive diagnoses, other mental disorders, and on measures of role impairment in education, quality of life, attainment of educational milestones, and family functioning; and (d) conduct **exploratory** analyses to examine the effectiveness of this intervention program, moderators of protection, and potential ethnic and cultural differences in intervention response. This study will enroll adolescents, parents, primary physicians and office nurse/medical assistants who are related to adolescent participation. Parents will be enrolled as study subjects in order that we can obtain information from them with regard to their child and, their key potential moderators of intervention effect and ratings of their experience. Physicians and office nurse/medical assistants are enrolled to evaluate the feasibility and sustainability of the intervention.

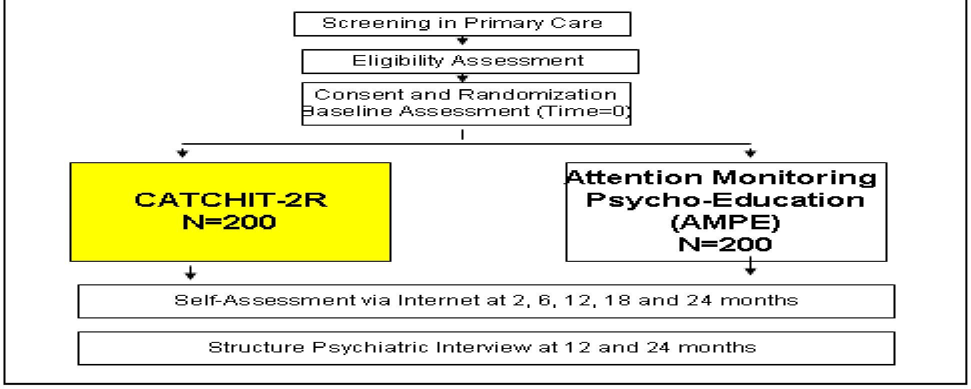

Figure 2: Study Design

b. Eligibility criteria

i. Adolescents

<u>Inclusion criteria</u>: (A) Youth ages 13 through 18 who are English speaking. (B) Youth must be experiencing elevated level of depressive symptoms on the Center for Epidemiologic Studies Depression[32] (CES-D) scale (score >/= 16) and have at least two core symptoms of Major Depression on the Patient Health Questionnaire, Adolescents.[139] If the score is >/=18 and no personal experience of depression, the cases would be discussed among investigators and the decision for inclusion in the study would be taken in a case by case basis. This definition of current subdiagnostic depressive symptoms is consistent with the criteria used by Radloff.[32] (C) Youth will be included if they have a past history of depression, anxiety, externalizing symptoms, or substance abuse. Youth presenting in partial remission from a major depressive episode at Baseline will be rescreened after 2 months utilizing the phone screen to ensure episode is fully remitted prior to randomization or access to study intervention. Those who do not fully remit after two months will be excluded.

<u>Exclusion criteria</u>: Adolescents must not have: (A) Current DSM-IV diagnosis (Kiddie Schedule of Affective Disorders)[44] of Major Depressive Disorder of Dysthymia, current therapy for depression, or be taking antidepressants (e.g., SSRIs, TCAs, MAOIs, bupropion, nefazodone, mirtazapine, venlafaxine); (B) Current CES-D score >35; (C) DSM-IV diagnosis of schizophrenia (current or past) or bipolar affective disorder; (D) Current serious medical illness that causes significant disability or dysfunction; (E) Significant reading impairment (a minimum sixth-grade reading level based on parental report), mental retardation, or developmental disabilities; (F) Serious imminent suicidal risk (as determined by endorsement of current suicide on CES-D or in KSADS interview) or other conditions that may require immediate psychiatric hospitalization; (G) Psychotic features or disorders, or currently be receiving psychotropic medication; or (H) Extreme, current drug/alcohol abuse (greater than or equal to 2 on the CRAFFT).

ii. Parents

Inclusion criteria: adolescent child enrolled in the study

Exclusion criteria: Non-English speaking or ineligible child

iii. Physicians/nurse practitioner

Inclusion: (A) primary care practices for minimum of 6 months, (B) willing to participate in training to perform interventions

Exclusion: (A) none

iv. Nurse/medical assistants

Inclusion: (A) practice physician participating

Exclusion: none

c. Justification for inclusion of any special or vulnerable populations

Inclusion of Children: The target age group for this intervention is adolescents ages 13-18. This is a high-risk age group for depression. The intervention requires the adolescent to participate in insight-oriented activities. Younger children may not have yet developed the cognitive ability to complete the exercises and adolescents older than 18 frequently may have already experienced an episode of depression. The goal of this intervention is to prevent the first episode.

Women, Children, and Minorities: Based on previous studies at our sites, we anticipate that about 60-70% of the adolescent participants will be female. Because of the emergence of gender differences in depression during adolescence (Hankin et al., 1998), we will test for gender effects in the present study, although in Clarke et al. (2001) there were no differences in the rate of change over time for males and females and no interactions between gender and treatment. We are not studying children younger than age 13 because the CATCHIT-2R intervention would require substantial adaptation for children's developmental needs. Moreover, the risk of depressive episodes is considerably lower for children below age 13 years. Our experience is that the ethnic ratio will reflect that found in the population in the cities in which the primary care practices are located (please see Target Enrollment Form Page). The geographic heterogeneity of the sites (i.e., Boston (Northeast), Chicago (Midwest)) should provide some degree of ethnic diversity. We assume an overall minority representation of about 15%, which is comparable to previous studies at these sites and representative of the populations in the communities served. We plan to over-sample minority participants.

d. Plans for subject selection, recruitment, and documentation of informed consent

i. Research Subjects Overview: Participants will consist of 580 adolescents' ages 13 through 18 and at least one parent (320 participantsin Chicago and 260 participants in Boston) who will participate in a baseline and five follow-up assessments (2, 6, 12, 18, and 24 months post baseline). 400 teens and 400 parents are anticipated to be eligible for randomization. All English-speaking ethnic minorities and Caucasians meeting inclusion criteria will be invited to participate. Adolescents will be eligible for participation if they are experiencing sub-threshold depression (> 1 symptom of depression, one of which must be a core symptom such as depressed mood, irritability or loss of pleasure for two weeks or more). We will include individuals with co-morbid anxiety disorders, attention deficit disorder, conduct disorder and past, but not current, substance abuse. We will exclude those who are too severely depressed for this form of intervention, or who would be unlikely to benefit from a primary care/Internet-based approach, such as those with severe mental illness (schizophrenia, bipolar disorder), ongoing active substance abuse, and those with developmental disabilities. Because this is a prevention trial, the participating youth will not have active major depression when they are enrolled, although some of them are expected to develop major depression or other mental health disorders during the study period. Parents will be enrolled as study subjects in order that we can obtain information from them with regard to their child and, their key potential moderators of intervention effect and ratings of their experience. Physicians and office nurse/medical assistants are enrolled to evaluate the feasibility and sustainability of the intervention.

ii. Adolescents:

Recruitment of research subjects: Participants will be identified using a public health model of screening (all adolescent patients during routine care appointments) for risk (sub-threshold depressed mood) in primary care clinics or specialty clinics (N=580, N=200 randomized from each site) encompassing diverse populations in urban, suburban, and rural areas in Chicago and Boston over 24 months. Based on our prior study, we expect that each site will need to screen approximately 5,000 adolescents in order to recruit and randomize 200 participants. We conservatively estimate that each physician will enroll 1-8 study participants, such that we plan to recruit 27-100 physicians at each site. We will recruit adolescent participants using a public health screening method at participating primary care practices. We will also have practices, if they desire, send out a template

letter to families inviting them to contact PATH directly for screening. Study team members will not have access to patient lists from the research sites.

1) Step 1: Screening: Adolescents will be invited to complete the screener while visiting their primary care doctor (Patient Health Questionnaire Adolescent, PHQ-A,[139]) as a matter of routine clinical practice. They will be offered the 2 question screener by a nurse, medical assistant, or study staff while in an exam room. Screening by medical assistant or nurse was successfully implemented in our phase 2 clinical trial and has been described in prior publications.[140 129] Those who screen negatively on the 2 question screener will have an option to have their contact information given to Northwestern for Project Tech (IRB#2012-0071). Potential cases will also be obtained via referrals from clinics by physicians, or through self-referral by brochures in the clinics where by parents can obtain information and call the study offices themselves directly. Flyers will be used and the flyer text posted on online forums such as Facebook or UIC websites. Participants will also be invited by a letter to contact the PATH team directly for screening if they are interested in participation. The letter will be provided by the PATH team to practices but sent and addressed by practices to avoid study staff having access to patient lists.

Step 2: Consent to be contacted by study staff with regard to study: All teens will be offered a study information brochure. The parent and adolescent will be asked if they wish to consent to have their screening form handed over to the study staff and to be contacted with regard to learning about the study and assessing eligibility. To do so, they must sign initial consent (parent)/assent (adolescent) at bottom of the form and provide contact information. The screener will then either be collected by the MA/RN or placed in a sealed envelope and returned to office or study staff present. If an envelope is used, it will state clearly "DO NOT MAIL. RETURN TO PATH STUDY. CONFIDENTIAL."

Step 3: After adolescent/parent assent/consent, primary care staff contacts the study coordinator and let them know they have signed initial consent forms. The study coordinator or a member of the research team would pick up the forms from the primary care setting.

Step 4: After obtaining initial consent from the parent and assent from the adolescent, study office conducts eligibility assessment (by phone) only to those adolescents who meet initial criteria specified on the 2 question screener. Up to five (5) call attempts are made in order to make initial contact with the participant(s). One call attempt may be replaced by a scripted email message if an email address is provided on the initial screening tool. A message is left on every other call. If no contact is made after 5 attempts, no further attempts to reach the participant are made.

Step 5: After establishment of eligibility criteria, the parent/adolescent are scheduled for enrollment/interview at their primary care office. If the teen is 18, they may attend without a parent if they desire (or if their parent is unavailable or unwilling to participate).

Step 6: Study staff meets adolescent and parent at primary care office, confirms full eligibility in face to face interview as well as consenting, enrollment and randomization of the subjects (described below). Should it be determined that a participant is in partial remission during the baseline interview, they will be rescreened by phone as described above in 2 months to determine if major depressive episode has fully remitted prior to randomization.

Step 7: Primary care physicians will be told to use usual care procedures (e.g., referrals for therapy, medication) in responding to adolescents with major depression during screening or during study participation. In addition, we will provide physicians with written materials and training so that they feel better equipped to manage adolescents who present with depressive issues.

Informed consent procedures for Adolescents: Potential research participants will be contacted initially by office staff in the physicians' offices. Office staff (Nurse/MA) or study staff will ask the adolescent and accompanying parent if they wish to complete an adolescent health questionnaire (2-question initial screen) that will be provided to their physician. At the bottom of the questionnaire, they will be asked if they are willing to be called by the study coordinator about the study. If they agree and are eligible based on the 2-question responses, they will be contacted by the study coordinator, who will speak with the teen and the parent about the study, conduct the phone eligibility assessment for the teen and will invite the teen and parent to meet. Those meeting inclusion criteria will be randomly assigned to the intervention or comparison group, after the meeting. At the first meeting, the study coordinator will meet with potential participants in a private office (within the physicians' office suites) and will provide a step by step review of the consent form with each participant. Parents and adolescents will have the entire study procedure explained to them, including the interviews, intervention, and questionnaires. Adolescents and parents will specifically consent to audio taping of interviews and retention of information for future studies (lack of consent to either or both of these items will not impact enrollment in the study). This is necessary to evaluate fidelity to the intervention interview guidelines. Both parent and child will complete written informed consent procedures. They will be informed that acceptance or refusal will not influence their ability to receive care from their pediatrician or other health care providers, and that they are free to withdraw at any time. Procedures for ensuring confidentiality and a statement of potential risks are included on the informed consent form. If the family chooses to enroll, they will complete the consent documents and will begin the baseline assessment. They will also be provided with copies of the consent documents to keep for their reference and retention. If a participant turns 18 during the course of the study, participants will be required to sign a new consent as a legal adult. A copy of the consent and HIPAA will be mailed to them for their review. A self-addressed and stamped envelope will be included for them to return the forms to UIC. If their birthday coincides with a scheduled in-person meeting with study staff, a hard-copy signature will be obtained.

iii. Parents

Recruitment and consent of research subjects: Parents will be consented in parallel process with adolescents. The procedures are the same, except parents don't need to complete an eligibility assessment over the phone, since their eligibility depends on their teens' eligibility.

iv. Physicians and nurse practitioners (acting as primary care provider):

Recruitment and consent of research subjects: Potential physicians or nurse practitioners will be recruited via provision of general information about past studies and current plans. The initial approach will be made by the site investigator at the participating organizations. After initial interest is established, the principal investigator will visit the practice during lunch time (lunch provided) to explain elements of participation. If physicians/ nurse practitioners agree to participate, they will have the risk, benefits and study procedures reviewed with them. Physicians/ nurse practitioners will specifically consent to audio taping of interviews. This is necessary to evaluate fidelity to the intervention interview guidelines.

v. Office Nurse and medical assistants:

Potential office nurses/medical assistants/practitioners will be recruited via provision of general information about past studies and current plans at an initial lunch time meeting that includes the practice physicians and nurse practitioners. Investigators will visit the practice during lunch time (lunch provided) to explain elements of participation. If physicians/ nurse practitioners agree to participation, they will have the risk, benefits and study procedures reviewed with them.

e. Description of Procedures

Random assignment to experimental conditions: Participants will be assigned randomly to CATCH-IT 3 or HEALTH EDUCATION. Randomization will be blocked by practice and stratified by level of risk, and will be balanced across time. [141-142] Blocked randomizations will be prepared by the Northwestern site (data analysis site).

Intervention

- **I. CATCH-IT 3 intervention defined.** Based on promising results from the phase 2 trial of CATCH-IT 3, a modified version of this primary care/Internet-based preventive intervention is being prepared (text revised and edited, Internet design completed,) titled CATCH-IT 3 revised (CATCH-IT 3), using previously published methods. [25, 38, 143-144]The CATCH-IT 3 intervention has a motivational (3 PCP motivational interviews at time 0, 2 months and 12 months and 2 check-in phone call at week 1 and week 4) and an Internet component (with separate adolescent [14 modules] and parent [5 modules] programs). Parents have a parallel structure of motivational (3 motivational interviews at time 0, 2 months and 12 months, performed by study coordinator) and 2 phone calls at weeks 1 and 4 after enrollment) This revised and expanded intervention will include a comprehensive approach to reducing modifiable risk factors and enhancing resiliency factors associated with increased or decreased risk of depression, respectively, proposed by Spence and Reinecke.[145] Study staff will monitor time in study and deploy elements of the intervention based on time since enrollment, including computer and human elements (e.g. calls, doctor visits). If study staff are unable to reach participants by phone on the first attempt for any of the above mentioned study procedures, staff will attempt to call two (2) more times over a 1-2 week period to establish contact. If no response is received, staff will try contacting participants by a secondary method as specified in the General Information Sheet (email, mail, or text message). Staff will then wait 2 weeks before contacting the participant again. This process will be repeated until contact with the participant is made or the participant requests to be withdrawn from the study. If withdrawal is requested, a letter confirming their withdrawal will be sent. Details regarding contact of participants and withdrawal procedures can be found in the **Protocol for Contacting Participants** and **Withdrawal Procedures**.

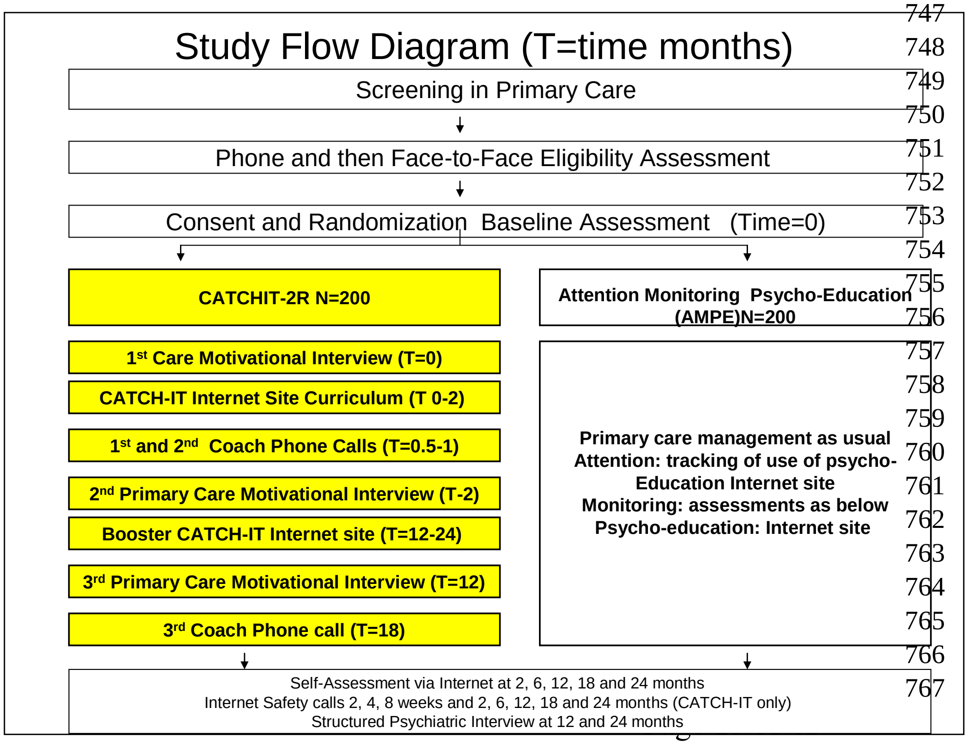

Roles of study and primary care staff in study implementation. Practice nurse/medical assistant staff members will complete the screening process. Study staff will collect completed screening forms from each practice and will conduct eligibility assessments by phone.[29] Consent and baseline assessments will be conducted face-to-face in primary care offices. At each site, primary care physicians willing to participate and complete 3 training sessions (during lunch) and meet fidelity standards will be recruited and will perform adolescent motivational interviews in person or by phone. The study coordinator will perform the parent motivational interview

while the adolescent meets with the physician, and he/she will also perform the telephone coaching calls.

Motivational component: The intervention uses motivational interviewing, goal-setting, and telephone coaching to enhance each of these change processes and to advance the participants (adolescent and parent, separate interviews) through the stages of change to reduce vulnerability and increase protective factors.[112, 146 147-148] In the motivational interview (10-15 minutes duration), the physician (study coordinator in case of parent) seeks to help the adolescent weigh the balance of positives and negatives of undertaking this depression prevention intervention. Before meeting with the PCP or study coordinator, the adolescent and parent respectively complete or review a self-administered MI form intended to help them prepare participation. If the motivational interview is to be completed by phone, these documents can be emailed to them prior to the meeting time. The coaching phone calls will be conducted by study staff, use the same motivational interview approach, and last 5 minutes or less in duration, solely designed to encourage completion of the intervention and behavior change (not act as psychotherapy). All adolescents complete a motivational interview questionnaire before the physician interview to enhance level of participation and fidelity to the interview (asks questions physician will ask).

Internet adolescent component: Table 2 below describes the modified intervention in terms of theoretical framework, content, exercises and targeted risk factors. Each module has exercises that participants can complete online and print out for review. We have previously described the development of the internet intervention based on the Instructional Design Model, Trans-theoretical Model of Change, and the Theory of Planned Behavior.[25] With the exception of cultural modules, all materials have already been developed. To enhance participation, CATCH-IT 3 will include engaging media options such as music, games, stories, videos, and the opportunity to post stories for future users (asymmetric communication, not for current study users).[25, 62, 130]. For participants without internet access at home, appointments can be scheduled with study staff to use computers at their clinic site or UIC. A private space and tablet computer will be provided for their use at no cost. CATCH-IT teens will additionally be offered a booster website after completing 2 months of the CATCH-IT program. Adolescent consent, adolescent assent, and parent permission will be obtained over the phone.

Table 2. Intervention Phases and Components

Component	Module Content and Exercises (theoretical model)	Behavioral Target
Induction (month 0-2) and Maintenance Phase (month 2-6) using Standard Modules		
Motivational Component	PCP MI at time 0,8 weeks and 12 months<br>Phone calls as 1 and 4 weeks	Under-attainment of milestones<br>Low motivation for prevention
Modules 2 -4	Event scheduling<br>Practicing active behaviors (Behavioral Activation) BA	Loss of response contingent Reinforcement
Modules 5-8	Identifying and countering pessimistic automatic thoughts, general beliefs and hopelessness<br>Problem solving (Cognitive Behavioral Psychotherapy)	Cognitive distortions<br>Pessimistic cognitive style/content<br>Poor coping skills
Modules 9-12	Improving communication skills, coping transitions conflict resolution. Engaging new networks (Interpersonal Psychotherapy –IPT)	Lack of social support<br>Social skills deficits<br>Lack of peer support
Module 13-14	Flexibility/humor/persistence, Community involvement<br>Barriers to treatment (Resiliency concept)	inflexible responses<br>Low levels of pro-social activities
Booster program 6 modules Situational problem solving	Rational appraisal of problems, solutions, plan and execute and focus on addressing challenges in achieving healthy developmental milestones in relationships and academics for example.	Unresolved conflict<br>Low self-efficacy

Internet parent component: The parent component of the intervention is based on an adaptation of Beardslee and Gladstone's clinician-facilitated and lecture intervention approaches from the Preventive Intervention Project.[18] This intervention helps parents develop the awareness and skills needed to help build resiliency in their children. The intervention also seeks to reduce known risk factors for adolescent depression. Gladstone and Van Voorhees have developed an intervention plan (Table 3). To address the key mediating role of parental depressed mood, a personalized component will be available to parents with depressed mood.

For participants without internet access at home, appointments can be scheduled with study staff to use computers at their clinic site or UIC. A private space and tablet computer will be provided for their use at no cost.

Table 3. Parent Program

Component	Module Content and Exercises (theoretical model)	Behavioral Target
Modules 1-3<br>PIP intervention	Activism<br>Connectedness<br>Affect recognition	Cultivating strengths<br>Encourage discussion, behavioral activation<br>resiliency behaviors and expression of emotion
Module 4 -5<br>If parental depressed mood	Treatment education<br>Access to MoodGym depression treatment program	Increase likelihood of treatment<br>Self-directed CBT and IPT via MoodGym to change behaviors [149-150]

<u>Training health care providers:</u> Physicians will be trained to implement the study during three lunchtime sessions (N=3 at time=0, 2 months [feedback] and time=12 months [booster]). This training program adds two sessions to the program successfully introduced in the CATCH-IT 3 study, with physicians demonstrating satisfactory fidelity to the motivational interview model[29]. All physicians will be provided with standard scripts to assist them during the interviews, and all interviews will be taped and reviewed for fidelity. We will assess physician ability to perform interviews after session three and provide feedback based on review of taped interviews. [108, 136-137, 151]

J. <u>Attention Monitoring and Psychoeducation Group ("Healthy Lifestyles") defined</u>: The HEALTH EDUCATION components (labeled as "Health Education" to participants) are similar to those employed in previous primary care based quality improvement/Chronic Care Model Interventions (patient education [psycho-education described below], provider training [described in Case Finding and Recruitment], active monitoring and referral [case management, discussed under assessments], physician and nurse education and routine contact with PCP [study design rationale]) (see Table 4 below). This Internet site will focus on assisting parents and adolescents with general information on health living appropriate for age as well as psychoeducation about depression. The psychoeducation is intended to assist participants in determining what depressive illness is and what treatment option exist and increase likelihood of seeking treatment.[25, 152-153] The HEALTH EDUCATION group will receive the same assessments as the CATCH-IT 3 group (including notification and referral) and routine contact with PCP (anticipated 2-3 visits). Psychoeducation Internet site use will be monitored to compare with use of the CATCH-IT 3 intervention as an attention control. Self-harm risk will be managed based on a standard protocol. If study staff are unable to reach participants by phone on the first attempt for any of the above mentioned study procedures, staff will attempt to call two (2) more times over a 1-2 week period to establish contact. If no response is received, staff will try contacting participants by a secondary method as specified in the General Information Sheet (email, mail, or text message). Staff will then wait 2 weeks before contacting the participant again. This process will be repeated until contact with the participant is made or the participant requests to be withdrawn from the study. If withdrawal is requested, a letter confirming their withdrawal will be sent. Details regarding contact of participants and withdrawal procedures can be found in the **Protocol for Contacting Participants** and **Withdrawal Procedures.**

Table 4. HEALTH EDUCATION Psychoeducation Program Adolescents

Component	Module Content and Exercises (theoretical model)	Behavioral Target
1	AVOIDING ACCIENTAL INJURY	Safety behaviors
2	NUTRITION FACTS AND MYTHS	Healthy diet
3	HEALTHY EATING ON THE GO	Healthy diet
4	HEALTHY TEETH AND GUMS	Dental health

5	BENEFITS OF CALCIUM AND VITAMIN D	Calcium and Vit D consumption
6	ENVIRONMENTAL HEALTH	Reducing exposure
7	FOODBORNE ILLNESSES	Reducing exposure
8	PROTECTION AGAINST SEASONAL ILLNESSES	
9	MENINGOCOCCAL MENINGITIES VACCINATION	
10	BICYCLE AND VEHICLE SAFETY MEASURES	Increase bike safety
11	FIRE SAFETY IN THE HOME	Increase fire safety
12	HUMAN PAPILOMA VIRUS VACCINATION	Increase vaccination
13	ALLERGIES	Minimizing exposure
14	LEARNING ABOUT MOOD	Awareness of when to seek treatment

Table 4. HEALTH EDUCATION Psychoeducation Program Parents

**Component**	**Module Content and Exercises (theoretical model)**	**Behavioral Target**
1	NUTRITION AND TEENS: HELP FOR PARENTS	Healthy Diet
2	VEHICLE AND BICYCLY SAFETY MEASURES FOR YOUR TEEN	Safety Behaviors
3	SNACK HEALTHY-TIPS FOR YOU AND YOUR TEENS	Healthy Diet
4	YOUR TEEN AND THE WORKPLACE	Safety Behaviors

K. <u>Blindness of clinical evaluators</u>: Clinical evaluators will be unaware of the intervention condition to which participants are assigned. The research administrator will access the randomization program, which will make the assignment to intervention groups, after the baseline assessment is completed. After baseline, evaluators from the Boston site will assess Chicago participants, and Chicago evaluators will assess participants from the Boston site. We will assess the quality of the blind by asking the clinical raters to guess treatment at each assessment point. Dr. Gladstone will monitor assessment quality at both sites.

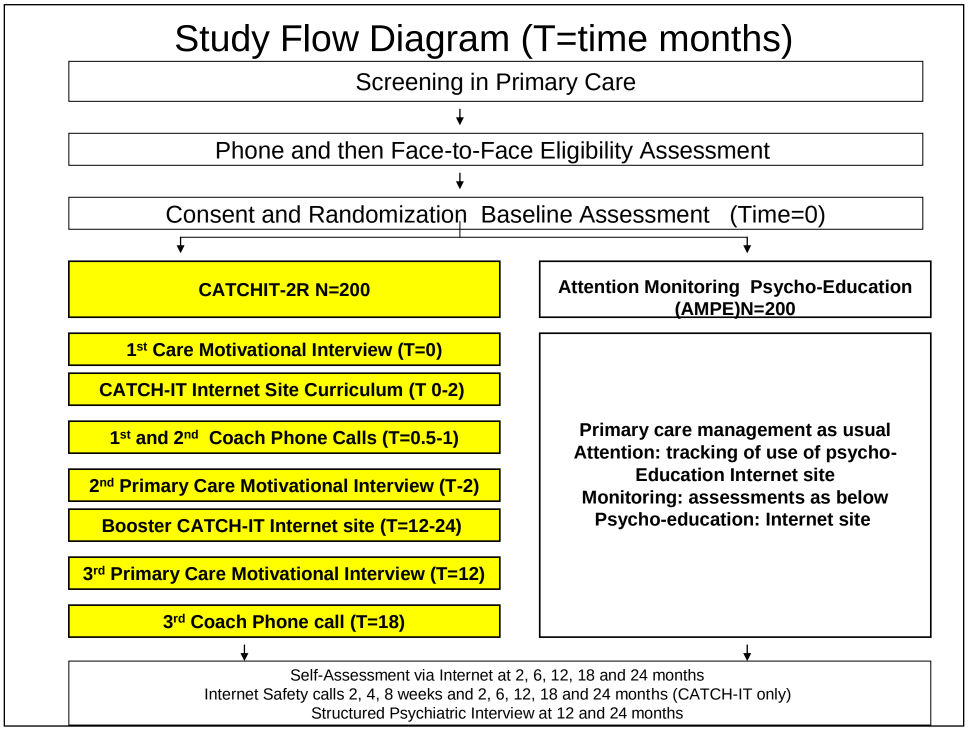

L. <u>Assessment instruments</u>:

Please see Table 5 (below) for an overview of the assessment schedule as well as the study flow diagram which provides an overview of assessments. Assessments of adolescents and parents will occur at baseline and at 2 (self-assessment and phone interview), 6 (self-assessment and phone interview), 12 (self-assessment and phone interview), 18 (self-assessment) and 24 (self-assessment and phone interview) months post-baseline. Self-assessments can be completed online, but if preferred by the participant, can instead be completed in person or by phone. Surveys of office staff will occur at baseline and 1 year post enrollment. Interviews between adolescents, parents, and providers will be taped at the time of enrollment and follow-up. For self-assessments, parents and adolescents (and their parents) will be notified within 48 hours if they have severe depressive symptoms consistent with major depressive episodes. Table provides an overview followed by a complete list.

Table 5. Assessments and Instruments (Adolescent and Parent)

Domain	Form	Month in Study					
		0	*2*	*6*	*12*	*18*	*24*
Psychopathology and Treatment (interview)	*KSADS* (baseline)[44] (depression and anxiety modules, screens for bipolar d/o and psychosis) and *KLIFE* (2, 6, 12 and 24 months), [154] and Treatment history *(CASA)* [155]	P,A	P,A	P,A	P,A		P,A
Vulnerability Symptoms (self/other-report)	*Depression Scale (CES-D, 20 items, alpha=0.86)*[138], administered both verbally and written [34]. For adolescents only, anxiety will be assessed by the *SCARED* (41-items), with good reliability/validity.[156] Externalizing in teens will be assessed by the *DBD*-parent form (45 items).[157]	A<br>P*<br>P	A<br>P*P	A<br>P*P	A<br>P*P	A<br>P*P	A<br>P*P
Vulnerability-cognition and social factors (self-report) and adverse events	*Beck Hopelessness Scale (BHS*[160] ,20 items); *CRAFFT* (6 items, alpha=0.68)[161] *ALEQ (life events)*[162] *Sibling Inventory of Differential Experience (SIDE)*	A	A	A	A		A
Functionality	*World Health Organization (WHO) Quality of Life Scale (26 items)*	P		P	P		P
Family dynamics (self-report)	*Sibling Relationship Questionnaire (48 items)*	P,A			P,A		P,A
Family dynamics (self-report)	*Child's Report of Parental Behavior Inventory (23 items)*	P,A	A		A		P,A
Family dynamics (Self-report)	*Conflict Behavior Questionnaire (20 items),*	P,A		P,A		P,A	

Developmental milestones (interview)	*Social Adjustment Scale (36 items)*	A			A		A
Personalization - Motivation	*Theory of Planned Behavior Scale* (21 items, alpha=0.76 and *Trans-theoretical Model scale* (4 items, alpha =0.76)	A			A		A
Experience/Use	training (4 items, alpha=0.96)	Continuous measure					
Adherence (use) and log files	Time measured every 6 seconds, number characters typed in, completed exercises and log files of clicks/actions patterns[164]	Continuous Measure					
Practice Functionality	Safety Net Medical Home Sale	Completed by Health Care Administrator at Baseline only					
Physician and Nurse/MA Implementation	Evidence Based Practice Attitude Scale (15 items), Revised Organizational Readiness For Change (51 items), Culture of Medical Group Practice – revised (16 items)	Completed by physicians and nurses/medical assistants (organizational readiness only) at 2 months					
Physician and nurse questionnaire with domains in feasibility and sustainability, as well as the Revised Organizational Readiness For Change		Measured only after the completion of the study.					

Legend: A-Adolescent, P-Parents on adolescent's site and P*-Parents on their site

List of Assessments

Adolescent Measures

Demographic Information: Demographic information will be obtained at baseline from adolescents and parents using the General Information Sheet (GIS) covering age, date of birth, sex, height, weight, address, phone number(s), email address(s), race/ethnicity, school placement, parental marital status, family composition, number of siblings and first-born status, and number of lifetime moves, and, derived from the Four-Factor Hollingshead measure of social status.[166]

General Information Sheet – Adolescents and parents will provide demographic information about themselves at baseline and follow-up assessment points. Information collected includes age, date of birth, sex, height, weight, address, phone number(s), email address(s), race/ethnicity (teen only), school placement (teen only), parental marital status (teen only), family composition (teen only), number of siblings and first-born status (teen only), and number of lifetime moves (teen only) derived from the Four-Factor Hollingshead measure of social status.

KSADS – The KSADS is a semi-structured clinical interview that is used to assess psychiatric diagnoses in participants under age 18, specifically for depression and anxiety, and provides screening interviews for bipolar disorder and psychosis. This instrument is used to interview both the child and the parent about the child's current and past psychiatric diagnoses.

KLIFE – This instrument will be used to assess these same diagnoses since baseline and will be administered to both children and parents at the 2, 6, 12 and 24 month follow-ups. The KLIFE measures the occurrence, degree of severity of new episodes, and the extent of impairment using the Depression Rating Scale (DRS), which yields a depression score from 1-6 for each week of the follow-up interval.

CES-D – The Center for Epidemiological Studies – Depression Scale is a self-report measure of the frequency of 20 depressive symptoms over the past week, using a 5-point scale. The use of self-report scales like the CES-D as depression case-finding or screening instruments has been successfully validated with both adults and adolescents. The CES-D is short and easy to read, has been successfully administered in several large adolescent school samples, and has strong psychometrics with youth.

SCARED – The SCARED is a 41-item scale, with five factors corresponding to different diagnostic categories of anxiety, and with good reliability and validity. Anxiety is often comorbid with depression and may predict either a better or worse response to the intervention.

DBD – The Disruptive Behaviors Disorder Scale is a self-report measure assessing the level of teens' behavioral problems.

Beck Hopelessness Scale (BHS) - This is a 20-item, true-false measures the extent to which individuals are pessimistic about their future. The BHS has been shown to predict dropout from psychosocial treatment and poorer treatment response. The BHS has strong psychometric properties in adolescent samples.

Conflict Behavior Questionnaire - A self report questionnaire evaluating relationship experiences and conflict between parent and child (both mother and father)

World Health Organization (WHO) Quality of Life Scale – This is a 26-item scale to assess an individual's perceptions regarding their quality of life within the context of their culture and value system, personal goals, standards, and concerns.

Sibling Relationship Questionnaire (SRQ) – A self-report measure containing 48 questions evaluating sibling relationships and support.

Social Adjustment Scale (SAS) – A self report measure containing 36 items. The measure is designed to evaluate behaviors at school, with peers, and at home over the past two weeks.

Teen Behavior Questionnaire (TBS) – a self report questionnaire regarding diet, exercise, religion, and internet use.

CRAFFT – This is a 6-item measure that assesses non-alcohol illicit substances.

ALEQ (life events) – The Adolescent Life Events Questionnaire is a retrospective self-report measure that asks the teen to report on the presence/absence of various life events in the following categories: health/loss, conflict/arguments, changes/moves, school/job, finances, and crime and legal issues.

Theory of Planned Behavior Scale – We adapted the items from a previous questionnaire that was adjusted based on the preventive health model. The original instrument, developed for prostate cancer, was adapted to

primary care based depression prevention. The participants will indicate a level of agreement based on a Likert-type scale of 1=strongly disagree, 2=disagree, 3=neither agree nor disagree, 4=agree and 5=strongly agree with items such as “depression intervention makes sense to me.”

Trans-theoretical Model Scale - We will measure motivation to change risk-factor behaviors before, during, and after the intervention. We adapted the standard approach for measuring motivation as described by Miller and Rollnick to evaluate importance (“rate the importance of preventing a depressive episode”), self-efficacy (“rate your ability to learn coping skills to reduce your risk of depression”) and readiness (“rate your readiness to learn coping skills”) on a 1-10 scale (1, “not important”, 10 “very important).

Child’s Report of Parental Behavior Inventory (CRPBI) – A scale used to evaluate a teen’s perceptions and experiences of both mother and father.

Sibling Inventory of Differential Experience (SIDE) – A scale to measure a teen’s perceptions of parent (both mother and father separately) responses to the teen as compared to their siblings.

Motivational Interview Pre-Measure - This measure is administered to adolescents prior to meeting with the physician for a motivational interview. It contains items asking adolescents to identify goals for the intervention, consider how they are feeling and their degree of motivation to change their feelings, and their willingness to engage in the present intervention. This is an optional measure.

Suicide Ideation Scale - A scale used to assess the adolescent’s current level of suicidal tendencies. This measure is only used in response to adolescent reports of suicidal thinking.

Suicide Intent Scale – A measure used to inquire about past suicide attempts. This measure is only used in response to adolescent reports of suicidal thinking.

Lethality Scale – A measure of the lethality of past suicide attempts. This measure is only used in response to adolescent reports of suicidal thinking.

Safety phone calls- Assessment calls to evidence self-harm ideation and adverse experience with Internet program. Qualitative questions regarding website and baseline interview are also included.

Parent Measures

KSADS - Please see description above.

KLIFE - Please see description above.

CES-D - Please see description above. Parents will complete the CES-D regarding their own mood, and also will complete the CES-D about their adolescent.

Conflict Behavior Questionnaire - A self report questionnaire evaluating relationship experiences and conflict between parent and child

DBD – The Disruptive Behaviors Disorder Scale is a self-report measure assessing the level of teens’ behavioral problems.

Child’s Report of Parental Behavior Inventory - Parent (CRPBI) – A scale used to evaluate a parent’s perceptions and experiences of their child.

Sibling Relationship Questionnaire (SRQ) – A self-report measure containing 48 questions evaluating sibling relationships and support.

World Health Organization (WHO) Quality of Life Scale – This is a 26-item scale to assess an individual's perceptions regarding their quality of life within the context of their culture and value system, personal goals, standards, and concerns.

Suicide Ideation Scale - Please see description above. This measure is only used in response to adolescent reports of suicidal thinking.

Suicide Intent Scale - Please see description above. This measure is only used in response to adolescent reports of suicidal thinking.

Lethality Scale - Please see description above. This measure is only used in response to adolescent reports of suicidal thinking.

The Child and Adolescent Services Assessment (CASA): This measures use of medical services by adolescent.

Physician/Nurse Measures

Physician and Nurse Feasibility Questionnaire: We developed a physician and nurse feasibility instrument based on interviews with providers and primary care observation (willingness used with permission of My K Banh, Ph.D). We developed these measures based on the Van Voorhees practice experience and discussions with office staff. They were then used to evaluate practice experience with the intervention in the recently completed randomized trial. Willingness and capability predicted practice performance of key functions such as screening.
Feasibility *(alpha=0.83 for physicians, 0.94 for nurses)*: This construct was developed to gather information regarding specific elements of the program. Providers were asked to provide answers to each statement using a five-point scale from 1 (strongly disagree) to 5 (strongly agree). Physicians and nurses are surveyed on elements of the process to screen for depression, and about the motivational interviewing process and the Internet modules. Group means and standard deviations for each statement are generated.
Willingness *(alpha=0.86 for physicians, 0.78 for nurses)*: Physicians and nurses provide opinions regarding the responsibility of primary care providers to care for adolescent depression. Each statement begins with "I believe that primary care providers should. . ." followed by a statement (e.g., "ask directly about depressive symptoms during well-child visits"). Statements are rated on the five-point scale from 1 (strongly disagree) to 5 (strongly agree). Group means and standard deviations for each statement are generated.
Capability *(alpha=0.77 for physicians, 0.78 for nurses)*: Providers are asked to provide opinions regarding their abilities to provide depression-related services to patients (e.g., "I have adequate knowledge about adolescent depression"). Providers answer on a five-point scale from 1 (strongly disagree) to 5 (strongly agree), with group means and standard deviations generated for each statement.
Sustainability *(alpha=NA, only one item)*: On a five-point scale from 1 (strongly disagree) to 5 (strongly agree), providers are asked, "I would implement the entire program (interview and referral to Internet website) in my office even if I were not enrolled in the study." Nurses were asked additionally, "I would recommend the intervention to my child or the child of a close friend." Group means and standard deviations are generated for each statement. The implementation statement also serves as the outcome measure for the willingness and capability statements.

Medical Home Safety Net Scale: Assessment, recently developed a the University of Illinois at Chicago, measures practice adherence to the medical home model.

M. <u>Quality assurance over assessment</u>. Interviewers will have extensive clinical experience with adolescents and with administration of the Kiddie Schedule of Affective Disorders (K-SADS). A computer-based algorithm (Chicago) will determine periodicity of assessments. We estimate the total time for each phone assessment to be 30 minutes and total assessment time to be < 2 hours, which is substantially less than the total duration of activity time with the intervention (1 hour provider, 3 hours Internet).

N. <u>Incentives to participants</u>. Adolescent and parent participants will receive small monetary incentives to compensate them for their time and travel expenses ($200 over 2 years to adolescent, $100 over 2 years to parent). Participants will also receive reimbursement for travel up to $20 in value. Physicians will be compensated through their practices based on pro-rated reimbursement (to practice) for time devoted to study participation (training, interviews etc) at $200 for each HEALTH EDUCATION participant and $400 for each CATCH-IT participant. Nurses and medical assistants will receive $100 over 2 years. Should the participant not wish to receive a cash payment, be unable to collect a payment in person, or prefer a payment be mailed, incentives will be mailed or emailed to the participant in the form of check or gift card. Small gift cards (valuing $5) will be provided to teen participants for module completion (valuing $20 in total). These will be mailed to each participant. When teens turn 18 years old in the study, they need to sign reconsent forms to continue their participation. If teens wish to continue in the study, they will receive $50 for signing their consent and HIPAA forms. This can be given to them in the form of cash or a giftcard. Teens will receive an additional $35 in the form of a Tango gift card for completing qualitative questions about their experience with the PATH study after 24 months.

**Time (month)**	**Key Task**	**Milestone**
0-6	Study preparation	Training of sites and final functional tests of Internet intervention
7-12	Start-up all sites	Enroll N=80
13-24	Enrollment	Enroll N=160 total N=240
24-30	Enrollment and follow-up	Enroll N=160, total N=400, complete follow-up N=80
30-42	Follow-up completion	complete follow-up N=80, complete follow-up N=80, total N=160
		complete follow-up N=80, total N=240
36-48		complete follow-up N=80, total N=320
49-60	Analysis and follow-up	complete follow-up N=80, total N=400

<u>Qualifications of investigators</u>: This group of investigators has made important contributions to the field in studies of risk factors for adolescent depression (**Van Voorhees**, **Gladstone**, **Beardslee**, **Bell**),[47, 49, 78, 80, 81] the prevention of depression in adolescents (**Van Voorhees, Gladstone**, **Beardslee**, **Bell, Weersing, Garber**)[15, 20, 22, 24, 40, 44, 50], the development of technology-based interventions (**Van Voorhees**, **Munoz**, **Simon**), cognitive behavioral psychotherapy (**Reinecke**), and analysis and design of randomized controlled trial and hypothesis testing **(Brown)[1, 201, 202].** Members of this group have worked together on other prevention studies of youth depression. **Van Voorhees** is a recognized expert in the field of primary care mental research with adolescents[131] and depression[5] and has demonstrated the ability to field three clinical studies of primary care/Internet based interventions.[43, 203] **Gladstone** is an expert on preventing youth depression, with a particular focus on family-based prevention approaches. **Brown** and **Shi** are internationally and nationally recognized experts in evaluation of preventive mental health interventions and understanding trajectories and outcomes in adolescents.[1, 3 184, 185] **Canel** (MD, Pediatricis, Physician Leader, Practice-Based Research Network, Northshore Health Systems) and **Eder** (Ph.D. Cultural History) have extensive experience in developing and implementing primary care interventions as experienced healthcare administrators. **Cheng has**

extensive experience directing primary care studies at Harvard-Vanguard. **Desrosiers** will play key role in assisting implementation in UIC adolescent medicine clinics.

i. Data analysis: Descriptive statistics will be calculated for the variables. As appropriate, the Pearson chi-square test, Fisher's exact test, or analysis of variance will compare the CATCH-IT 3 and HEALTH EDUCATION groups for balance on baseline characteristics, including demographics and prior treatment history. While randomization should be successful, any variables significantly differing between groups will be included as a covariate in the analyses, along with those we expect to be related to depressive disorder (i.e., gender), and those we find to be empirically related. STATA, SPSS, SAS, and Mplus will be used for the analyses.

Survival analyses: We will test for constant hazard over time and if not significant at 0.10 will use Cox proportional hazards to compute the hazard ratio for major depressive episodes for the intervention group in comparison to the HEALTH EDUCATION group. If the test or diagnostics reveal non-proportional hazards, i.e. there is evidence of differential effect over time, i.e. 1-year vs 2-year outcomes; we will include this non-proportionality in analyses. We have used such limited "model fitting" tests without regard to the magnitude of intervention effect in previous analyses that still preserve the Type I error rate[167], and will follow multiple comparison procedures we have previously used to identify time intervals where significant effects are found.[168]

Longitudinal data analysis: Our general method will be to use growth curve modeling with continuous outcomes to examine how the intervention changes depressive symptoms, vulnerability factors, educational impairment and quality of life. Simple comparisons will be calculated as appropriate (e.g. number of depressed days),[34] and survival curve modeling will be used for binary outcomes for prevalence of disorders. [24, 169-173] Guided by our methods development on growth models, we will address missing data using full information maximum likelihood and multiple imputation, which provide improvement over "last observation carried forward" and other less refined methods[174-176]. We will also examine whether there are variations in growth trajectories by introducing covariate interactions and mixtures to growth models, techniques that we and our colleagues have developed [167, 177-180]. These more complex models can characterize quantitatively differential response as well as qualitative changes such as recurrence. All such models will be preceded by careful model checking. [152, 167, 181-182]

Mediator analysis: We will evaluate potential mediators including change in motivation, principles (intervention experience characteristics) of effective preventive interventions (adherence/dose, positive relationships, training and socio-cultural relevance) and changes in vulnerability and protective factors (social support, automatic negative thoughts, depressed mood). Based on our recent work on a comparison of analytic models for mediation with nonlinear models, [183] we will use the "product of coefficients" method to conduct tests of mediation and the bootstrap method for accurate confidence intervals [184]. To examine the effects of participating in the website, we will conduct complier average causal effect (CACE) analyses that include covariates predicting participation status[185-188].

Moderator analysis: We expect to conduct tests of moderation in six domains: (1) demographic and cultural factors, [35-37](2) vulnerability factors and adverse events, [33] (3) motivation and attitudes, [38] (4) physician relationship, (5) parent and child co-morbid psychopathology, and (6) treatment before and during study. [20, 39] We will use sub-group analyses and tests of interaction to evaluate moderator effects.

Sample Size and Statistical Power

Aim 1: We conducted a series of power/sample size calculations through a SAS macro (Shi, 1995) under realistic alternatives to address impact on MDE. We require 200 randomized subjects per intervention condition to achieve 80% power based on a conservative application of our pilot study findings from the CATCH-IT trial.[129] These calculations assume that in the control group 72% are free from depression after one year follow up and the second year continues to follow the same exponential rate for controls; for intervention the hazard is a constant ratio of 0.62, and an attrition rate of 7% for each or the first 4 quarters and 2% for each of the second 4 quarters. Note that the estimated hazard rate of 0.62 is 2/3 of that we observed in this previous study. There are concerns raised about the use of pilot study data to assess power, even using a

lower value than we observed [189], so we conducted other power calculations based on the data from a similar study.[19] In their study, they found a departure from exponential and disproportional hazards of intervention over time. Assuming the same attrition rate as we found above, a study with 400 randomized subjects would produce 80% power when the effect of CATCH-IT 3 was only 60% at every interval of time relative to the CBT hazard rate in the Clarke's study. Thus we expect to have sufficient power even if the overall benefit is 60% that obtained by Clarke.

Aims 2 and 3: We will examine changes in the growth curve for depressive symptoms and quality of life over 6 measures. We have 80% power to detect a modest effect size of 0.25 by the last time point, accounting for 75% reliability in the intercept (www.psmg.usf.edu/PowerCal/case0.html).

Aim 4: Using mean and standard deviation values from our previous educational impairment data as an illustration of moderator analyses[30] with M=11.3 (SD=3.6) and M=9.9 (SD=3.4), with power of 0.80, alpha level of 0.05, and 10% attrition, 109 participants are needed for each group for a total of 218 participants. Thus we have sufficient power to detect moderate to large interactions with intervention.

Exploratory Aim 1 will consist of calculation of descriptive statistics. For **Exploratory Aim 2**, we will conduct an economic evaluation to determine the potential benefits of both arms the intervention based on and stated preferences (willingness to pay) and value of health improvements (difference in depression free days valued using Quality Adjusted Life Years conversion factor for depression) proceed ahead with a formal cost-effectiveness evaluation if justified.

ii. Rationale for Selection of Subject:

Why youth between 13 and 18 years old? This investigation is limited to adolescents in this age range because our prior experience working with youth and cognitive interventions indicates that children aged 12 and younger may not be able to cognitively grasp the abstract concepts of cognitive-behavioral therapy.[190] Moreover, depression risk has been shown to increase between ages 14 and 18.[191-192] Therefore, it makes the most sense to target the intervention to adolescents in this risk period.

Is an Attention Monitoring Psychoeducation comparison ethical and will it provide adequate attention control? There is currently no standard of care for adolescents who have depressive symptoms but who do not meet criteria for Major Depressive Disorder (MDD).[193] We believe that this study design is ethical because even adolescents in the HEALTH EDUCATION group will receive a higher standard of care than is traditionally available, including (1) screening, (2) psycho-education, (2) monitoring at 1 week and 1, 2, 6, 12, 18 and 24 months for progression to major depression, (3) feedback when progression occurs, (4) referral to the physician and family in completing treatment referrals, and (5) training of the primary care physician to recognize, treat and/or refer adolescents with major depression, consistent with primary care based Chronic Care Model interventions.[37, 194] By providing the HEALTH EDUCATION adolescents with a psycho-education Internet site, tracking assessment calls and physician encounters, we can actually measure "attention" so as to control for or evaluate effects in the mediation analysis. To address physician contact attention, all HEALTH EDUCATION physician contact will be documented. Furthermore, we estimate that the total study contact experienced by both groups when all assessment, PCP and Internet exposure are included to be 6.5 hours in CATCH-IT 3 and 5.0 hours in HEALTH EDUCATION.

Why exclude youth with no symptoms of depression, or with current depressive diagnoses? The goal of the present study is to reduce the risk of depressive episodes among adolescents who are at risk for disorder based on sub-syndromal depressed mood ("Indicated prevention" based on IOM[119] categorization of preventive interventions, presence of symptoms but not reaching criteria for disorder/episode). With regard to enrolling youth with no symptoms, past attempts to prevent depression using a "universal" prevention approach (e.g., targeting all adolescents in a particular school district) have not demonstrated benefit on the whole.[195] In terms of enrolling youth with current disorder, past research suggests that youth with current major depression are less likely to respond to prevention efforts than are "demoralized" youth (i.e., youth with elevated symptoms of depression but no current depressive disorder). [19] Sub-syndromal depressed mood represents a unique opportunity to intervene whereby adolescents are sufficiently "demoralized" to be motivated to participate, but not so depressed that the behavioral intervention may not be effective.[20, 38, 41]

Why include youth with other psychiatric diagnoses? The goal of the present study is to examine the efficacy of a *public health* model for the prevention of youth depression in primary care. Given our desire to implement this intervention in real-life settings, and given the high rates of comorbidity in youth with depressive disorders, it follows that a more inclusive recruitment approach is required. [10, 196-197]

Why over sample ethnic minority youth? Most interventions have been developed for European American adolescents. Sources of resiliency and vulnerability may vary by ethnicity in significant ways (see preliminary results).[36] We need a large enough cohort of ethnic minority youth to ensure we can adequately explore these issues. [24, 198-199]

Why a stream-lined assessment battery? The goal of this study is to examine the efficacy of an intervention with very limited person-to-person contact in realistic community settings with a population of primary care patients comparable to the general population of adolescents. We believe, based on our prior survey research, that extensive person-to-person psychological evaluation may alienate many potential study participants. We are thus only conducting a single face-to-face assessment meeting at baseline, and will conduct subsequent assessments via the telephone.

Why assess parents for depressive symptoms? Although the focus of the CATCH-IT 3 intervention is on preventing youth depression, for several reasons we are also assessing parental depressive symptoms. First, parental depression is a significant predictor of depression in adolescence.[200] Second, in a multi-site depression prevention study targeting at-risk adolescents, current parental depression was found to modify intervention effects, such that the intervention only decreased the incidence of depression in youth whose parents were not currently depressed.[131] Finally, studies of family-based prevention programs indicate that, in addition to decreasing risk for depression in the youth targeted by the intervention program, these prevention efforts also have decreased depressive symptom levels in parents.[201] We will assess depressive symptoms in parents at each time point and offer parents Internet based resources if they are experiencing depressed mood. We will also examine the moderating and mediating influence of parental mood on outcome.

Why a two-year follow-up? Effects from other prevention programs for youth depression have diminished over 22 months.[19] Also, effects from some prevention programs for youth depression have *increased* over time.[202-203] We will follow participants for 2 years after intake to detect both initial and more enduring aspects of the intervention. Extended follow-up is especially important in *prevention* outcome interventions, because by definition the participants enter this investigation when they are not in an episode of depression and must be followed prospectively to determine if the experimental intervention minimizes or eliminates future episodes.

Bo

g. Safety Monitoring and Assessment (if relevant, include provisions for managing adverse reactions) Data management (when relevant, address measures of privacy protection, coding, storage of information)

DSMB plan: A DSMB will convene every 6 months to review data. A report will be created by the analytic team led by Dr. Hendricks Brown. Stopping rules will include: 1) clear advantage in primary outcome in any arm; and 2) evidence of adverse events significantly above that expected in this risk population in either arm.

Risks of study and efforts to minimize risks:

General efforts to address/minimize risks: A Data and Safety Monitoring Board (DSMB) will monitor our sites and serve as a reporting body to the NIH as well as the respective IRBs. Data will be reviewed after each fifteen persons complete the study. The primary role of the DSMB will be to monitor the safety of participants in both the Internet-based preventive intervention and HEALTH EDUCATION comparison group, report any and all adverse effects in a timely and consistent fashion, and check the validity and integrity of the data. The DSMB will be composed of four members, none directly involved in the project or employed by the participating institutions, and will include one psychiatrist (with experience working with adolescents), one psychologist, one pediatrician and one internist.

The DSMB will meet first to approve the protocol and subsequently conduct annual reviews to determine whether patient safety has been adequately safeguarded and enrollment goals have been met. The DSMB will be kept apprised of any adverse events when they occur and will serve as the final arbiters of whether individual patients should be removed from the protocol. Although the therapists, in consultation with the clinically responsible PI and/or co-Investigator, are empowered to take whatever immediate action is necessary to safeguard the welfare of individual patients, the DSMB also will be called upon to render judgments in the advent of any serious clinical problems (e.g., serious suicidal intent or clinical deterioration).

In addition, to protect the privacy of our participants, we will apply for a Certificate of Confidentiality from the Department of Health and Human Services (DHHS) for this research study. Participants and their families will thus be protected from disclosure of confidential information in federal, state, or local civil, criminal, administrative, legislative, or other proceedings. Disclosure will be necessary, however, upon request of DHHS for audit or program evaluation purposes. **The Confidentiality Certificate does not prevent subjects from voluntarily releasing information about themselves or their involvement in this research. The investigators may not use the Certificate of Confidentiality to withhold information when a participant gives written permission to provide research information to others**.

Specific efforts to minimize risks. The proposed study poses minimal risks. Those risks that might exist fall into four categories: (a) risks of the preventive intervention; (b) risks associated with research assessments, consisting of face-to-face questions about depression, family and personal functioning, and other mental and emotional problems; (c) risks associated with potential loss of confidentiality; and (d) risks of worsening mental or emotional state. We address each in turn below.

A) Risks of the preventive intervention. The Internet-based preventive program consists of 14 modules that teens complete independently. In previous studies we did not find adverse effects of the Internet-based prevention program. However, there is the slight possibility that some teens may find it slightly uncomfortable to think about the feelings and attitudes that are highlighted in the Internet-based program.

Protection for risks of the preventive intervention: Youth participating in the Internet-based prevention program will be told that, if they experience any discomfort as a result of their participation, they should discuss any feelings of discomfort with members of the research staff. Research staff will talk with participants about the nature of their discomfort and will encourage them to take breaks or discontinue modules that they find uncomfortable. We will conduct safety surveillance calls for those in the CATCH-IT 3 arm at 1 week and 1, 2, 6, 12, and 24 months. Should we be unable to reach a participant at two consecutive time points, we will utilize public records or locator services to attempt additional contact to ensure the safety of the family.

B) *Risks associated with research assessments*: As noted, research assessments consist of face-to-face questions about depression, family and personal functioning, and other mental and emotional problems. Youth will give voluntary responses to interview questions; they are told that they can decline to answer any questions that they want, if answering would cause them distress or concern. We or our colleagues have used all of these instruments in previous research with no known problems or distress on the part of the participants. All research assessments are audio-recorded, for the purpose of review by senior research staff who make quality assurance ratings of staff performance. These audio-recordings are another form of research data that is at slight risk for unintentional release to unauthorized persons.

Protection for risks associated with research assessments: Youth and parents will be told that they are free to not respond or to terminate involvement at any time, with no adverse consequences. If a youth or parent appears to be distressed during assessments, research staff will halt the interview and offer to take a break. The interview will only recommence when and if the participant reports feeling capable of doing so. The interviews during the course of the study involve no specific risk or discomfort beyond those of a standard clinical interview. Interviewers will be trained to be sensitive to these issues.

C) Risks associated with potential loss of confidentiality. As noted above, there is a slight risk that research records (questionnaires, chart notes) might be obtained by persons not authorized to do so. There is a slight risk that research data files might be compromised and obtained or viewed by unauthorized persons. Finally, the strategy used to identify potential participants, screening questionnaires administered in the waiting room, carries the potential risk of loss of privacy. The risk of this method is that participants would experience a loss of confidentiality if others in the waiting room were to see their responses on the questionnaire form.
Protection for risks associated with potential loss of confidentiality. Data for all participants will be kept strictly confidential, except as mandated by law. All research files are kept in locked file cabinets or a locked file room. Participants will be assigned a numerical code for identification in the files. Names and other identifiers will be kept in separate locked files. Statistical analyses will be performed on aggregate-level data; participants are never individually named. All audio-recorded assessments and motivational interviews will be kept in locked file cabinets and will never be played for anyone who is not a member of the research team.

D) *Risks of worsening mental or emotional state and or self-harm thoughts/events.* Some youth enrolled in the Internet-based prevention program, and in the control group, will likely develop depression, suicidal behavior, and/or other mental and emotional problems during the study period. The development of suicidal ideation during the study remains as the most serious risk. However, these are risks inherent in the population and would occur whether or not they were enrolled in the study. We do not believe that the risk of these depressive, suicidal, or other adverse outcomes are increased as a function of being enrolled in this study. Should mood worsen or thoughts of self-harm develop (i.e. teen develop MDD, MDE, or suicidal thoughts), the teen's physician and parents will be notified. Physicians will be recontacted 1 week after disclosure to inquire as to whether the teen has begun treatment or a treatment plan is in place. Should no plan/treatment yet be established, PI or Project Manager will discuss with doctor the best course of action for the teen to ensure their safety and connection to mental health services.

Protection against risk of worsening mental or emotional state: Throughout the study, both intervention and control participants will be monitored regularly for evidence of symptom change. We will ask participants at each evaluation point whether they have or are experiencing negative emotions as a result of the intervention. Additionally, any individual with high sub-threshold depression (four symptoms as well as functional impairment) at evaluation points will be offered referral to a mental health specialist. If, during any assessment or intervention contact, research staff determine that either a youth or parent has developed a serious mental health problem (including but not limited to worsening depression, suicidal behavior, etc.), we will provide referral information and the telephone numbers to appropriate services ("usual care" for these conditions). If the detected problem is imminent and of crisis status, we will take appropriate immediate action, including escorting and/or providing medical transportation for these individuals to an emergency room. A Data Safety and Monitoring Board will be set up to monitor any adverse events that might occur during the course of the study (see description below).

Protection against risk of self-harm thoughts/acts: We have developed detailed procedures for detecting, assessing and triaging youth with psychiatric emergencies. The most frequent, although rare, of these crises involves suicidal behavior (not surprising given the depression risk of these youth) but includes acute substance abuse, psychotic symptoms, and so on. To address the risk of suicide we will conduct safety surveillance calls for those in the CATCH-IT 3 arm at 1, 4, 8 week and 6, 12, 18 and 24 months. We will also conduct post-study monitoring by telephone at 12 and 24 weeks. Monitoring during the intervention will include follow-up phone calls from the study coordinator four weeks after enrollment to assess for suicidal ideation and/or worsening depression symptoms. We will reassess for suicide risk and Major Depression at the exit interview. Because of our extensive and repeated assessment procedures, we believe we will be aware of suicidal thoughts/intentions, should they exist, and we are well prepared to manage these issues. In fact, given the high incidence of suicidal thinking among adolescents, it is likely that participants in this study with suicidal thoughts will be *more* likely to receive appropriate referrals, treatment and follow-up than will adolescents who

are not part of this research project. All participants who develop suicidal ideation will be referred for further evaluation by a child psychiatrist. The principal investigator will remain available on 24-hour call for adolescent participants or their parents should a need or concern arise. Briefly, any one of several “trigger events” (such as a positive response to a suicide assessment item, or suicidal behavior in sessions) requires staff to collect more detailed information about the possible crisis event. In the case of suicide, several specialized scales may be administered (the Suicide Intent Scale and the Suicide Ideation Scale), not for data but to aid in clinical decision-making. When sufficient information regarding the possible crisis is obtained, staff will use decision rules to decide whether the risk is (a) high and imminent or (b) high but not imminent. For the imminent risk situations they immediately link the subject to emergency mental health resources. This may include the use of security personnel to escort the youth to the appropriate facility, or enlisting parents to escort the youth. For events that are risky but not imminent, staff will contact the clinically-responsible investigators (the site PIs and co-investigators) for consultation. Follow-up for less-imminent risk events typically will include informing the parent(s) and other involved clinicians, and providing referrals. Because “usual care” is permitted in both study conditions, all youth will be referred to the usual crisis resources in their community. The intent of the research emergency protocol is not to offer more research intervention services, but to detect, assess, and triage/refer the youth to the most appropriate existing, non-research crisis resource available to them.

Self-harm prevention protocol: This protocol specifies assessment and management of risk during the study and includes structured assessment, risk stratification and actions to be taken.

PATH Abuse Protocol: A safety protocol has been developed to address any thoughts or actions of harm to others that are disclosed during any point of contact with the participant. Action will be taken to ensure immediate safety needs are met, followed by disclosure to parent and/or healthcare provider as necessary. Research staff will also provide assistance locating mental health services and notify any appropriate authorities in these circumstances.

Unique risks of a multi-site study: In a multi-site study of this type, some of the assessments and motivational interviews will be sent to the coordinating center for quality assurance (reliability and fidelity) ratings. These files, audio-recordings and other data will be identified only by the non-meaningful research ID (001, 002, etc.) and will not have any identifying information on it. Only authorized research staff at the other site will review these research audio-recordings, files or data, and then only for the purposes of quality assurance and/or research training. Data sent to remote sites such as the coordinating center also will be kept locked and secure at those sites, using the same procedures identified here in this section. All computerized data will be kept on the secured computers or networks at each site. These data will be accessible only to research staff, using confidential usernames and passwords.

Benefits of study:

The benefits of participation in this study include having extensive and repeated psychiatric assessments and a referral for treatment if there is a worsening of symptoms. For half of the sample, an Internet-based preventive intervention will be provided at no cost to the family. Both the adolescents and their parents likely will derive a sense of altruism, accomplishment, and contribution by furthering understanding of the problem through their participation. The intervention provided in this study may help to improve adolescents' academic, social, and family functioning and may prevent the development of substance abuse and lower the risk for suicidal behavior. In addition, adolescents and their parents will be paid for their participation.

Importance of the knowledge to be gained: The results of this study are important because they will help to develop the guidelines for preventive intervention with youth at risk for the development of depressive disorders, which is a major public mental health concern. The study addresses a pressing social need, the development and evaluation of low cost, evidence based, culturally acceptable and geographically accessible depression prevention interventions for youth. Furthermore, it addresses an important scientific question thus far not addressed in the depression prevention literature: how can youth at-risk for depression be motivated to participate in preventive interventions and change behaviors that may increase their risk? This study addresses this question directly by comparing a motivational approach that includes two primary interviews

and four motivational phone calls with a control condition that includes only a referral to the Internet-site (but with the same telephone monitoring).

Major Depression is a common disorder that causes significant morbidity, lost productivity and is associated with an increased risk of suicide. The first episode usually occurs in adolescence. There are currently no widely available, low cost, effective and acceptable depression prevention interventions for adolescents. The development of a feasible, cost-effective preventive intervention for this age group would be of great value.

Primary care physicians could provide a “motivational bridge” between at-risk adolescents and self-directed depression prevention programs. From the adolescent perspective, there is currently no systematic approach to connect them with self-directed depression prevention programs. Motivational interviewing can reduce at-risk behaviors in adolescents. In this intervention, the goal is to help the adolescent develop their own intrinsic motivation to reduce their risk of depression. The physician helps the adolescent identify how reducing their risk of depression is closely related to their valued life goals. While there is a strong theoretical rationale for a combined primary care/Internet-based intervention, we do not know whether this approach is feasible or would increase use of Internet-based depression prevention interventions.

Protection of Human Subjects Training:

The PI has already completed Human Subjects Training, and all research staff members will have completed such training by the start of the research.

h. Data management plan: The Chicago site will be the central data repository for the study, as it has successfully performed this function of a multi-site study for interview, self-administered and Internet data. [40, 129]Standard measures, including backup copies of paper instruments, locked storage, and secure data transfer will be used, as will encryption of electronic data, confidentiality protections and back-up archival storage.

8. For multi-site protocols, an overall study management plan should be provided.

Overview: Investigator time for this research is allocated at 35% at each site for the first four years, and 30% in year 5. Dr. Van Voorhees will oversee operations at the Chicago site; Dr. Gladstone will oversee operations at the Boston site. They will share responsibility and authority for leading and directing the project, and will share intellectual and logistic tasks. This will require regular weekly phone calls and frequent email contact between these phone calls, all of which they have done previously with success. They will both have the option to be included in publications derived from this project. They will make project decisions based on consensus and will seek external dispute resolution should it be required, although past experience suggests that no such steps will be necessary. Dr.s Van Voorhees and Gladstone have developed a collaborative relationship with Dr. Hendricks Brown, a nationally and internationally recognized expert in the evaluation of preventive mental health interventions and understanding trajectories and outcomes in adolescents, in order to address methodological aspects of the proposed research and to assist in data management and analysis. **Dr. Brown** will serve as a co-investigator on this project and will allocate 10% time in years 1-4, and 20% of time in year 5. He will have the option to be included in publications derived from this project. Dr. Brown will participate in regular phone calls, frequent email contact, and annual face-to-face meetings with **Dr.s Van Voorhees and Gladstone**. He will consult on project decisions. Decisions will be made by consensus, and external dispute resolution will be sought should conflicts arise. Dr. **Beardsee** and **Bell** will exercise roles as senior co-investigators at each site and collectively assisting in guiding the project. Local site leader investigators (**Eder, Canel, Chengand Desrosiers**) will lead implementation of the interventions at their sites.

<u>Evidence for successful implementation of shared PI roles</u>: <u>Multiple PD/PI Leadership Plan</u>. Drs. Van Voorhees and Gladstone have worked together for the past 6 years. They first began collaborating when Dr. Van Voorhees sought Dr. Gladstone’s assistance in developing a parent component to the CATCH-IT

intervention. Throughout the years they have worked together to address recruitment issues and to determine the best way to address family issues while preventing depression in at-risk adolescents. They also have met at various conferences to discuss CATCH-IT data, and they have collaborated on a risk study for adolescent depression. They have written papers collaboratively as well. In all these endeavors, they have functioned as a team, each contributing unique perspectives to the problem of preventing depression in at-risk adolescents. On the present investigation, they propose to continue their longstanding, successful collaboration by conducting a two-site randomized controlled trial of the CATCH-IT 3 prevention program.

Unique Contributions: Throughout the years, Dr. Van Voorhees and Dr. Gladstone have worked successfully as a research team without conflict. A multiple PD/PI approach is indicated because they each contribute unique skills relevant to this work, and because they share a commitment to the development of prevention approaches to address the problem of youth depression. Dr. Van Voorhees, a board certified internist and pediatrician, has developed the original CATCH-IT intervention and has unique experience in implementing an Internet-based intervention program in the primary care setting. He is a technology based researcher who has completed a previous study with funding from a K-08 award (and RWJ and NARSAD foundations) in 12 dispersed primary care sites; he also has expertise in health services research and in the influence of motivation and attitudes on behavior in adolescents. Dr. Gladstone, a licensed clinical psychologist and health services provider, is also a senior research scientist who currently serves as a PI on a large, NIMH-funded multi-site prevention trial for youth depression. Dr. Gladstone has considerable experience in the assessment of mental disorder outcomes and can ensure high quality assessments of study outcomes in a non-traditional study setting. She also has served as a Senior Research Associate on an NIMH-funded, family-based prevention study targeting the children of depressed parents and is in a unique position to implement the family-based component of the CATCH-IT prevention intervention. Dr. Gladstone has unique experience in the crucial developmental epoch of young adulthood and has many years' experience managing the tasks, both scientific and technical, of a large research project. Throughout the years, Drs. Van Voorhees and Gladstone have worked as a team and have benefited from one another's skills, backgrounds and unique perspectives. Together, they will be best able to conduct this research and to understand the implications of research findings for the design of future prevention interventions for adolescents and families facing depression.

Justification for multiple sites: The goal of this study is to determine if a primary care/technology based approach is efficacious in preventing adolescent depression in the full range of US practice environments.
(1) Recruit and implement protocol: Using two implementation sites will enable us to rapidly recruit a diverse sample and to over-sample low income ethnic minority youth rapidly. **Gladstone** and **Van Voorhees** have leveraged their relationships with local health systems to create a very large practice based research system.
(2) Interdisciplinary team model: Dr. **Van Voorhees** (Internist and Pediatrician) has considerable experience in primary care mental health services research while Dr. **Gladstone** (Clinical Psychologist) has taken leading roles in development and evaluation of family based depression prevention approaches, and in conducting a multi-site prevention study. Dr. **Brown** (social scientist) has extensive experience evaluating preventive interventions and understanding developmental pathways.
(3) Natural practice variations - valuable heterogeneity vs. unwanted heterogeneity: Chicago sites will focus on providers to low income minority youth while the Massachusetts site will focus on well-developed, staff model and physician hospital organizations with fully implemented electronic medical records systems. By incorporating site variations, we can evaluate the public health significance of this prevention approach without detracting from the goal of determining if the intervention is efficacious.
(4) Rapid accrual of results toward public health while minimizing costs: We currently have no reliable strategy for early intervention to pre-empt depression in community settings. Dr. **Van Voorhees**, working with

advisors/mentors **Bell**, **Gladstone** and **Beardslee**, was able to complete this during the first 2.5 years and will now begin the RO-1 funded study in year 5 of his K-award. By having each site conduct follow-up interviews for the other, we can reduce person power needs at each site.

(5) Minimizing study bias/maximizing study quality: By designing a 5-year efficacy study using two implementation sites (Chicago [**Van Voorhees**] and Boston [**Gladstone**]) and a separate data analytic site (Northwestern [**Brown**]), we can greatly improve study quality. Specifically, randomization will be conducted by the non-implementation site (Northwestern), full assessor blinding will be achieved by having interviews conducted by the alternate implementation site (e.g. Boston assesses outcomes for Chicago), and the non-implementation site (Northwestern) will conduct the main analyses.

Sources of research material:

The sources of research material will be the interviews with adolescents and parents, and questionnaires filled out by parents and teens, as well as by physicians, nurses and office staff. This material will be obtained for research purposes only. Each subject will be assigned a number that will be included on all data forms, and information linking this number to identifiable private information will be kept in a locked file cabinet, and will be stored on a computer in a password-protected file. This study does not involve access to medical or employee records. We will collect use information and ratings data from use of all Internet sites.

Measures to be used:

Please note that we will not include any suicide-related items on the questionnaires that are available for completion on the computer. Questions about suicidal thoughts/intentions will only be asked in person or over the phone so that any acute concerns can be addressed immediately.

1. *National Institute of Mental Health Strategic Goals.* Washington: National Institute of Mental Health 2007.

2. Reinherz HZ, Giaconia RM, Hauf AMC, Wasserman MS, Paradis AD. General and specific childhood risk factors for depression and drug disorders by early adulthood. *Journal of American Academic Child and Adolescent Psychiatry.* 2000;39(2):223-231.

3. Horwitz AV, White HR. Becoming married, depression, and alcohol problems among young adults. *J Health Soc Behav.* 1991;32(3):221-237.

4. Ernst C, Foldenyi M, Angst J. The Zurich Study: XXI. Sexual dysfunctions and disturbances in young adults. Data of a longitudinal epidemiological study. *Eur Arch Psychiatry Clin Neurosci.* 1993;243(3-4):179-188.

5. Binder J, Angst J. [Social consequences of psychic disturbances in the population: a field study on young adults (author's transl)]. *Arch Psychiatr Nervenkr.* 1981;229(4):355-370.

6. Skodol AE, Schwartz S, Dohrenwend BP, Levav I, Shrout PE. Minor depression in a cohort of young adults in Israel. *Archives of General Psychiatry.* Jul 1994;51(7):542-551.

7. Runeson B. Mental disorder in youth suicide. DSM-III-R Axes I and II. *Acta Psychiatr Scand.* May 1989;79(5):490-497.

8. Breslau N, Kilbey MM, Andreski P. DSM-III-R nicotine dependence in young adults: prevalence, correlates and associated psychiatric disorders. *Addiction.* Jun 1994;89(6):743-754.

9. Christie KA, Burke JD, Regier DA, Rae DS, et al. Epidemiologic evidence for early onset of mental disorders and higher risk of drug abuse in young adults. *Am J Psychiatry.* 1988;145(8):971-975.

10. Kessler RC, Walters EE. Epidemiology of DSM-III-R major depression and minor depression among adolescents and young adults in the National Comorbidity Survey. *Depress Anxiety.* 1998;7(1):3-14.

11. Glasgow RE, Vogt TM, Boles SM. Evaluating the public health impact of health promotion interventions: the RE-AIM framework. *Am J Public Health.* Sep 1999;89(9):1322-1327.

12. Poduska J, Kellam S, Brown CH, et al. Study protocol for a group randomized controlled trial of a classroom-based intervention aimed at preventing early risk factors for drug abuse: integrating effectiveness and implementation research. *Implement Sci.* 2009;4:56.

13. O'Connell ME, Boat TF, Warner KE. *Preventing mental, emotional, and behavioral disorders among young people : progress and possibilities*. Washington, D. C.: National Academies Press; 2009.

14. Clarke GN. *The coping wIth stress course adolescent: workbook*. Portland, OR: Kaiser Permanente Center for Health Research; 1994.

15. Jacobson NS MC, Dimdjian S. Behavioral Activation Treatment for Depression: Returning to Contextual Roots. *Clinical Psychology: Science and Practice.* Fall 2001;8(3):255-270.
16. Mufson L DK, Moreau D, Weissman MM. *Interpersonal Psychotherapy fo Depressed Adolescents*. New York, New York: Guilford Press; 2004.
17. Stuart S RM. *Interpersonal Psychotherapy A Clinicians Guide*. New York, New York: Oxford University Press; 2003.
18. Beardslee W, Gladstone TRG, Wright EW, Cooper AB. A family-based approach to the prevention of depressive symptoms in children at risk: Evidence of parental and child change. *Pediatrics.* 2003;112(2):E99-E111.
19. Clarke GN, Hornbrook M, Lynch F, et al. A randomized trial of a group cognitive intervention for preventing depression in adolescent offspring of depressed parents. *Arch Gen Psychiatry.* Dec 2001;58(12):1127-1134.
20. Garber J, Clarke GN, Weersing VR, et al. Prevention of depression in at-risk adolescents: a randomized controlled trial. *Jama.* Jun 3 2009;301(21):2215-2224.
21. Mufson L, Dorta KP, Wickramaratne P, Nomura Y, Olfson M, Weissman MM. A randomized effectiveness trial of interpersonal psychotherapy for depressed adolescents. *Arch Gen Psychiatry.* Jun 2004;61(6):577-584.
22. Beardslee WR, Gladstone TR, Wright EJ, Cooper AB. A family-based approach to the prevention of depressive symptoms in children at risk: evidence of parental and child change. *Pediatrics.* 2003;112(2):e119-131.
23. Mufson L, Weissman MM, Moreau D, Garfinkel R. Efficacy of interpersonal psychotherapy for depressed adolescents. *Archives of General Psychiatry.* 1999;56(6):573-579.
24. Bell CC, Bhana A, Petersen I, et al. Building protective factors to offset sexually risky behaviors among black youths: a randomized control trial. *J Natl Med Assoc.* Aug 2008;100(8):936-944.
25. Landback J, Prochaska M, Ellis J, et al. From prototype to product: development of a primary care/internet based depression prevention intervention for adolescents (CATCH-IT). *Community Ment Health J.* Oct 2009;45(5):349-354.
26. Van Voorhees B. Development and implementation of a primary care/internet-based depression prevention intervention for adolescents (CATCH-IT). *Journal of General Internal medicine.* 2008;23(s2):278.
27. O'Hara MW, Stuart S, Gorman LL, Wenzel A. Efficacy of interpersonal psychotherapy for postpartum depression. *Arch Gen Psychiatry.* 2000;57(11):1039-1045.
28. Clarke GN, Hawkins W, Murphy M, Sheeber LB, Lewinsohn PM, Seeley JR. Targeted prevention of unipolar depressive disorder in an at-risk sample of high school adolescents: a randomized trial of a group cognitive intervention. *Journal of American Academic Child and Adolescent Psychiatry.* Mar 1995;34(3):312-321.
29. Van Voorhees BW, Fogel J, Reinecke MA, et al. Randomized clinical trial of an Internet-based depression prevention program for adolescents (Project CATCH-IT) in primary care: 12-week outcomes. *J Dev Behav Pediatr.* Feb 2009;30(1):23-37.
30. Van Voorhees BW, Vanderplough-Booth K, Fogel J, et al. Integrative internet-based depression prevention for adolescents: a randomized clinical trial in primary care for vulnerability and protective factors. *J Can Acad Child Adolesc Psychiatry.* Nov 2008;17(4):184-196.
31. Lewinsohn PM, Roberts RE, Seeley JR, Rohde P, Gotlib IH, Hops H. Adolescent psychopathology: II. Psychosocial risk factors for depression. *J Abnorm Psychol.* May 1994;103(2):302-315.
32. Radloff LS. The use of the Center for Epidemiologic Studies Depression Scale in adolescents and young adults. *J Youth Adolesc.* 1991;20(2):149-166.
33. Van Voorhees BW, Paunesku D, Gollan J, Kuwabara S, Reinecke M, A B. Predicting future risk of depressive episode in adolescents: the Chicago Adolescent Depression Risk Assessment (CADRA).
Annals of Family Medicine 2008;6(6):503-511.
34. Lynch FL, Hornbrook M, Clarke GN, et al. Cost-effectiveness of an intervention to prevent depression in at-risk teens. *Arch Gen Psychiatry.* Nov 2005;62(11):1241-1248.
35. Matthews AK, Sanchez-Johnsen L, King A. Development of a Culturally Targeted Smoking Cessation Intervention for African American Smokers. *J Community Health.* Aug 29 2009.
36. Van Voorhees BW, Paunesku D, Fogel J, Bell CC. Differences in vulnerability factors for depressive episodes in African American and European American adolescents. *J Natl Med Assoc.* Dec 2009;101(12):1255-1267.
37. Van Voorhees BW, Walters AE, Prochaska M, Quinn MT. Reducing Health Disparities in Depressive Disorders Outcomes between Non-Hispanic Whites and Ethnic Minorities: A Call for Pragmatic Strategies over the Life Course. *Med Care Res Rev.* Aug 31 2007.
38. Van Voorhees BW, Ellis JM, Gollan JK, et al. Development and Process Evaluation of a Primary Care Internet-Based Intervention to Prevent Depression in Emerging Adults. *Prim Care Companion J Clin Psychiatry.* 2007;9(5):346-355.
39. Garber J. Depression in children and adolescents: linking risk research and prevention. *Am J Prev Med.* Dec 2006;31(6 Suppl 1):S104-125.
40. Van Voorhees B, Fogel J, Pomper B, R D. Adolescent Dose and Ratings of an Internet-Based Depression Prevention Program: A Randomized Trial of Primary Care Physician Brief Advice versus a Motivational Interview. *ournal of Cognitive and Behavioral Psychotherapies.* 2009;9(1).

41. Compas BE, Hinden BR, Gerhardt CA. Adolescent development: pathways and processes of risk and resilience. *Annu Rev Psychol.* 1995;46:265-293.

42. Lewinsohn PM, Joiner TE, Jr., Rohde P. Evaluation of cognitive diathesis-stress models in predicting major depressive disorder in adolescents. *J Abnorm Psychol.* May 2001;110(2):203-215.

43. Reinecke M, Simmons A. Vulnerability to DepressionAmong Adolescents: Implications for Cogntive Treatment. *Cognitive and Behavioral Practice.* 2005;12:166-176.

44. Kaufman J, Birmaher B, Brent D, et al. The Revised Schedule for Affective Disorders and Schizophrenia for School Aged Children: Present and lifetime version (K-SADS-PL): Preliminary reliability and validity data. *Journal of the American Academy of Child and Adolescent Psychiatry.* 1996;36:980-988.

45. Brent D, Kolko DJ, Wartella ME, Boylan MB. Adolescent psychiatry inpatients' risk of suicide attempt at 6-month follow-up. *Journal of the American Academy of Child and Adoelscent Psychiatry.* 1993;32:95-105.

46. Brent D, Perper J, Goldstein CE, et al. Risk factors for adolescent suicide: A comparison of adolescent suicide victims with suicidal inpatients. *Archives of General Psychiatry.* 1988;45:581-588.

47. Harrington R, Rudge H, Rutter M, Pickles A, Hill J. Adult outcomes of childhood and adolescent depresion. I: Psychiatric status. . *Archives of General Psychiatry.* 1990;47:465-473.

48. Georgiades K, Lewinsohn PM, Monroe SM, Seeley JR. Major depressive disorder in adolescence: the role of subthreshold symptoms. *J Am Acad Child Adolesc Psychiatry.* Aug 2006;45(8):936-944.

49. Weissman MM, Wolk S, Goldstein RB, et al. Depressed adolescents grown up. *Journal of the American Medical Association.* 1999;17:7-13.

50. Curry JF. Specific psychotherapies for child and adolescent depression. *Biological Psychiatry.* 2001;49:1091-1100.

51. Findling RL, Feeny NC, Stansbrey RJ, DelPorto-Bedoya D, Demeter C. Somatic treatment for depressive illnesses in children and adolescents. *Child Adolesc Psychiatr Clin N Am.* 2002;11(3):555-578, ix.

52. Emslie GJ, Rush AJ, Weinberg WA, Gullion CM, Rintelmann J, Hughes CW. Recurrence of major depressive disorder in hospitalized children and adolescents. *Journal of the American Academy of Child and Adoelscent Psychiatry.* 1997;36:785-792.

53. Brent D, Holder D, Kolko DJ, et al. A clinical psychotherapy trial for adolescent depression comparing cognitive, family, and supportive therapy. *Archives of General Psychiatry.* 1997;54:877-885.

54. Muthén B, Brown CH. Estimating drug effects in the presence of placebo response: Causal inference using growth mixture modeling. *Statistics in Medicine.* Forthcoming, 2009.

55. Druss BG, Hoff RA, Rosenheck RA. Underuse of antidepressants in major depression: prevalence and correlates in a national sample of young adults. *J Clin Psychiatry.* Mar 2000;61(3):234-237.

56. Young AS, Klap R, Sherbourne CD, Wells KB. The quality of care for depressive and anxiety disorders in the United States. *Archives of General Psychiatry.* Jan 2001;58(1):55-61.

57. Jaycox LH, Miranda J, Meredith LS, Duan N, Benjamin B, Wells K. Impact of a primary care quality improvement intervention on use of psychotherapy for depression. *Ment Health Serv Res.* 2003;5(2):109-120.

58. Cuijpers P, Brannmark JG, van Straten A. Psychological treatment of postpartum depression: A meta-analysis. *Journal of Clinical Psychology.* Jan 2008;64(1):103-118.

59. Cuijpers P, Muñoz RF, Clarke GN, Lewinsohn PM. Psychoeducational treatment and prevention of depression: The "Coping with Depression" course thirty years later. *Clinical Psychology Review.* Accepted, 2009.

60. Cuijpers P, van Straten A, Warmerdam L. Behavioral activation treatments of depression: A meta-analysis. *Clinical Psychology Review.* Apr 2007;27(3):318-326.

61. Muñoz RF, Cuijpers P, Smit F, Barrera A, Leykin Y. Prevention of onset and recurrence of major depression. *Annual Review of Clinical Psychology.* Accepted for publication (2009).

62. Van Voorhees B, Watson N, Bridges J, et al. Development and Pilot Study of a Marketing Strategy for Primary Care/Internet Based Depression Prevention Intervention for Adolescents (CATCH-IT). *Journal of Clinical Psychiatry Primary Care Companion.* in press.

63. Lewinsohn PM, Gotlib IH, Seeley JR. Adolescent psychopathology: IV. Specificity of psychosocial risk factors for depression and substance abuse in older adolescents. *J Am Acad Child Adolesc Psychiatry.* Sep 1995;34(9):1221-1229.

64. Lewinsohn PM, Gotlib IH, Seeley JR. Depression-related psychosocial variables: are they specific to depression in adolescents? *J Abnorm Psychol.* Aug 1997;106(3):365-375.

65. Liu YL. The role of perceived social support and dysfunctional attitudes in predicting Taiwanese adolescents' depressive tendency. *Adolescence.* Winter 2002;37(148):823-834.

66. Liu Y-L. The role of perceived social support and dysfunctional atttitudes in predicting Taiwanese adolescent's depressive tendency. *Adolescence.* 2002(Winter).

67. Hankin BL. Adolescent depression: description, causes, and interventions. *Epilepsy Behav.* Feb 2006;8(1):102-114.

68. Van Voorhees BW, Vanderploegbooth K, Fogel J, et al. Integrative Internet-Based Depression Prevention for Adolescents: A Randomized Clinical Trial in Primary Care for Vulnerability and Protective Factors. *Journal of the Canadian Academy of Child and Adolescent Psychiatry.* 2008(in press).

69. Van Voorhees BW, Paunesku D, Kuwabara SA, et al. Protective and vulnerability factors predicting new-onset depressive episode in a representative of U.S. adolescents. *J Adolesc Health.* Jun 2008;42(6):605-616.

70. Hollon SD, Evans MD, DeRubeis RJ. Cognitive mediation of relapse prevention following treatment for deprsesion: Implications of differential risk. In: Ingram RE, ed. *Psychological aspects of depression*. New York: Plenum Press; 1990:117-136.

71. Paunesku D, Ellis JM, Fogel J, et al. Clusters of Behaviors and Beliefs Predicting Adolescent Depression: Implications for Prevention

Journal of Cognitive and Behavioral Psychotherapies. 2008;8(2):147-168.

72. Vanderploeg-booth K, Puanesku, D, Msall, M, Van Voorhees, BW, . Using population attributable risk to help target preventive interventions for adolescent depression. *nternational Journal of Adolescent Medicine and Health.* (in press).

73. Evans DL, Foa EB, Gur RE, Hendin H, O'Brien CP, Seligman MEP. *Treating and preventing adolescent mental health disorders: What we know and what we don't know*. New York: Oxford University Press; 2005.

74. Procidano ME, Heller K. Measures of perceived social support from friends and from family: three validation studies. *Am J Community Psychol.* 1983;11(1):1-24.

75. Asarnow JR, Goldstein MJ, Tompson M, Guthrie D. One-year outcomes of depressive disorders in child psychiatric inpatients: Evaluation of the prognostic power of a brief measure of expressed emotion. *Journal of Child Psychology and Psychiatry.* 1993;34(129-137).

76. Kaufman J, Birmaher B, Brent DA, Ryan ND, Rao U. K-SADS-PL. *J Am Acad Child Adolesc Psychiatry.* 2000;39(10):1208.

77. Asarnow JR, Scott CV, Mintz J. A combined cognitive-behavioral family eduation intervention for depression in children: A treatment development study. *Cognitive Therapy and Research.* 2002;26:221-229.

78. Strober M, DeAntonio M, LHealth Educationrt C, Diamond J. Intensity and predictors of treatment received by adolescents with unipolar major depression prior to hospital admission. *Depress Anxiety.* 1998;7(1):40-46.

79. Shochet IM, Dadds MR, Holland D, Whitefield K, Harnett PH, Osgarby SM. The efficacy of a universal school-based program to prevent adolescent depression. *J Clin Child Psychol.* 2001;30(3):303-315.

80. Van Voorhees BW CL, Wang N, Rost KM, Rubenstein LV, Meridith L. Primary Care Patients with Depression Are Less Accepting of Treatment Than Those Seen by Mental Health Specialists: Results from a Primary Care Clinic Sample. *Journal of General Internal Medicine.* 2003;in press.

81. Gillham JE, Shatte AJ, Freres DR. Preventing depression: A review of cognitive-behavioral and family interventions. *Applied and Preventive Psychology.* 2000;9(2):63-88.

82. Crutzen R, de Nooijer J, Brouwer W, Oenema A, Brug J, de Vries NK. Internet-delivered interventions aimed at adolescents: a Delphi study on dissemination and exposure. *Health Educ Res.* Jun 2008;23(3):427-439.

83. Mains JA, Scogin FR. The effectiveness of self-administered treatments: a practice-friendly review of the research. *J Clin Psychol.* 2003;59(2):237-246.

84. Gray NJ, Klein JD, Cantrill JA, Noyce PR. Adolescent girls' use of the Internet for health information: issues beyond access. *J Med Syst.* 2002;26(6):545-553.

85. Merry S, McDowell H, Hetrick S, Bir J, Muller N. Psychological and/or educational interventions for the prevention of depression in children and adolescents. *Cochrane Database of Systematic Reviews.* 2004;1:CD003380.

86. Clarke GN. Adolescent use of web-based depression treatment programs. Portland, OR2002.

87. Christensen H, Griffiths KM, Korten A. Web-based cognitive behavior therapy: analysis of site usage and changes in depression and anxiety scores. *J Med Internet Res.* Jan-Mar 2002;4(1):E3.

88. Kaltenthaler E, Shackley P, Stevens K, Beverley C, Parry G, Chilcott J. A systematic review and economic evaluation of computerised cognitive behaviour therapy for depression and anxiety. *Health Technology Assessment.* 2002;6(22):1-89.

89. Cuijpers P, Van Straten A, Smit F. Preventing the incidence of new cases of mental disorders: a meta-analytic review. *J Nerv Ment Dis.* 2005;193(2):119-125.

90. Wantland DJ, Portillo CJ, Holzemer WL, Slaughter R, McGhee EM. The effectiveness of Web-based vs. non-Web-based interventions: a meta-analysis of behavioral change outcomes. *J Med Internet Res.* Nov 10 2004;6(4):e40.

91. van den Berg MH, Schoones JW, Vliet Vlieland TP. Internet-based physical activity interventions: a systematic review of the literature. *J Med Internet Res.* 2007;9(3):e26.

92. Walters ST, Wright JA, Shegog R. A review of computer and Internet-based interventions for smoking behavior. *Addict Behav.* Feb 2006;31(2):264-277.

93. Ybarra ML, Kiwanuka J, Emenyonu N, Bangsberg DR. Internet use among Ugandan adolescents: implications for HIV intervention. *PLoS Med.* Nov 2006;3(11):e433.

94. Lou CH, Zhao Q, Gao ES, Shah IH. Can the Internet be used effectively to provide sex education to young people in China? *J Adolesc Health.* Nov 2006;39(5):720-728.

95. Kirk SF, Harvey EL, McConnon A, et al. A randomised trial of an Internet weight control resource: the UK Weight Control Trial [ISRCTN58621669]. *BMC Health Serv Res.* Oct 29 2003;3(1):19.

96. Patten CA, Croghan IT, Meis TM, et al. Randomized clinical trial of an Internet-based versus brief office intervention for adolescent smoking cessation. *Patient Educ Couns.* Dec 2006;64(1-3):249-258.

97. Evers KE, Cummins CO, Prochaska JO, Prochaska JM. Online health behavior and disease management programs: are we ready for them? Are they ready for us? *J Med Internet Res.* Jul 1 2005;7(3):e27.

98. Verheijden MW, Jans MP, Hildebrandt VH, Hopman-Rock M. Rates and determinants of repeated participation in a web-based behavior change program for healthy body weight and healthy lifestyle. *J Med Internet Res.* 2007;9(1):e1.

99. Clarke G, Reid E, Eubanks D, et al. Overcoming depression on the Internet (ODIN): a randomized controlled trial of an Internet depression skills intervention program. *J Med Internet Res.* Dec 2002;4(3):E14.

100. Patten SB. Prevention of depressive symptoms through the use of distance technologies. *Psychiatr Serv.* 2003;54(3):396-398.

101. Santor DA, Poulin C, LeBlanc JC, Kusumakar V. Online health promotion, early identification of difficulties, and help seeking in young people. *J Am Acad Child Adolesc Psychiatry.* Jan 2007;46(1):50-59.

102. Fleming MF, Mundt MP, French MT, Manwell LB, Stauffacher EA, Barry KL. Brief physician advice for problem drinkers: long-term efficacy and benefit-cost analysis. *Alcohol Clin Exp Res.* Jan 2002;26(1):36-43.

103. Vansteenkiste M, Sheldon KM. There's nothing more practical than a good theory: integrating motivational interviewing and self-determination theory. *Br J Clin Psychol.* Mar 2006;45(Pt 1):63-82.

104. Miller W, Rollnick S. *Motivational Interviewing: Preparing People to Change Addictive Behaviors.* New York: Guilford Press; 1991.

105. DiClimente CC, Velasquez MM. Motivational Interviewing and the Stages of Change. In: Miller WR, Rollnick S, eds. *Motivational Interviewing: Preparing People for Change*. Second ed. New York: The Guilford Press; 2002:201-216.

106. Tevyaw TO, Monti PM. Motivational enhancement and other brief interventions for adolescent substance abuse: foundations, applications and evaluations. *Addiction.* 2004;99(Suppl 2):63-75.

107. Erol S, Erdogan S. Application of a stage based motivational interviewing approach to adolescent smoking cessation: The Transtheoretical Model-based study. *Patient Educ Couns.* Feb 25 2008.

108. McCambridge J, Strang J. The efficacy of single-session motivational interviewing in reducing drug consumption and perceptions of drug-related risk and harm among young people: results from a multi-site cluster randomized trial. *Addiction.* Jan 2004;99(1):39-52.

109. Feldstein SW, Forcehimes AA. Motivational interviewing with underage college drinkers: a preliminary look at the role of empathy and alliance. *Am J Drug Alcohol Abuse.* 2007;33(5):737-746.

110. Skaret E, Weinstein P, Kvale G, Raadal M. An intervention program to reduce dental avoidance behaviour among adolescents: a pilot study. *Eur J Paediatr Dent.* Dec 2003;4(4):191-196.

111. DiClemente CC, Prochaska JO. Processes and Stages of Self-Change: Coping and Competence in Smoking Behavior Change. In: Shiffman S, Wills TA, eds. *Coping and Substance Abuse*. Orlando: Academic Press, Inc; 1985.

112. Wing RR, Hamman RF, Bray GA, et al. Achieving weight and activity goals among diabetes prevention program lifestyle participants. *Obes Res.* 2004;12(9):1426-1434.

113. Johnson SB, Millstein SG. Prevention opportunities in health care settings. *Am Psychol.* 2003;58(6-7):475-481.

114. Joffe A, Radius S, Gall M. Health counseling for adolescents: what they want, what they get, and who gives it. *Pediatrics.* Sep 1988;82(3 Pt 2):481-485.

115. Emmons KM, Rollnick S. Motivational interviewing in health care settings. Opportunities and limitations. *Am J Prev Med.* Jan 2001;20(1):68-74.

116. St Peter RF, Reed MC, Kemper P, Blumenthal D. Changes in the scope of care provided by primary care physicians. *N Engl J Med.* Dec 23 1999;341(26):1980-1985.

117. Graeff-Martins AS, Flament MF, Fayyad J, Tyano S, Jensen P, Rohde LA. Diffusion of efficacious interventions for children and adolescents with mental health problems. *J Child Psychol Psychiatry.* Mar 2008;49(3):335-352.

118. Fremont WP, Nastasi R, Newman N, Roizen NJ. Comfort level of pediatricians and family medicine physicians diagnosing and treating child and adolescent psychiatric disorders. *Int J Psychiatry Med.* 2008;38(2):153-168.

119. Mrazek PJ, Haggerty RJ, eds. *Reducing risks for mental disorders: Frontiers for preventive research.* Washington, DC: National Academy Press.

1994.

120. Beardslee W, Gladstone TRG. Prevention of childhood depression: Recent findings and future prospects. *Biological Psychiatry.* 2001;49:1101-1110.

121. Gladstone TRG, Beardslee W. The prevention of depression in at-risk adolescents: Current and future directions. *Journal of Cognitive Psychotherapy.* 2000;14:9-23.

122. Gladstone TRG, Beardslee W. The prevention of depression in children and adolescents: A review. *Canadian Journal of Psychiatry.* in press.

123. Gladstone TRG, Kaslow NJ. Depression and attributions in children and adolescents: A meta-analytic review. *Journal of Abnormal Child Psychology.* 1995;23:597-606.

124. Gladstone TRG, Kaslow NJ, Seeley JR, Lewinsohn PM. Sex differences, attributional style, and depressive symptoms among adolescents. *Journal of Abnormal Child Psychology.* 1997;25:297-305.

125. Beardslee W, Wright EW, Gladstone TRG, Forbes P. Long-term effects from a randomized trial of two public health preventive interventions for parental depression. *Journal of Family Psychology.* 2007;21:703-713.

126. Van Voorhees BWE, Justin ; Gollan, Jackie K.; Bell, Carl C.; Stuart, Scott S.; Fogel, Joshua ; Corrigan,Patrick; Ford, Daniel E. . Development and Process Evaluation of a Primary Care Internet-Based Intervention to Prevent Depression in Young Adults. *Primary Care Companion Journal of Clinical Psychiatry.* 2007;9(5):346-355.

127. Jane-Llopis E, Hosman C, Jenkins R, Anderson P. Predictors of efficacy in depression prevention programmes. Meta-analysis. *Br J Psychiatry.* 2003;183:384-397.

128. Van Voorhees B. 12 Month Outcomes of a Randomized Clinical Trial of a Primary Care Internet Based Depression Prevention Intervention (CATCH-IT). Paper presented at: Society for Prevention Research 2009; Washington, DC.

129. Van Voorhees BW, Fogel J, Reinecke MA, et al. Randomized Clinical Trial of an Internet-Based Depression Prevention Program for Adolescents (Project CATCH-IT) in Primary Care: 12-Week Outcomes. *J Dev Behav Pediatr.* Feb 3 2008.

130. Marko M, Fogel J, E M, Van Voorhees B. Adolescent Internet Depression Prevention: Preferences for Intervention and Predictors of Intentions and Adherence. *Journal of Psyberpsychology and Rehabilitation.* in press.

131. Garber J, Clarke GN, Weersing VR, et al. Prevention of depression in at-risk adolescents: A randomized controlled trial. *Journal of the American Medical Association.* in press.

132. Beardslee WR, Gladstone TRG. Prevention of childhood depression: Recent findings and future prospects. *Biological Psychiatry.* 2001;49(12):1101-1110.

133. Brown C, Schulberg, H. C., Sacco, D., Perel, J. M., Houck, P. R. Effectiveness of treatments for major depression in primary medical care practice: a post hoc analysis of outcomes for African American and white patients. *J Affect Disord.* May 1999;53(2):185-192.

134. Brown C, Schulberg HC, Prigerson HG. Factors associated with symptomatic improvement and recovery from major depression in primary care patients. *Gen Hosp Psychiatry.* Jul-Aug 2000;22(4):242-250.

135. Brown CH, Wang W, Kellam SG, et al. Methods for testing theory and evaluating impact in randomized field trials: intent-to-treat analyses for integrating the perspectives of person, place, and time. *Drug Alcohol Depend.* Jun 1 2008;95 Suppl 1:S74-S104.

136. Pierson HM, Hayes SC, Gifford EV, et al. An examination of the Motivational Interviewing Treatment Integrity code. *J Subst Abuse Treat.* Jan 2007;32(1):11-17.

137. Moyers TB, Martin T, Manuel JK, Hendrickson SM, Miller WR. Assessing competence in the use of motivational interviewing. *J Subst Abuse Treat.* Jan 2005;28(1):19-26.

138. Radloff LS. The CES-D scale: a self-report depression scale for research in the general population. *Applied Psychological Measures.* 1977;1:385-401.

139. Johnson JG, Harris ES, Spitzer RL, Williams JB. The patient health questionnaire for adolescents: validation of an instrument for the assessment of mental disorders among adolescent primary care patients. *J Adolesc Health.* Mar 2002;30(3):196-204.

140. Van Voorhees BW, Melkonian S, Marko, M.,, Humensky J, Fogel J. Adolescents in primary care with sub-threshold depressed mood screened for participation in a depression prevention study: Co-morbidity and factors associated with depressive symptoms. *The Open Psychiatry Journal,*.4:10-18.

141. Pastore DR, Juszczak L, Fisher MM, Friedman SB. School-based health center utilization: a survey of users and nonusers. *Arch Pediatr Adolesc Med.* 1998;152(8):763-767.

142. Piantadosi S. Clinical trials : a methodologic perspective. *Applied probability and statistics ;; 1346; Variation: Wiley series in probability and statistics.; Applied probability and statistics ;; 1346.* 1997; http://www.loc.gov/catdir/description/wiley037/96046311.htmlAvailability: Check the catalogs in your library. Libraries worldwide that own item: 172 Search the catalog at University of South FloridaFind Items About: Clinical trials :2,483
Materials specified: Publisher description http://www.loc.gov/catdir/description/wiley037/96046311.htmlMaterials specified: Table of contents http://www.loc.gov/catdir/toc/wiley022/96046311.html.

143. Gagne RM BL, Wager WW. *Principles of Instructional Design*. Fort Worth, TX: Harcourt Brace Jovanovich College Publishers; 1992.

144. Van Voorhees BW, Watson N, Bridges J. Development and pilot study of a marketing strategy for primary care/internet based depression prevention intervention for adolescents (CATCH-IT). *Journal of Clinical Psychiatry Primary Care Companion.* in press.

145. Spence SR, M. Cognitive approaches to understanding, preventing, and treating child and adolescent depression. In: Reinecke MaC, D., ed. *ognitive therapy across the lifespan: Evidence and practice*. Cambridge, UK: Cambridge University Press.; 2003.

146. The Diabetes Prevention Program (DPP): description of lifestyle intervention. *Diabetes Care.* 2002;25(12):2165-2171.

147. Katon W, Rutter C, Ludman EJ, et al. A randomized trial of relapse prevention of depression in primary care. *Arch Gen Psychiatry.* Mar 2001;58(3):241-247.

148. Unutzer J. IMPACT Study Results. Paper presented at: Robert Wood Johnson Foundation Depression in Primary Care Annual Meeting2004.

149. Christensen H, Griffiths KM, Jorm AF. Delivering interventions for depression by using the internet: randomised controlled trial. *Bmj.* 2004;328(7434):265. Epub 2004 Jan 2023.

150. Christensen H, Griffiths KM, Mackinnon AJ, Brittliffe K. Online randomized controlled trial of brief and full cognitive behaviour therapy for depression. *Psychol Med.* Dec 2006;36(12):1737-1746.

151. Schoener EP, Madeja CL, Henderson MJ, Ondersma SJ, Janisse JJ. Effects of motivational interviewing training on mental health therapist behavior. *Drug Alcohol Depend.* May 20 2006;82(3):269-275.

152. Van Voorhees BW, Fogel J, Houston TK, Cooper LA, Wang NY, Ford DE. Beliefs and attitudes associated with the intention to not accept the diagnosis of depression among young adults. *Annals of Family Medicine.* 2005;3(1):38-46.

153. Van Voorhees BW, Fogel J, Houston TK, Cooper LA, Wang NY, Ford DE. Attitudes and illness factors associated with low perceived need for depression treatment among young adults. *Soc Psychiatry Psychiatr Epidemiol.* Sep 2006;41(9):746-754.

154. Keller MB, Lavori PW, Friedman B, et al. The Longitudinal Interval Follow-up Evaluation. A comprehensive method for assessing outcome in prospective longitudinal studies. *Arch Gen Psychiatry.* Jun 1987;44(6):540-548.

155. Rost KM, Duan N, Rubenstein LV, et al. The Quality Improvement for Depression collaboration: general analytic strategies for a coordinated study of quality improvement in depression care. *Gen Hosp Psychiatry.* Sep-Oct 2001;23(5):239-253.

156. Birmaher B, Khetarpal S, Brent D, et al. The screen for child anxiety related emotional disorders (SCARED): Scale construction and psychometric characteristics. *Journal of the American Academy of Child and Adoelscent Psychiatry.* 1997;36:545-553.

157. Pelham WE, Jr., Gnagy EM, Greenslade KE, Milich R. Teacher ratings of DSM-III-R symptoms for the disruptive behavior disorders. *J Am Acad Child Adolesc Psychiatry.* Mar 1992;31(2):210-218.

158. Kendall PC HB, Hays RC,. Self-referent speech and psychopathology: The balance of positive and negative thinking. *Cognitive Therapy and Research.* 1989;13:583-598.

159. Kaufman NK, Rohde P, Seeley JR, Clarke GN, Stice E. Potential mediators of cognitive-behavioral therapy for adolescents with comorbid major depression and conduct disorder. *J Consult Clin Psychol.* 2005;73(1):38-46.

160. Beck AT, Steer RA. *Manual for the Beck Hopelessness Scale*. San Antonio, TX: The Psychological Corporation; 1988.

161. Knight JR, Shrier LA, Bravender TD, Farrell M, Vander Bilt J, Shaffer HJ. A new brief screen for adolescent substance abuse. *Arch Pediatr Adolesc Med.* Jun 1999;153(6):591-596.

162. Hankin BL, Abramson LY. Measuring cognitive vulnerability to depression in adolescence: reliability, validity, and gender differences. *J Clin Child Adolesc Psychol.* Dec 2002;31(4):491-504.

163. Masten AS, Coatsworth JD, Neemann J, Gest SD, Tellegen A, Garmezy N. The structure and coherence of competence from childhood through adolescence. *Child Dev.* 1995;66(6):1635-1659.

164. Van Voorhees B. Adolescent attitudes toward depression prevention. *Journal of General Internal medicine.* 2008;23(s2):243.

165. B.H. Ascher B, Farmer EMZ, Burns BJ, Angold A. The Child and Adolescent Services Assessment (CASA): Description and Psychometrics. *Journal of Emotional and Behavioral Disorders.* 1996;4(12-20).

166. Hollingshead A. *Four-factor index of social status*. New Haven: Yale University; 1975.

167. Brown CH, Wang W, Kellam SG, et al. Methods for testing theory and evaluating impact in randomized field trials: Intent-to-treat analyses for integrating the perspectives of person, place, and time. *Drug and Alcohol Dependence.* Jun 2008;95(Suppl 1):S74-S104; Supplementary data associated with this article can be found, in the online version, at doi:110.1016/j.drugalcdep.2008.1001.1005.

168. Petras H, Kellam SG, Brown CH, Muthén BO, Ialongo NS, Poduska JM. Developmental epidemiological courses leading to antisocial personality disorder and violent and criminal behavior: Effects by young adulthood of a universal preventive intervention in first- and second-grade classrooms. *Drug and Alcohol Dependence.* Jun 2008;95(Suppl1):S45-S59.

169. Muthén B. Growth modeling with binary responses. In: Eye Av, Clogg CC, eds. *Categorical variables in developmental research : methods of analysis*. San Diego: Academic Press; 1996:37-54.

170. Muthén B, Asparouhov T. Modeling interactions between latent and observed continuous variables using maximum-likelihood estimation In Mplus. *Mplus Web Notes.* March 5 2003;Version 1(6):10 p.

171. Muthén BO, Khoo S-T. Longitudinal studies of achievement growth using latent variable modeling. *Learning & Individual Differences.* 1998;10(2):73-101.

172. Gibbons RD. Mixed-effects models for mental health services research *Health Services and Outcomes Research Methodology.* 2000;1(2):91-129.

173. Gibbons RD, Hedeker D, Elkin I, et al. Some conceptual and statistical issues in analysis of longitudinal psychiatric data: Application to the NIMH Treatment of Depression Collaborative Research Program dataset. *Archives of General Psychiatry.* 1993;50(9):739-750.

174. Fava GA, Ruini C, Rafanelli C, Finos L, Conti S, Grandi S. Six-year outcome of cognitive behavior therapy for prevention of recurrent depression. *Am J Psychiatry.* 2004;161(10):1872-1876.

175. Siddique J, Brown CH, Hedeke D, et al. Missing data in longitudinal trials – Part B, Analytic issues. *Psychiatric Annals.* 2008;38(12):793-801.

176. Siddiqui O, Ali MW. A comparison of the random-effects pattern mixture model with last-observation-carried-forward (LOCF) analysis in longitudinal clinical trials with dropouts. *Journal of Biopharmaceutical Statistics.* Nov 1998;8(4):545-563.

177. Muthén B. Latent variable analysis: Growth mixture modeling and related techniques for longitudinal data. In: Kaplan D, ed. *The Sage handbook of quantitative methodology for the social sciences*. Thousand Oaks, CA: Sage Publications; 2004:345-368.

178. Muthén BO. The potential of growth mixture modelling. *Infant and Child Development.* Nov-Dec 2006;15(6):623-625.

179. Muthén BO, Asparouhov T. Growth mixture modeling: Analysis with non-Gaussian random effects. In: Fitzmaurice G, Davidian M, Verbeke G, Molenberghs G, eds. *Longitudinal data analysis: A handbook of modern statistical methods*. Boca Raton, Fl: Chapman & Hall/CRC Press; 2008:143-165.

180. Muthén BO, Brown CH, Masyn K, et al. General growth mixture modeling for randomized preventive interventions. *Biostatistics.* 2002;3:459-475.

181. Muthén BO, Brown CH, Leuchter A, Hunter A. General approaches to analysis of course: Applying growth mixture modeling to randomized trials of depression medication. In: Shrout PE, ed. *Causality and psychopathology: Finding the determinants of disorders and their cures*. Washington, DC: American Psychiatric Publishing; Forthcoming, 2009.

182. Nylund KL, Asparouhov T, Muthén BO. Deciding on the number of classes in latent class analysis and growth mixture modeling: A Monte Carlo simulation study. *Structural Equation Modeling.* 2007;14(4):535-569.

183. MacKinnon DP, Lockwood CM, Brown CH, Wang W, Hoffman JM. The intermediate endpoint effect in logistic and probit regression. *Clinical Trials.* 2007;4(5):499-513.

184. MacKinnon DP. *Introduction to statistical mediation analysis*. Mahwah, NJ: Lawrence Erlbaum Associates; 2008.

185. Jo B. Estimation of intervention effects with noncompliance: Alternative model specifications. *Journal of Educational & Behavioral Statistics.* 2002;27(4):385-409.

186. Jo B, Ginexi EM, Ialongo NS. Handling missing data in randomized experiments with noncompliance. 2009, Conditionally accepted for publication.

187. Jo B, Muthén BO. Modeling of intervention effects with noncompliance: A latent variable approach for randomized trials. In: Marcoulides GA, Schumacker RE, eds. *New developments and techniques in structural equation modeling.* Hillsdale, NJ: Lawrence Erlbaum Associates; 2001:57-87.

188. Jo B, Vinokur A. Sensitivity analysis and bounding of causal effects with alternative Identifying assumptions. *Journal of Educational and Behavioral Statistics.* 2009, In press.

189. Kraemer HC, Mintz J, Noda A, Tinklenberg J, Yesavage JA. Caution regarding the use of pilot studies to guide power calculations for study proposals. *Archives of General Psychiatry.* May 2006;63(5):484-489.

190. Butler L, Mietzitis S, Friedman R, Cole E. The effect of two school-based intervention programs on depressive symptoms in preadolescents. *Amerian Educational Research Journal.* 1980;17:111-119.

191. Hankin BL, Abramson LY, Mofitt TE, Silva PA, McGee R, Angell KE. Development of depression from preadolescence to young adulthood: Emerging gender differences in a 10-year longitudinal study. *Journal of Abnormal Psychology.* 1998;107:128-140.

192. Klerman GL, Budman S, Berwick D, et al. Efficacy of a brief psychosocial intervention for symptoms of stress and distress among patients in primary care. *Med Care.* Nov 1987;25(11):1078-1088.

193. Van Voorhees BW, Smith S, Ewigman B. Treat depressed teens with medication and psychotherapy. *J Fam Pract.* Nov 2008;57(11):735-739a.

194. Asarnow JR, Jaycox LH, Duan N, et al. Effectiveness of a quality improvement intervention for adolescent depression in primary care clinics: a randomized controlled trial. *Jama.* Jan 19 2005;293(3):311-319.

195. Horowitz JL, Garber J. The prevention of depressive symptoms in children and adolescents: A meta-analytic review. *Journal of Consulting and Clinical Psychology.* 2006;74:401-415.

196. Kessler RC, Nelson CB, McGonagle KA, Liu J, Swartz M, Blazer DG. Comorbidity of DSM-III-R major depressive disorder in the general population: results from the US National Comorbidity Survey. *Br J Psychiatry Suppl.* Jun 1996(30):17-30.

197. Lewinsohn PM, Hops H, Roberts RE, Seeley JR, Andrews JA. Adolescent psychopathology: I. Prevalence and incidence of depression and other DSM-III-R disorders in high school students. *J Abnorm Psychol.* Feb 1993;102(1):133-144.

198. Bell CC. Cultivating resiliency in youth. *J Adolesc Health.* 2001;29(5):375-381.

199. Bell CC RJ, Blount MA. . Preventing Violence: Research and Evidence-Based Intervention Strategies. In: Lutzker J, ed. *Suicide Prevention*. Washington, DC: American Psychological Association; 2005:p. 217 - 237.

200. Beardslee W, Versage E, Gladstone TRG. Children of affectively ill parents: A review of the past 10 years. *Journal of the American Academy of Child and Adoelscent Psychiatry.* 1998;37:1134-1141.

201. Compas BE, Forehand R, Keller G. Cognitive-behavioral prevention in families of depressed parents: 12-month outcomes of a randomized trial. *Society for Research in Child Development*. Denver, CO2009.

202. Sandler IN, Ayers TS, Wolchik SA, Tien J, Kwok O, Haine RA. The Family Bereavement Program: Efficacy evaluation of a theory-based prevention program for parentally bereaved children and adolescents. *Journal of Consulting and Clinical Psychology.* 2003;71:587-600.

203. Wolchik SA, Sandler IN, Milsap RE, Plummer BA, Greene SM, Anderson ER. Six-year follow-up of preventive interventions for children of divorce: A randomized clinical trial. *Journal of the American Medical Association.* 2002;288:1874-1881.
