# Supplementary material for: Effect of Internet-Based Cognitive Behavioral Humanistic and Interpersonal Training vs Internet-Based General Health Education on Adolescent Depression in Primary Care: A Randomized Clinical Trial
Source: JAMA Netw Open. 2018 Nov 2;1(7):e184278. doi: 10.1001/jamanetworkopen.2018.4278 (PMC6286074; doi:10.1001/jamanetworkopen.2018.4278)
Supplement: Supplement 2. — eTable 1. Definition for Criteria for Main Outcomes Analyses eTable 2. MI Fidelity: Mean MITI Summary Scores eTable 3. Hazard Ratio Estimate and 95% CI for First Depressive Episode Comparing CATCH-IT to Health Education eTable 4. Incidence of First Depressive Episode by Treatment Group eTable 5. Adjusted Cox Proportional Hazard Model Results, Including Adolescent Baseline CES-D10 as a Moderator eTable 6. Depressed Mood eTable 7. Functional Status eTable 8. A, Participants With Missing Data: KSADS and CES-D10. B, Predictors of Missing 2-Month Follow-up: Time to Last Assessment <2 Months. C, Predictors of Missing 6-Month Follow-up: Time to Last Assessment <6 Months. D, Predictors of Missing 2-Month CES-D10. E, Predictors of Missing 6-Month CES-D10 [file jamanetwopen-1-e184278-s002.pdf]

Supplementary Online Content

Gladstone TRG, Terrizzi DA, Paulson A, et al. Effect of internet-based cognitive behavioral humanistic and interpersonal training vs internet-based general health education on adolescent depression in primary care: a randomized clinical trial. *JAMA Netw Open*. 2018;1(7):e184278. doi:10.1001/jamanetworkopen.2018.4278

eTable 1. Definition for Criteria for Main Outcomes Analyses
eTable 2. MI Fidelity: Mean MITI Summary Scores
eTable 3. Hazard Ratio Estimate and 95% CI for First Depressive Episode Comparing CATCH-IT to Health Education
eTable 4. Incidence of First Depressive Episode by Treatment Group
eTable 5. Adjusted Cox Proportional Hazard Model Results, Including Adolescent Baseline CES-D_{10} as a Moderator
eTable 6. Depressed Mood
eTable 7. Functional Status
eTable 8. A, Participants With Missing Data: KSADS and CES-D_{10}. B, Predictors of Missing 2-Month Follow-up: Time to Last Assessment <2 Months. C, Predictors of Missing 6-Month Follow-up: Time to Last Assessment < 6 Months. D, Predictors of Missing 2-Month CES-D_{10}. E, Predictors of Missing 6-Month CES-D_{10}

This supplementary material has been provided by the authors to give readers additional information about their work.

© 2018 Gladstone TRG et al. *JAMA Network Open*.

eTable 1. Definition of Criteria for Main Outcomes Analyses

**Type of Analysis**	**Level of Participation**	**Percent of Sample (CATCH-IT/ Health Ed)**	**Justification**
Intention to treat	All randomized participants	N=369 (100%)<br>193/176<br>100%/100%	
Modified intention to treat	Completed MI 1 on timely basis (2 months)	N=356 (96%)<br>180/176<br>93%/100%	(1) Because of the nature of the intervention, there may be time to first dose effects; postulates that goal orientation is a key protective factor for adolescent depression[60].<br>(2) The 12-month outcomes of the CATCH-IT study[48] suggest benefit from MI over and above the internet program. In this trial, the lag between eligibility assessment and MI was rarely more than 10 days. [48]
As treated	Went to the website (parent or adolescent)	N=294 (80%)<br>157/137<br>81%/78%	Internet program thought to be the primary intervention. Those with no exposure may actually worsen.[56] In the primary care Psychobabble study, the principal aim of the intervention was to have primary care patients minimally engage in the internet portal.[61]
Per protocol 2	At least 2 modules completed (parent or adolescent)	N=245 (66%)<br>122/123<br>63%/70%	Dose response curves suggest completion of 2-4 modules is minimum threshold for benefit; mean number of modules completed in Hong Kong trial was 3.0 and demonstrated benefit[61] In 2.5 year outcome of CATCH-IT, data suggest that experience of "self-efficacy" in even one module category correlates with decline in CES-D score 2.5 years later.[62] In a dosing study of an adolescent supportive accountability model, adolescents completed 22% of didactics, or the equivalent of 2.75 modules.[63]
Per protocol 4	At least 4 modules	N=222 (60%)<br>104/118	Dose response curves suggest completion of 3-5 modules yields

© 2018 Gladstone TRG et al. *JAMA Network Open*.

	completed (parent or adolescent)	54%/67%	moderate benefit (at 60 minutes) [56]. In SPARX trial, 86% completed 4 modules[64]. In Vets trial, 54% of sample completed 4 modules[65].
Per protocol 7	At least 7 modules completed (parent or adolescent)	N=152 (41%)<br>59/93<br>31%/53%	Dose response curves suggest leveling off benefit after 120 minutes (6-7 modules) in Hong Kong CATCH-IT trial, where 14% completed 7 or more modules.[56] In 2.5 year study of risk factors for CATCH-IT, the mean modules completed was 6.75; this relates directly to the mean number of minutes and characters typed in the current trial.[66]

© 2018 Gladstone TRG et al. *JAMA Network Open*.

eTable 2. MI Fidelity: Mean MITI Summary Scores

	**All (N=32)**		**Baseline (N=17)**		**2 months (N=15)**		
	**Mean**	**(SD)**	**Mean**	**(SD)**	**Mean**	**(SD)**	**p[a]**
Technical Global (1-5)	3.0	(0.5)	3.0	(0.3)	2.9	(0.6)	0.34
Relational Global (1-5)	2.9	(0.6)	3.0	(0.5)	2.9	(0.8)	0.60
%CR	25.7	(24.2)	24.3	(22.3)	27.3	(26.9)	0.73
R:Q	0.5	(0.3)	0.5	(0.4)	0.4	(0.2)	0.48
Total MI-Adherent	0.4	(0.7)	0.6	(0.9)	0.2	(0.4)	0.14
Total MI-Non-Adherent	0.9	(1.3)	0.7	(1.2)	1.1	(1.5)	0.49
MI length, min[b]							
Mean	7.7	(4.0)	7.5	(4.7)	8.0	(3.2)	0.73
Median	6.9		6.6		7.6		

[a]Tests for difference between baseline and 2-month MIs: pooled variance t-tests.
[b]N=28 for MI length.
Definitions:
Technical Global: (cultivating change talk+softening sustain talk)/2, 1-5, higher is better
Relational Global: (partnership+empathy)/2, 1-5, higher is better
% CR: 100*complex reflection/(simple reflection+complex reflection)
Reflection to Question Ratio: total reflections (simple+complex) / total questions
Total MI-adherent: seeking collaboration+affirm+emphasizing autonomy
Total MI non-adherent: confront+persuade

© 2018 Gladstone TRG et al. *JAMA Network Open*.

eTable 3. Hazard Ratio Estimate and 95% CI for First Depressive Episode Comparing CATCH-IT to Health Education

		**Unadjusted**					**Adjusted[a]**				
		**HR**	**95% CI**		**p-value**	**Person-weeks**	**HR**	**95% CI**		**p-value**	**Person-weeks**
Intent to Treat (N = 369)	DSR ≥ 3	0.59	(0.27,	1.29)	0.18	7617.29	0.53	(0.23,	1.23)	0.14	7617.29
	DSR ≥ 4	0.64	(0.21,	1.96)	0.44	7836.00	0.71	(0.22,	2.24)	0.56	7836.00
	DSR = 5	0.79	(0.18,	3.53)	0.76	7904.43	0.76	(0.17,	3.40)	0.72	7904.43
Modified Intent to Treat (N = 356)	DSR ≥ 3	0.61	(0.28,	1.33)	0.21	7512.43	0.56	(0.24,	1.30)	0.18	7512.43
	DSR ≥ 4	0.66	(0.22,	2.02)	0.47	7731.14	0.75	(0.24,	2.37)	0.63	7731.14
	DSR = 5	0.81	(0.18,	3.63)	0.78	7799.57	0.81	(0.18,	3.62)	0.78	7799.57
As Treated (N = 294)	DSR ≥ 3	0.54	(0.25,	1.19)	0.13	6489.71	0.49	(0.21,	1.12)	0.09	6489.71
	DSR ≥ 4	0.59	(0.19,	1.81)	0.36	6708.43	0.64	(0.20,	2.02)	0.44	6708.43
	DSR = 5	0.73	(0.16,	3.27)	0.68	6776.86	0.73	(0.16,	3.34)	0.69	6776.86
Per Protocol 2 (N = 245)	DSR ≥ 3	0.41	(0.17,	0.99)	0.047	5678.86	0.44	(0.18,	1.08)	0.07	5678.86
	DSR ≥ 4	0.64	(0.21,	1.96)	0.44	5865.43	0.71	(0.23,	2.27)	0.57	5865.43
	DSR = 5	0.79	(0.18,	3.52)	0.75	5933.86	0.80	(0.18,	3.66)	0.78	5933.86
Per Protocol 4 (N = 222)	DSR ≥ 3	0.41	(0.16,	1.06)	0.07	5165.29	0.42	(0.16,	1.10)	0.08	5165.29
	DSR ≥ 4	0.57	(0.17,	1.89)	0.36	5345.57	0.63	(0.18,	2.16)	0.46	5345.57
	DSR = 5	0.87	(0.20,	3.90)	0.86	5412.86	0.89	(0.19,	4.15)	0.88	5412.86
Per Protocol 7 (N = 152)	DSR ≥ 3	0.72	(0.25,	2.06)	0.53	3514.86	0.61	(0.20,	1.86)	0.38	3514.86
	DSR ≥ 4	0.96	(0.23,	4.01)	0.95	3668.57	1.03	(0.23,	4.71)	0.97	3668.57
	DSR = 5	1.08	(0.18,	6.46)	0.93	3714.29	2.63	(0.23,	29.71)	0.43	3714.29

[a]Adjusted for gender, race (white vs. non-white), ethnicity (Hispanic vs. non-Hispanic), age at baseline, site, and baseline CES-D_{10} score. Participants with no baseline CES-D_{10} score were excluded from the adjusted analyses (N=7).

© 2018 Gladstone TRG et al. *JAMA Network Open*.

eTable 4. Incidence of First Depressive Episode by Treatment Group

		Health Ed				CATCH-IT			
		**N**	**Events**	**Person-weeks**	**IR[a]**	**N**	**Events**	**Person-weeks**	**IR[a]**
Intent to Treat (N = 369)	DSR >= 3	176	17	3850.71	44.15	193	10	3766.57	26.55
	DSR >= 4		8	3994.71	20.03		5	3841.29	13.02
	DSR = 5		4	4054.86	9.865		3	3849.57	7.793
Modified Intent to Treat (N = 356)	DSR >= 3	176	17	3850.71	44.15	180	10	3661.71	27.31
	DSR >= 4		8	3994.71	20.03		5	3736.43	13.38
	DSR = 5		4	4054.86	9.865		3	3744.71	8.011
As Treated (N = 294)	DSR >= 3	137	17	3139.86	54.14	157	10	3349.86	29.85
	DSR >= 4		8	3283.86	24.36		5	3424.57	14.6
	DSR = 5		4	3344.00	11.96		3	3432.86	8.739
Per Protocol 2 (N = 245)	DSR >= 3	123	17	2834.71	59.97	122	7	2844.14	24.61
	DSR >= 4		8	2978.71	26.86		5	2886.71	17.32
	DSR = 5		4	3038.86	13.16		3	2895.00	10.36
Per Protocol 4 (N = 222)	DSR >= 3	118	16	2709.71	59.05	104	6	2455.57	24.43
	DSR >= 4		8	2847.43	28.1		4	2498.14	16.01
	DSR = 5		4	2907.57	13.76		3	2505.29	11.97
Per Protocol 7 (N = 152)	DSR >= 3	93	11	2150.14	51.16	59	5	1364.71	36.64
	DSR >= 4		5	2261.29	22.11		3	1407.29	21.32
	DSR = 5		3	2299.86	13.04		2	1414.43	14.14

[a]Incidence rate (IR): events per 10,000 person-weeks.

© 2018 Gladstone TRG et al. *JAMA Network Open*.

eTable 5. Adjusted Cox Proportional Hazard Model Results, Including Adolescent Baseline CES-D_{10}[a] as a Moderator

**Model Estimates**		**Beta**	**HR**	**95% CI**		**p-value**
CATCH-IT vs. HE		1.36	3.89	(0.50,	30.54)	0.20
Male vs. Female		-0.49	0.61	(0.24,	1.58)	0.31
Non-white vs. white		0.54	1.72	(0.74,	3.99)	0.21
Hispanic vs. not		0.08	1.08	(0.39,	2.97)	0.88
Age at baseline		-0.29	0.75	(0.54,	1.03)	0.08
Site (Boston vs. Chicago)		1.07	2.90	(1.21,	6.95)	0.02
Baseline CES-D_{10}		0.15	1.16	(1.02,	1.32)	0.02
CATCH-IT * CES-D_{10} interaction		-0.20	0.82	(0.67,	0.99)	0.04
**Between Group Comparisons, comparing CATCH-IT to HE, for increasing CES-D_{10} scores**	**CES-D_{10} score**	**Beta**	**HR**	**95% CI**		**p-value**
	5	0.36	1.44	(0.41,	5.03)	0.84
	10	-0.64	0.53	(0.22,	1.29)	0.34
	15	-1.63	0.20	(0.05,	0.77)	0.05
	20	-2.63	0.07	(0.01,	0.66)	0.05
**Within Group Comparison, describing a one-unit increase in CES-D_{10} score**	**CES-D_{10} score**	**Beta**	**HR**	**95% CI**		**p-value**
CATCH-IT	1 unit change	-0.05	0.95	(0.82,	1.10)	0.75
HE	1 unit change	0.15	1.16	(1.02,	1.32)	0.04

[a] Higher CES-D_{10} scores indicate higher levels of depression. Participants with no baseline CES-D_{10} score were excluded from the analysis (N=7).

© 2018 Gladstone TRG et al. *JAMA Network Open*.

eTable 6. Depressed Mood

	CATCH-IT (N=193)			Health Ed (N=176)			Entire sample (N=369)		
CES-D_{10} (0-30)	**N**	**Mean**	**SD**	**N**	**Mean**	**SD**	**N**	**Mean**	**SD**
Screening	187	10.6	(4.1)	173	10.5	(4.1)	360	10.6	(4.1)
Baseline	190	9.5	(4.5)	172	9.4	(4.6)	362	9.4	(4.6)
2 months	123	9.2	(4.2)	140	7.9	(5.2)	263	8.5	(4.8)
6 months	116	8.2	(4.9)	133	7.9	(4.7)	249	8.0	(4.8)

CATCH-IT estimated slope: -0.183, p=0.01
Health Ed estimated slope: -0.184, p=0.008
Between-group difference (group*visit interaction): p=0.99
From linear mixed effect growth model with random effects for intercept and slope, adjusted for sex, ethnicity (Hispanic, non-Hispanic), race (white, non-white), baseline age, and site. The screening CES-D was not included in the model.

© 2018 Gladstone TRG et al. *JAMA Network Open*.

eTable 7. Functional Status

	**CATCH-IT (N=193)**			**Health Ed (N=176)**			**Entire sample (N=369)**		
GAS (1-100)	**N**	**Mean**	**SD**	**N**	**Mean**	**SD**	**N**	**Mean**	**SD**
Baseline	193	78.3	(9.3)	174	78.0	(9.6)	367	78.1	(9.4)
2 months	114	83.4	(8.6)	135	82.8	(11.5)	249	83.1	(10.3)
6 months	126	84.6	(8.9)	136	84.5	(10.2)	262	84.5	(9.6)

CATCH-IT estimated slope: 0.969, p<0.001
Health Education estimated slope: 0.945, p<0.001
Between-group difference (group*visit interaction): p=0.90
From linear mixed effect growth model with random effects for intercept and slope, adjusted for sex, ethnicity (Hispanic, non-Hispanic), race (white, non-white), baseline age, site, and baseline teen CES-D_{10}.

© 2018 Gladstone TRG et al. *JAMA Network Open*.

eTable 8A-E.

eTable 8A. Participants With Missing Data: KSADS and CES-D_{10}

	**All**		**CATCH-IT**		**Health Ed**		
	**N**	**(%)**	**N**	**(%)**	**N**	**(%)**	**p[a]**
Time to last follow-up[b]							
< 2 months	57	(15%)	41	(21%)	16	(9%)	0.001
≥ 2 months	312	(85%)	152	(79%)	160	(91%)	
< 6 months	71	(19%)	48	(25%)	23	(13%)	0.004
≥ 6 months	298	(81%)	145	(75%)	153	(87%)	
CES-D_{10}							
Missing 2-month CES-D_{10}	106	(29%)	70	(36%)	36	(20%)	<0.001
Has 2-month CES-D_{10}	263	(71%)	123	(64%)	140	(80%)	
Missing 6-month CES-D_{10}	120	(33%)	77	(40%)	43	(24%)	0.002
Has 6-month CES-D_{10}	249	(67%)	116	(60%)	133	(76%)	

[a] From chi-square test for difference between CATCH-IT and HealthEd.

[b] All available follow-up assessments (KSADS) were used to calculate time observed.

© 2018 Gladstone TRG et al. *JAMA Network Open*.

eTable 8B. Predictors[a] of Missing 2-Month Follow-up: Time to Last Assessment <2 Months

	**OR**	**(95% CI)**		**b**	**p**
Intercept	--			-4.71	0.007
Boston (ref=Chicago)	0.24	(0.10,	0.57)	-1.44	0.001
CATCH-IT (ref=Health Ed)	3.03	(1.56,	5.88)	1.11	0.001
Age at baseline, y	1.16	(0.95,	1.42)	0.15	0.15
Male (ref=female)	1.34	(0.70,	2.56)	0.29	0.38
Hispanic (ref=non-Hisp)	0.78	(0.35,	1.71)	-0.25	0.53
Non-white (ref=white)	1.34	(0.69,	2.61)	0.30	0.39
Mother's education (ref=college graduate)					
HS graduate/GED or less	3.19	(1.39,	7.30)	1.16	0.006
Some college	1.18	(0.52,	2.68)	0.16	0.70
Parents' marital status (ref=married)					
Never married	0.67	(0.26,	1.69)	-0.41	0.39
Divorced, separated, widowed	0.47	(0.20,	1.12)	-0.75	0.09
Firstborn child (ref=not firstborn)	1.46	(0.78,	2.75)	0.38	0.24
Past episode at baseline (ref=no past episode)	1.15	(0.58,	2.29)	0.14	0.70
High CES-D at screening or baseline (ref=no high CES-D)	0.89	(0.32,	2.50)	-0.11	0.83

[a]From logistic regression model. Some observations were excluded from the model due to missing data for covariates: N=353.

© 2018 Gladstone TRG et al. *JAMA Network Open*.

eTable 8C. Predictors[a] of Missing 6-Month Follow-up: Time to Last Assessment < 6 Months

	**OR**	**(95% CI)**		**b**	**p**
Intercept				-5.55	<0.001
Boston (ref=Chicago)	0.20	(0.09,	0.46)	-1.61	<0.001
CATCH-IT (ref=Health Ed)	2.62	(1.43,	4.79)	0.96	0.002
Age at baseline, y	1.23	(1.02,	1.49)	0.21	0.03
Male (ref=female)	1.57	(0.86,	2.86)	0.45	0.14
Hispanic (ref=non-Hisp)	0.61	(0.28,	1.29)	-0.50	0.19
Non-white (ref=white)	1.37	(0.73,	2.56)	0.31	0.32
Mother's education (ref=college graduate)					
HS graduate/GED or less	2.99	(1.37,	6.53)	1.09	0.006
Some college	1.03	(0.48,	2.23)	0.03	0.93
Parents' marital status (ref=married)					
Never married	0.95	(0.41,	2.19)	-0.06	0.90
Divorced, separated, widowed	0.49	(0.22,	1.09)	-0.72	0.08
Firstborn child (ref=not firstborn)	1.51	(0.84,	2.71)	0.41	0.17
Past episode at baseline (ref=no past episode)	1.36	(0.72,	2.57)	0.31	0.34
High CES-D at screening or baseline (ref=no high CES-D)	1.11	(0.42,	2.91)	0.10	0.84

[a]From logistic regression model. Some observations were excluded from the model due to missing data for covariates: N=353.

© 2018 Gladstone TRG et al. *JAMA Network Open*.

eTable 8D. Predictors[a] of Missing 2-Month CES-D_{10}

	OR	(95% CI)		b	p
Intercept	--			-1.93	0.16
Boston (ref=Chicago)	0.72	(0.41,	1.27)	-0.33	0.26
CATCH-IT (ref=Health Ed)	2.58	(1.56,	4.28)	0.95	<0.001
Age at baseline, y	1.03	(0.87,	1.21)	0.02	0.77
Male (ref=female)	1.46	(0.88,	2.44)	0.38	0.15
Hispanic (ref=non-Hisp)	1.10	(0.59,	2.05)	0.10	0.76
Non-white (ref=white)	1.45	(0.85,	2.46)	0.37	0.17
Mother's education (ref=college graduate)					
HS graduate/GED or less	2.79	(1.40,	5.55)	1.03	0.004
Some college	2.02	(1.09,	3.72)	0.70	0.02
Parents' marital status (ref=married)					
Never married	0.77	(0.37,	1.62)	-0.26	0.50
Divorced, separated, widowed	0.96	(0.52,	1.77)	-0.05	0.88
Firstborn child (ref=not firstborn)	1.11	(0.68,	1.83)	0.11	0.68
Past episode at baseline (ref=no past episode)	0.88	(0.51,	1.53)	-0.13	0.65
High CES-D at screening or baseline (ref=no high CES-D)	0.63	(0.29,	1.40)	-0.46	0.26

[a]From logistic regression model. Some observations were excluded from the model due to missing data for covariates: N=353.

© 2018 Gladstone TRG et al. *JAMA Network Open*.

eTable 8E. Predictors[a] of Missing 6-Month CES-D_{10}

	OR	(95% CI)		b	p
Intercept	--			-5.84	<0.001
Boston (ref=Chicago)	0.55	(0.31,	0.96)	-0.61	0.04
CATCH-IT (ref=Health Ed)	2.31	(1.41,	3.78)	0.84	<0.001
Age at baseline, y	1.30	(1.11,	1.53)	0.26	0.001
Male (ref=female)	1.08	(0.65,	1.81)	0.08	0.77
Hispanic (ref=non-Hisp)	0.63	(0.34,	1.19)	-0.46	0.16
Non-white (ref=white)	1.29	(0.76,	2.17)	0.25	0.34
Mother's education (ref=college graduate)					
HS graduate/GED or less	2.34	(1.18,	4.64)	0.85	0.02
Some college	1.75	(0.96,	3.20)	0.56	0.07
Parents' marital status (ref=married)					
Never married	1.11	(0.54,	2.28)	0.10	0.78
Divorced, separated, widowed	1.08	(0.59,	1.99)	0.08	0.80
Firstborn child (ref=not firstborn)	1.33	(0.81,	2.18)	0.29	0.25
Past episode at baseline (ref=no past episode)	0.74	(0.43,	1.28)	-0.30	0.28
High CES-D at screening or baseline (ref=no high CES-D)	1.48	(0.64,	3.43)	0.39	0.37

[a]From logistic regression model. Some observations were excluded from the model due to missing data for covariates: N=353.

© 2018 Gladstone TRG et al. *JAMA Network Open*.
