# Supplementary material for: Effect of Internet-Based Cognitive Behavioral Humanistic and Interpersonal Training vs Internet-Based General Health Education on Adolescent Depression in Primary Care: A Randomized Clinical Trial
Source: JAMA Netw Open. 2018 Nov 2;1(7):e184278. doi: 10.1001/jamanetworkopen.2018.4278 (PMC6286074; doi:10.1001/jamanetworkopen.2018.4278)
Supplement: Supplement 3. — Data Sharing Statement [file jamanetwopen-1-e184278-s003.pdf]

Data Sharing Statement

Gladstone. Effect of Internet-Based Cognitive Behavioral Humanistic and Interpersonal Training vs Internet-Based General Health Education on Adolescent Depression in Primary Care. *JAMA Netw Open*. Published November 02, 2018. 10.1001/jamanetworkopen.2018.4278

Data
Data available: Yes
Data types: Deidentified participant data
How to access data: bvanvoor@uic.edu; mmarko@uic.edu
When available: beginning date: 09-01-2019

Supporting Documents
Document types: None

Additional Information
Who can access the data: Researchers whose proposed use of the data has been approved and upon the review and approval of the Institutional Review Board (IRB) of record
Types of analyses: For specified purpose only
Mechanisms of data availability: With a signed data access agreement
Any additional restrictions: Any data will be available for research purposes only, once the proposed request has been reviewed and approved for the specific reason listed in the proposal, by the IRB of record (UIC IRB)
